# Supplementary material for: Comparative impact assessment of COVID-19 policy interventions in five South Asian countries using reported and estimated unreported death counts during 2020-2021
Source: PLOS Glob Public Health. 2023 Dec 27;3(12):e0002063. doi: 10.1371/journal.pgph.0002063 (PMC10752546; doi:10.1371/journal.pgph.0002063)
Supplement: S1 Text — The details of modeling true number of infections due to COVID-19. (PDF) [file pgph.0002063.s001.pdf]

Supplementary Section

S1 Infection Modeling

Priors for initial seedings

We seeded the transmission model with six sequential days of an equal number of infections, $c_{1,m} = c_{2,m} = ... = c_{6,m} \sim \text{Exponential}(\frac{1}{\tau})$ where $\tau \sim \text{Exp}(1)$. The prior parameter was chosen based on model convergence. For the counterfactual death analysis such a prior assumption is reasonable since it is a relative analysis. Its impact is analogous to what we observed when varying the prior values of the Infection Fatality Rate (IFR) mean in the Sensitivity Analysis (as discussed in the Results Section). Nonetheless, the posterior estimates of $c_{t,m}$ obtained on the basis of this assumption, particularly for the year 2021, are deemed unreliable. Consequently, we have adopted an indirect approach to address this issue.

During the period of 2020-2021, four national serosurveys were conducted in India to estimate the true prevalence of COVID cases in India. These serosurveys provided estimates of Under Reporting Factor for cases ($\text{URF}_{\text{cases}}$) where,

$$\text{URF}_{\text{cases}} = \frac{\text{Cumulative number of true infections}}{\text{Cumulative number of reported infections}}$$

in India, specifically 29.1 (May-June 2020), 14.3 (August-September 2020), 25.7 (December 2020- January 2021) and 24.9 (June-July 2021). The denominator of $\text{URF}_{\text{cases}}$ is readily available from JHU CSSE COVID database. With the help of sero estimates we obtain estimates of the numerator for India at the end of each serosurvey period. We predicted the $\text{URF}_{\text{cases}}$ at other dates for India in the period of March 15, 2020 - Dec 31, 2021 using linear interpolations with knots at the known serosurvey end dates. In terms of the notation of the transmission model, the numerator in $\text{URF}_{\text{cases}}$ at day t is equal to $\sum_{\tau=0}^{t} c_{\tau,m}$ where m stands for India. Using the model estimates of $c_{\tau,m}$ and the ones obtained from serosurveys, we calculated the factor by which the model infection estimates needed to be adjusted to match the serosurvey estimates for India. To obtain the estimates of $\text{URF}_{\text{cases}}$ for the other four countries, we made an assumption that the adjusting factor obtained for India remained the same for the other countries as well. We report these modified estimates of $\text{URF}_{\text{cases}}$ for 2020 and 2021 in Supplementary Tables S5 and S6 respectively. All these estimates are obtained using reported deaths analysis only.

In 2020, Pakistan had the highest $\text{URF}_{\text{cases}}$ of 45.4 [27.2, 86.9] on December 31, 2020. In contrast, Sri Lanka and Nepal had consistently lower URF compared to the other three countries. In 2021, except Sri Lanka all the other four countries had comparable $\text{URF}_{\text{cases}}$ around 25-30. Throughout this work, our main focus is on comparing the five countries based on their performances in the counterfactual scenarios in terms of mortality. The $\text{URF}_{\text{cases}}$ estimates provided in Supplementary Tables S5 and S6 should be used with caution since these are based on several assumptions.
