# Supplementary figures and images for: Comparative impact assessment of COVID-19 policy interventions in five South Asian countries using reported and estimated unreported death counts during 2020-2021
Source: PLOS Glob Public Health. 2023 Dec 27;3(12):e0002063. doi: 10.1371/journal.pgph.0002063 (PMC10752546; doi:10.1371/journal.pgph.0002063)

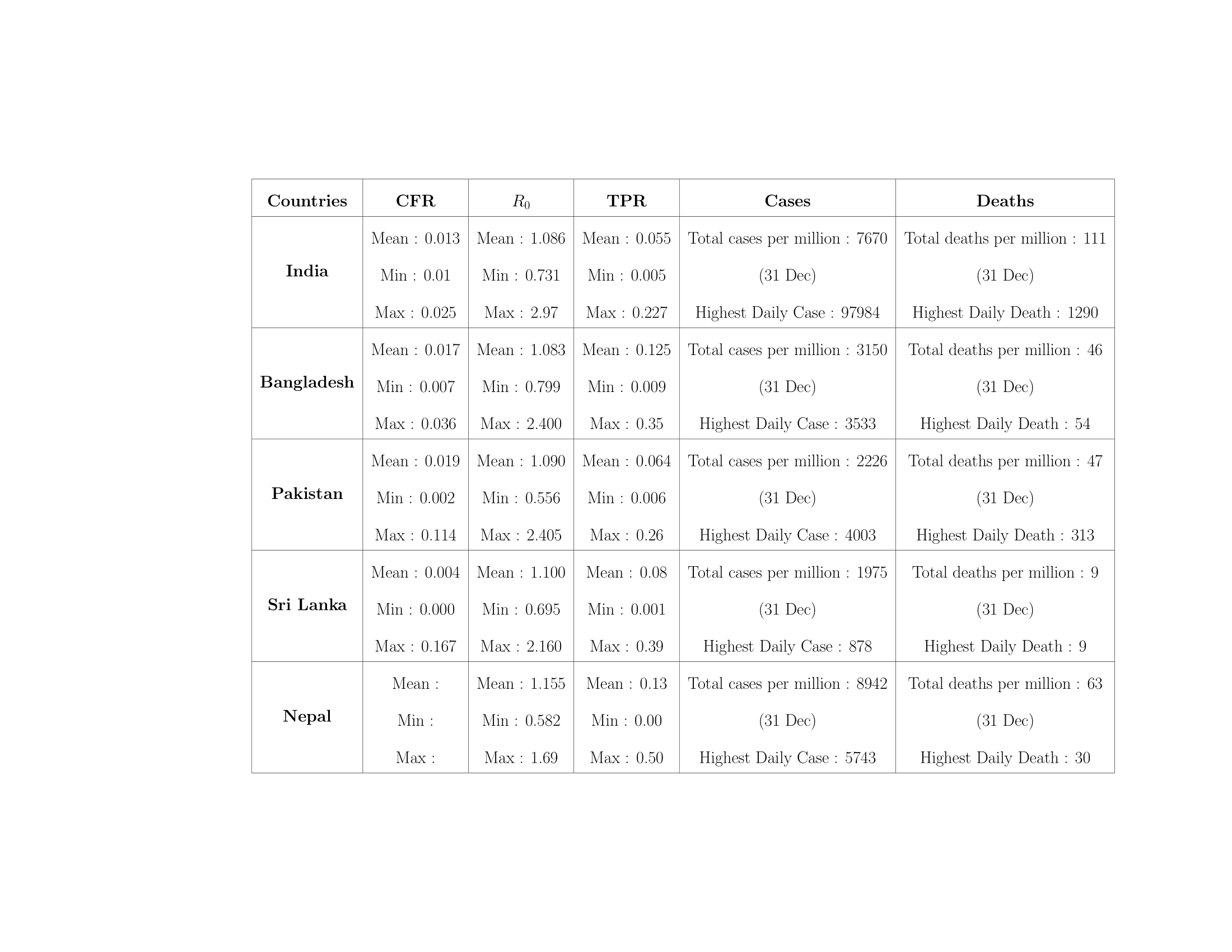

Supplement: S1 Table — CFR, R0 and TPR denote Case Fatality Rate, Basic Reproduction Number and Test Positive Rate respectively. For CFR and TPR, mean, min, and max of the daily CFR or TPR in the period March 15, 2020–December 31, 2020 reported. For cases and deaths, total counts per million reported on December 31, 2020. (TIF) [file pgph.0002063.s002.tif]

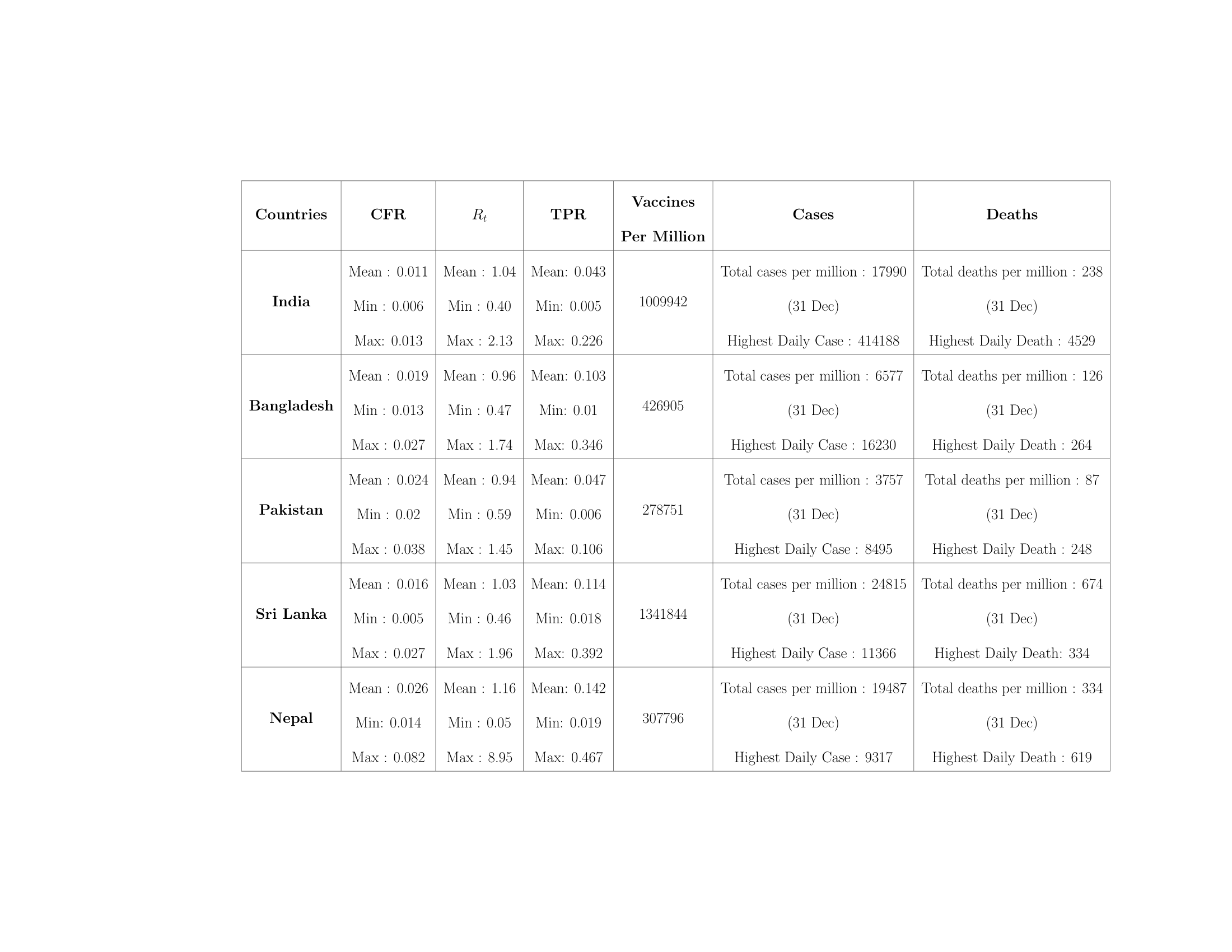

Supplement: S2 Table — CFR, R0, and TPR denote Case Fatality Rate, Time Varying Reproduction Number, and Test Positive Rate respectively. For CFR (or TPR), mean, minimum, and maximum of the daily CFR (or TPR) during the period January 1, 2021–December 31, 2021, are reported. Cumulative number of vaccines per million during the period January 1, 2021 to December 31, 2021 are reported. For cases (or deaths), the cumulative counts per million are obtained using the cumulative number of cases (or deaths) for the whole of 2021. (TIF) [file pgph.0002063.s003.tif]

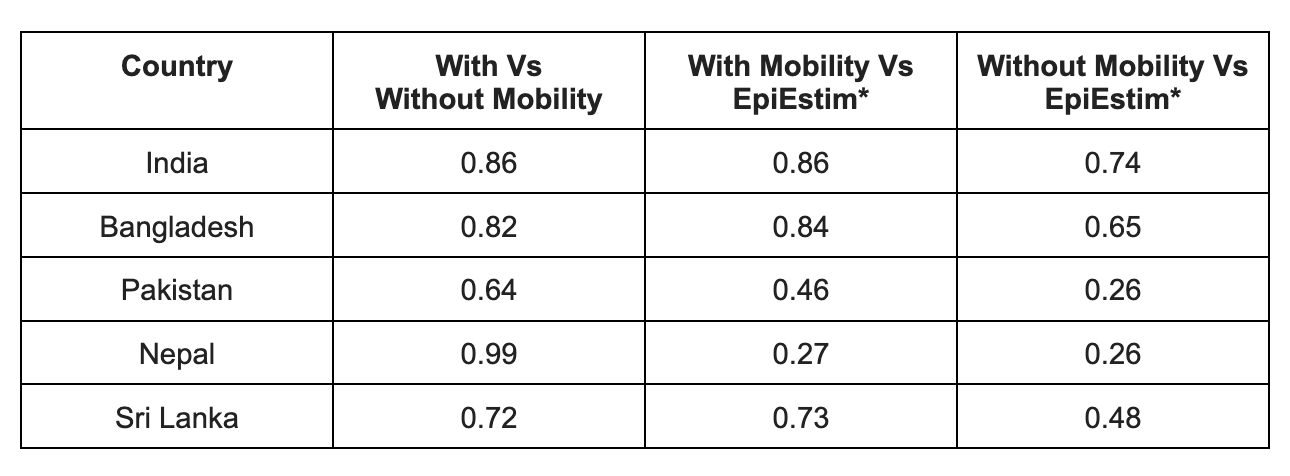

Supplement: S3 Table — Estimated Rt,m series are obtained using three ways: Not including mobility (original analysis- only based on reported mortality data), including mobility into the model and finally using the R Package EpiEstim which calculates Rt,m based on COVID-19 reported cases data. EpiEstim using the method “parametric_si” and specifying the mean and sd (4 days and 1 respectively) for the Delta strain of COVID-19. (PNG) [file pgph.0002063.s004.png]

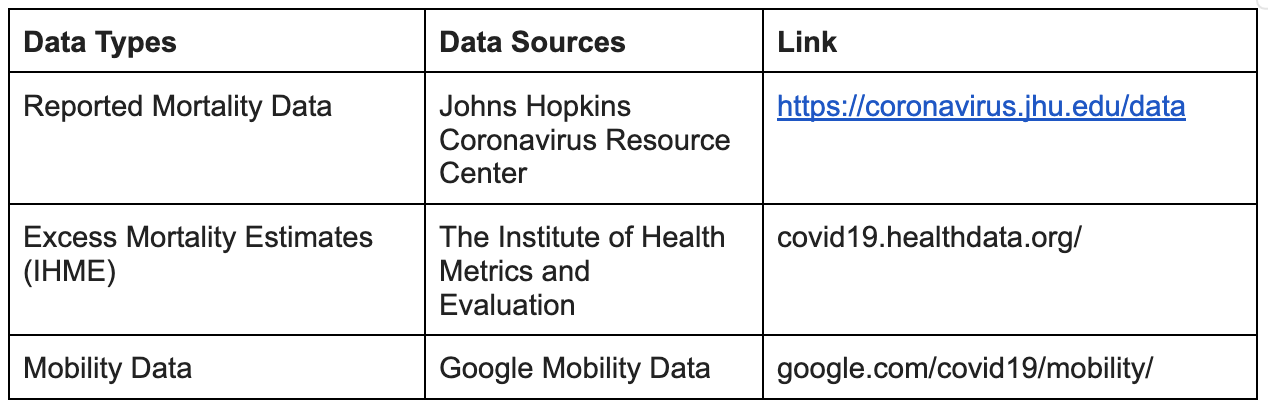

Supplement: S4 Table — (PNG) [file pgph.0002063.s005.png]

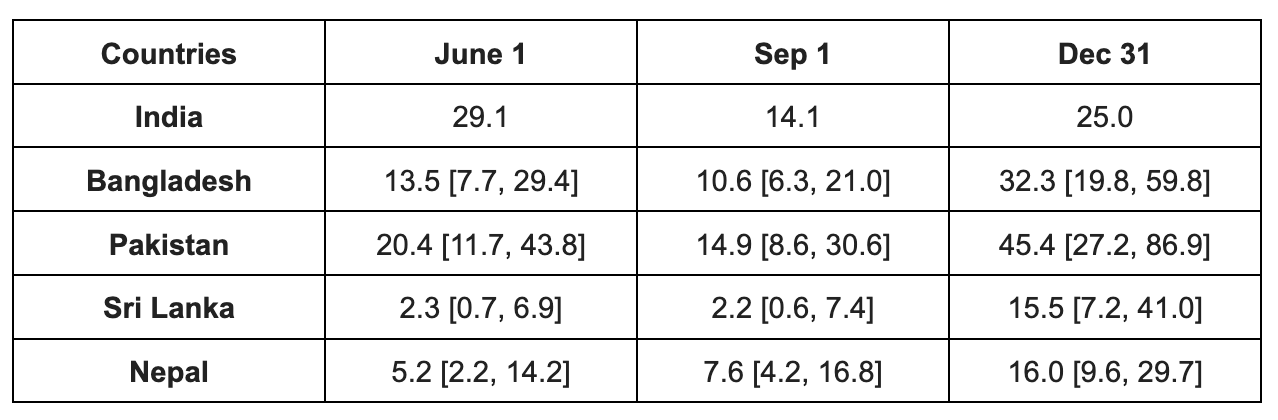

Supplement: S5 Table — For each country, we report the URF for cases on June 1, September 1 and December 31. (PNG) [file pgph.0002063.s006.png]

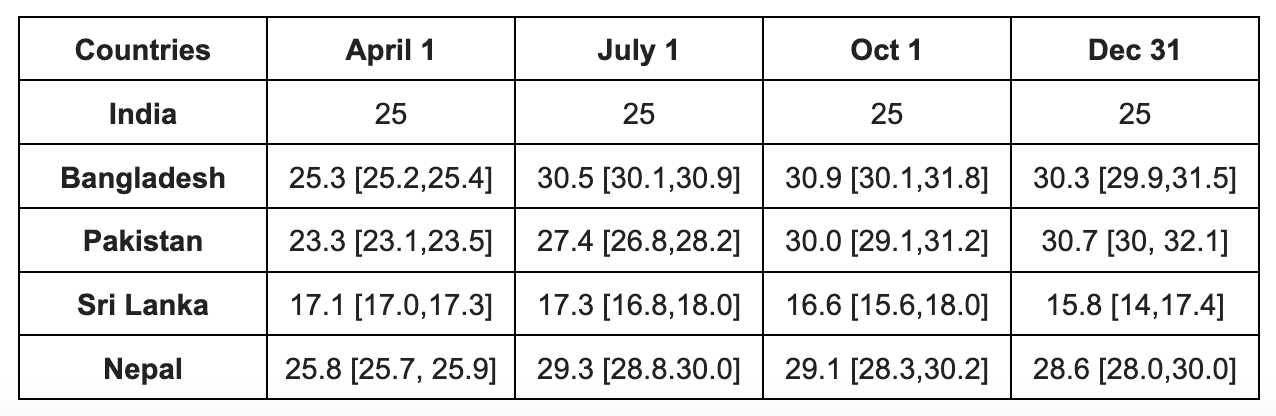

Supplement: S6 Table — For each country, we report the URF for cases on April 1, July 1, Oct 1 and December 31. (PNG) [file pgph.0002063.s007.png]

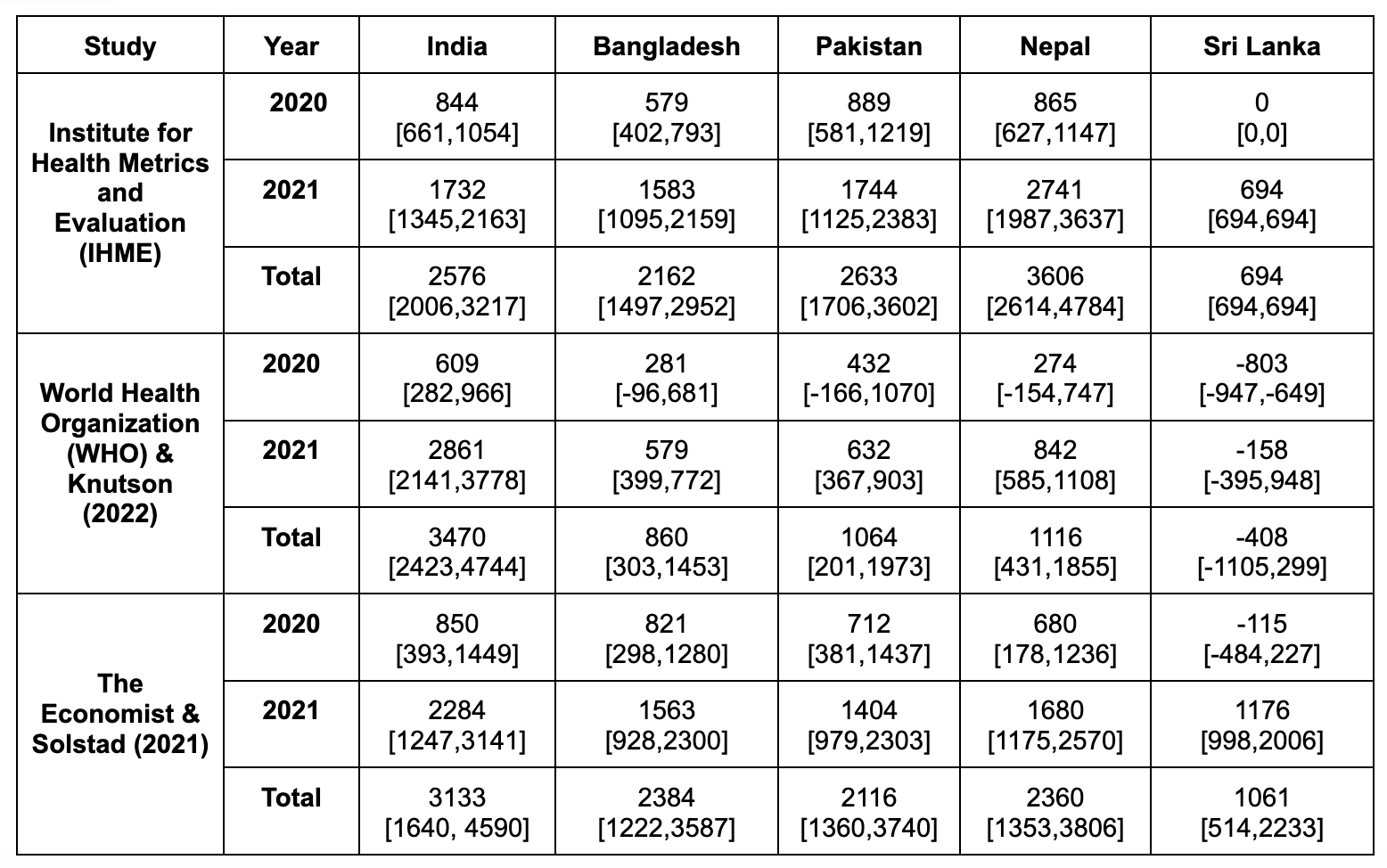

Supplement: S7 Table — (PNG) [file pgph.0002063.s008.png]

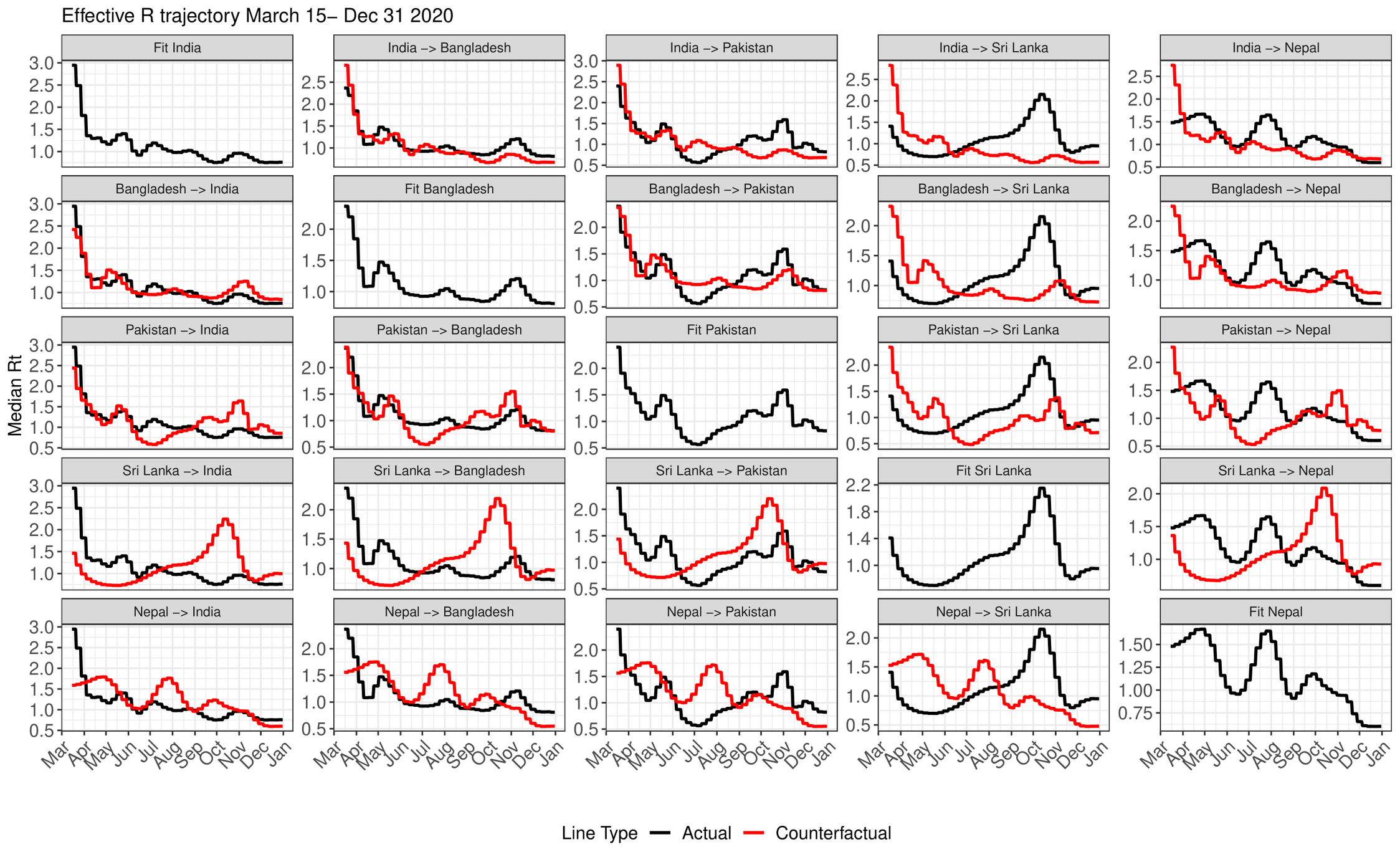

Supplement: S1 Fig — The black lines in the plots denote the actual Rt for the recipient country, while the red lines denote the counterfactual ones. This plot is from the analysis using reported deaths data. (TIF) [file pgph.0002063.s011.tif]

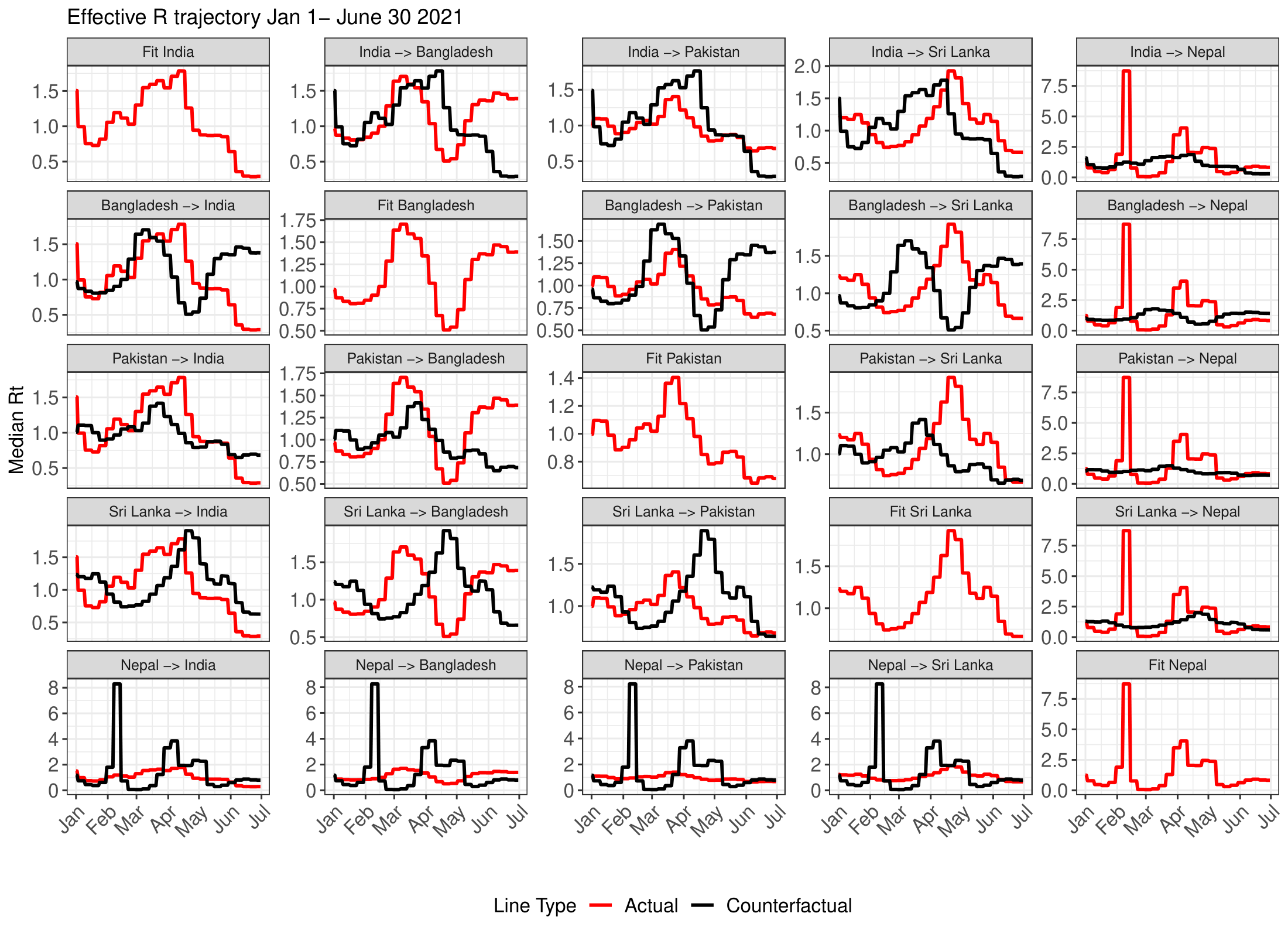

Supplement: S2 Fig — The black lines in the plots denote the actual Rt for the recipient country, while the red lines denote the counterfactual ones. This plot is from the analysis using reported deaths data. (TIF) [file pgph.0002063.s012.tif]

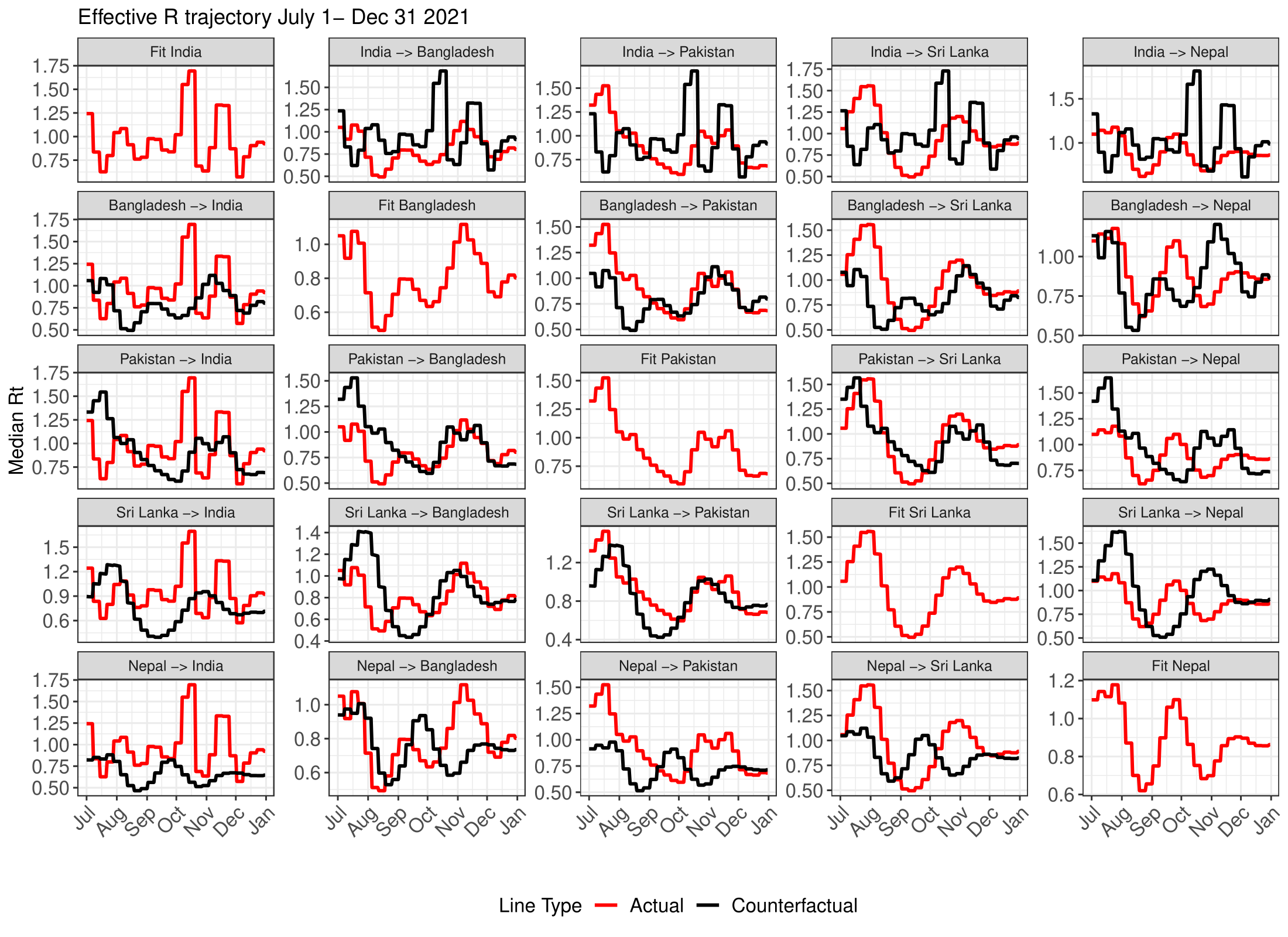

Supplement: S3 Fig — The black lines in the plots denote the actual Rt for the recipient country, while the red lines denote the counterfactual ones. This plot is from the analysis using reported deaths data. (TIF) [file pgph.0002063.s013.tif]

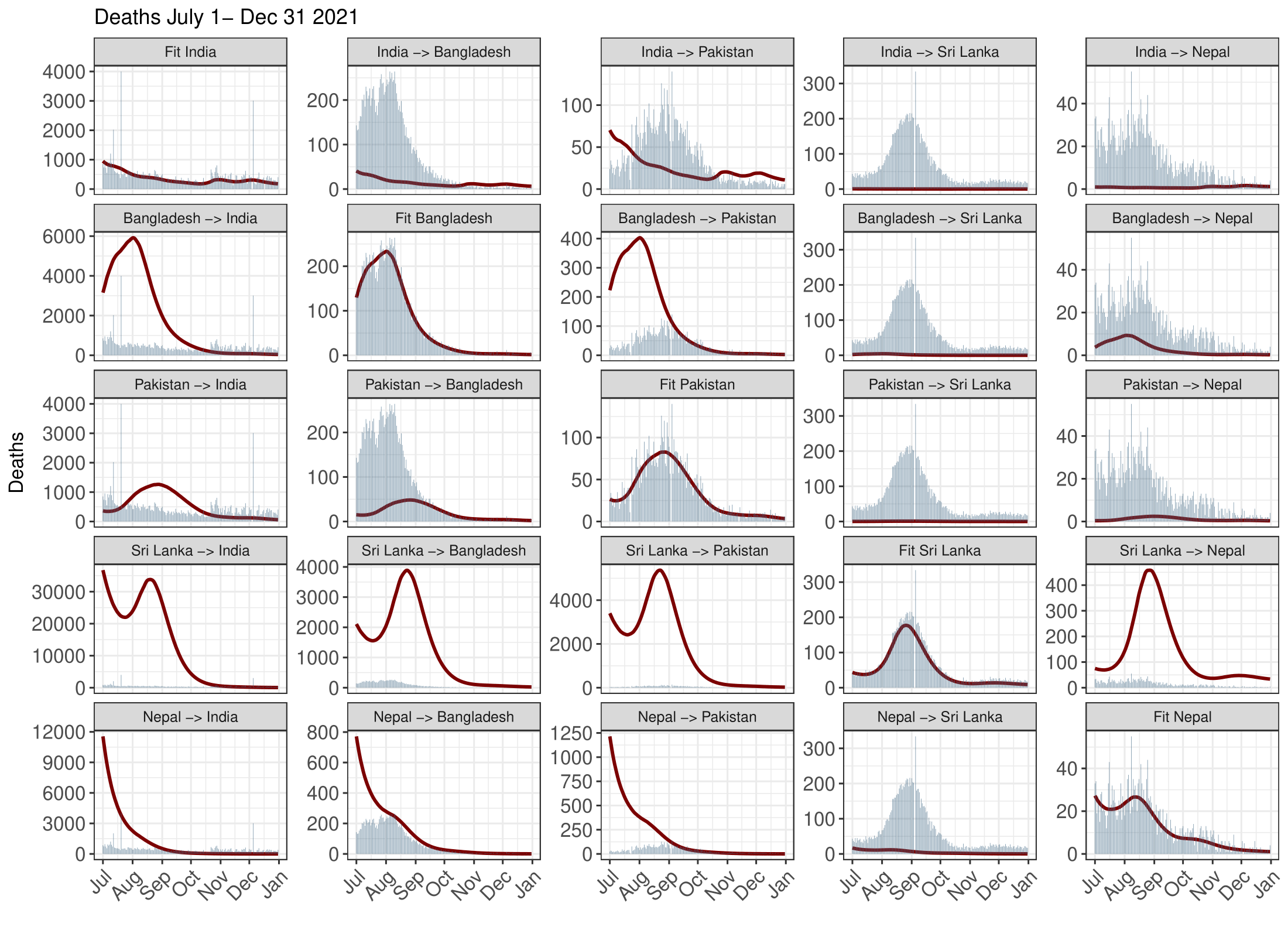

Supplement: S4 Fig — Recipient countries vary along the columns while the donor countries vary along the rows. The blue bars in the plots denote the actual daily death cases for the recipient country, while the red lines denote the counterfactual ones. The time period of analysis is from July 1, 2021 to December 31, 2021. (TIF) [file pgph.0002063.s014.tif]

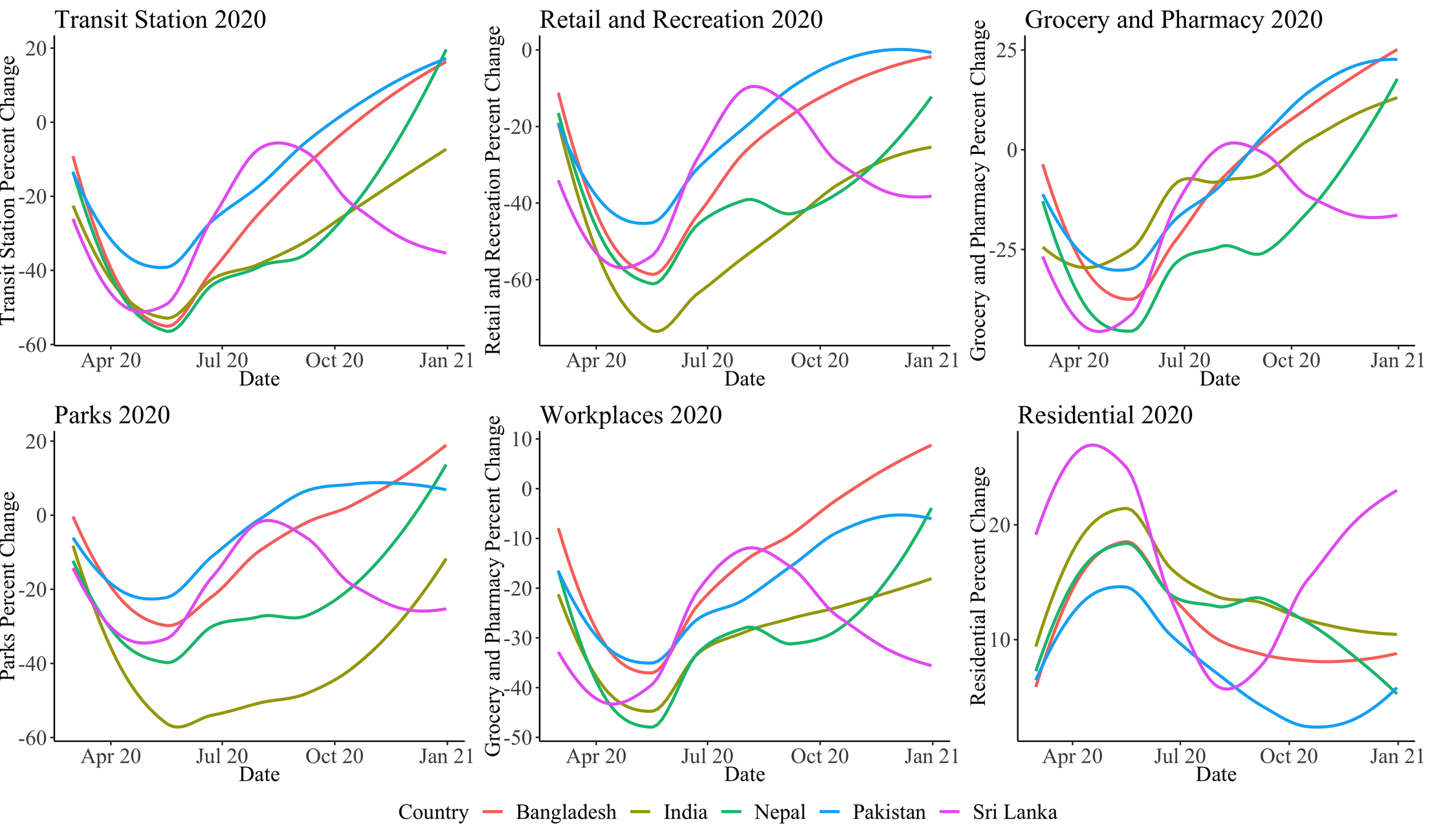

Supplement: S5 Fig — Baseline is the median value for the corresponding day of the week during January 3, 2020–February 6, 2020. Percent changes are reported for Transit Station, Retail and Recreation, Grocery and Pharmacy, Parks, Workplaces, and Residential for the year 2020 (March 15, 2020–December 31,2020). Google Mobility data used for this plot. (TIF) [file pgph.0002063.s015.tif]

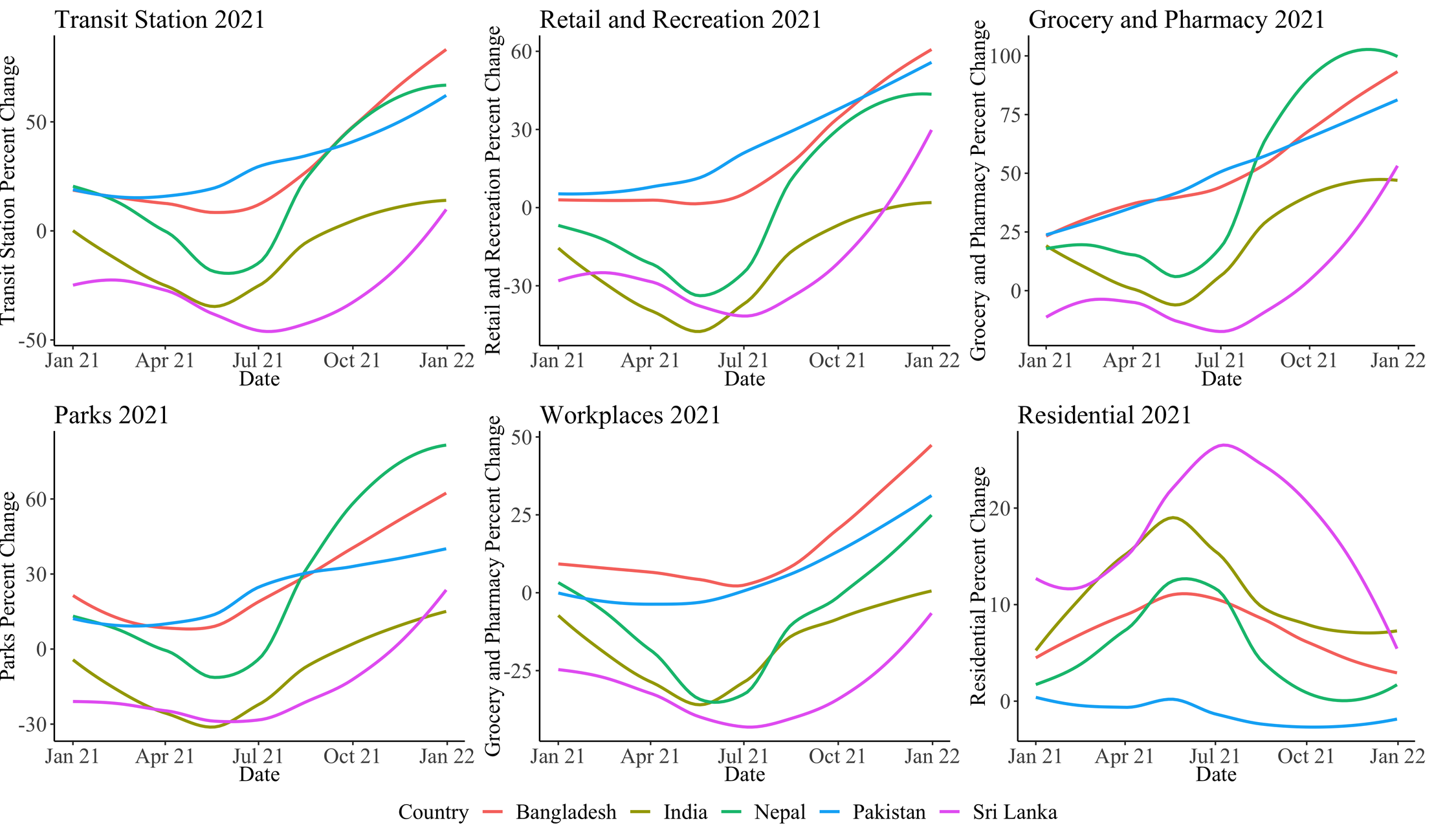

Supplement: S6 Fig — Baseline is the median value for the corresponding day of the week during January 3, 2020–February 6, 2020. Percent changes are reported for Transit Station, Retail and Recreation, Grocery and Pharmacy, Parks, Workplaces, and Residential for the year 2021. Google Mobility data used for this plot. (TIF) [file pgph.0002063.s016.tif]

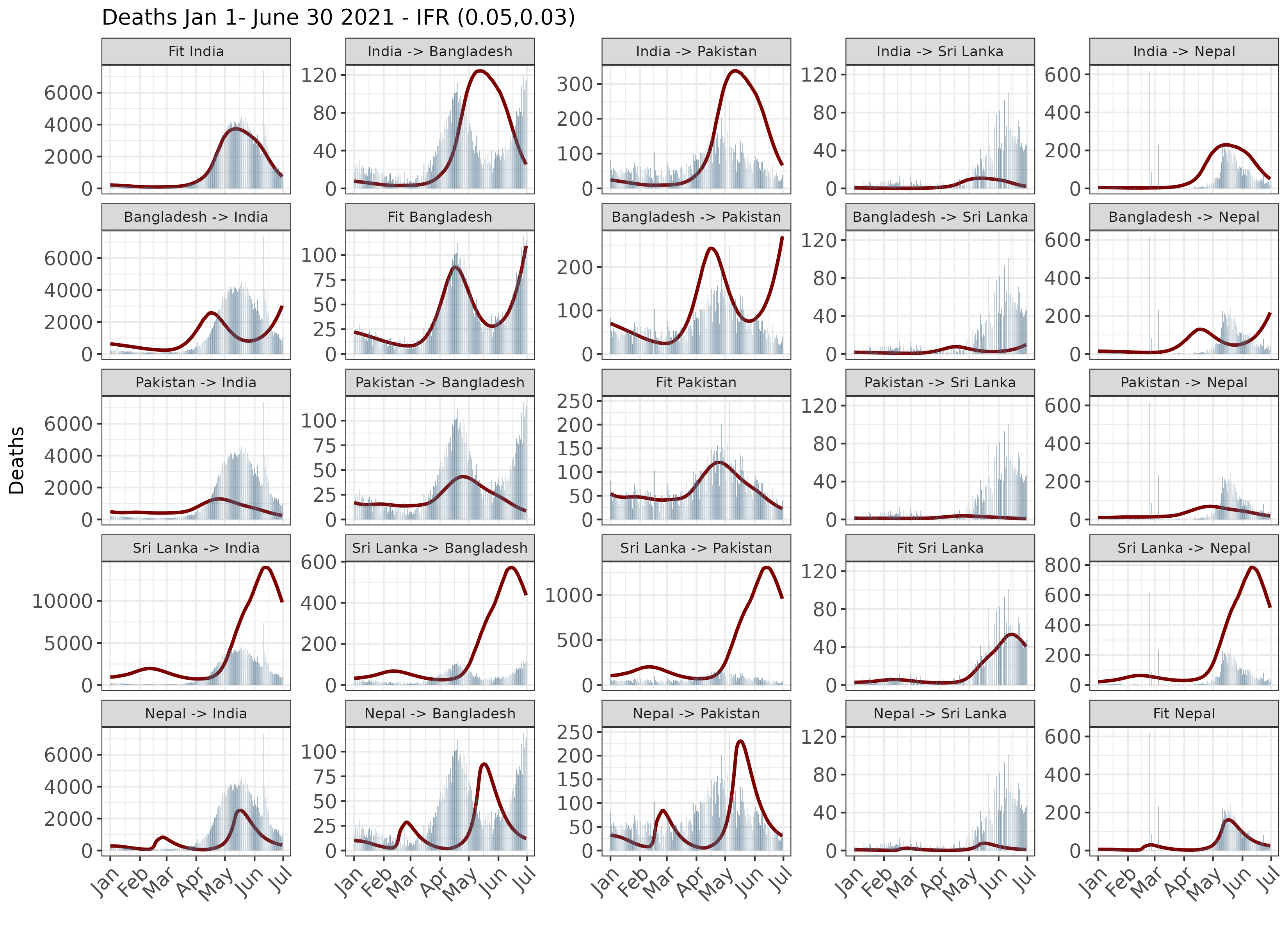

Supplement: S7 Fig — Recipient countries vary along the columns while the donor countries vary along the rows. The blue bars in the plots denote the actual daily death cases for the recipient country, while the red lines denote the counterfactual ones. The time period of analysis is from Jan 1, 2021 to June 30, 2021. (PNG) [file pgph.0002063.s017.png]

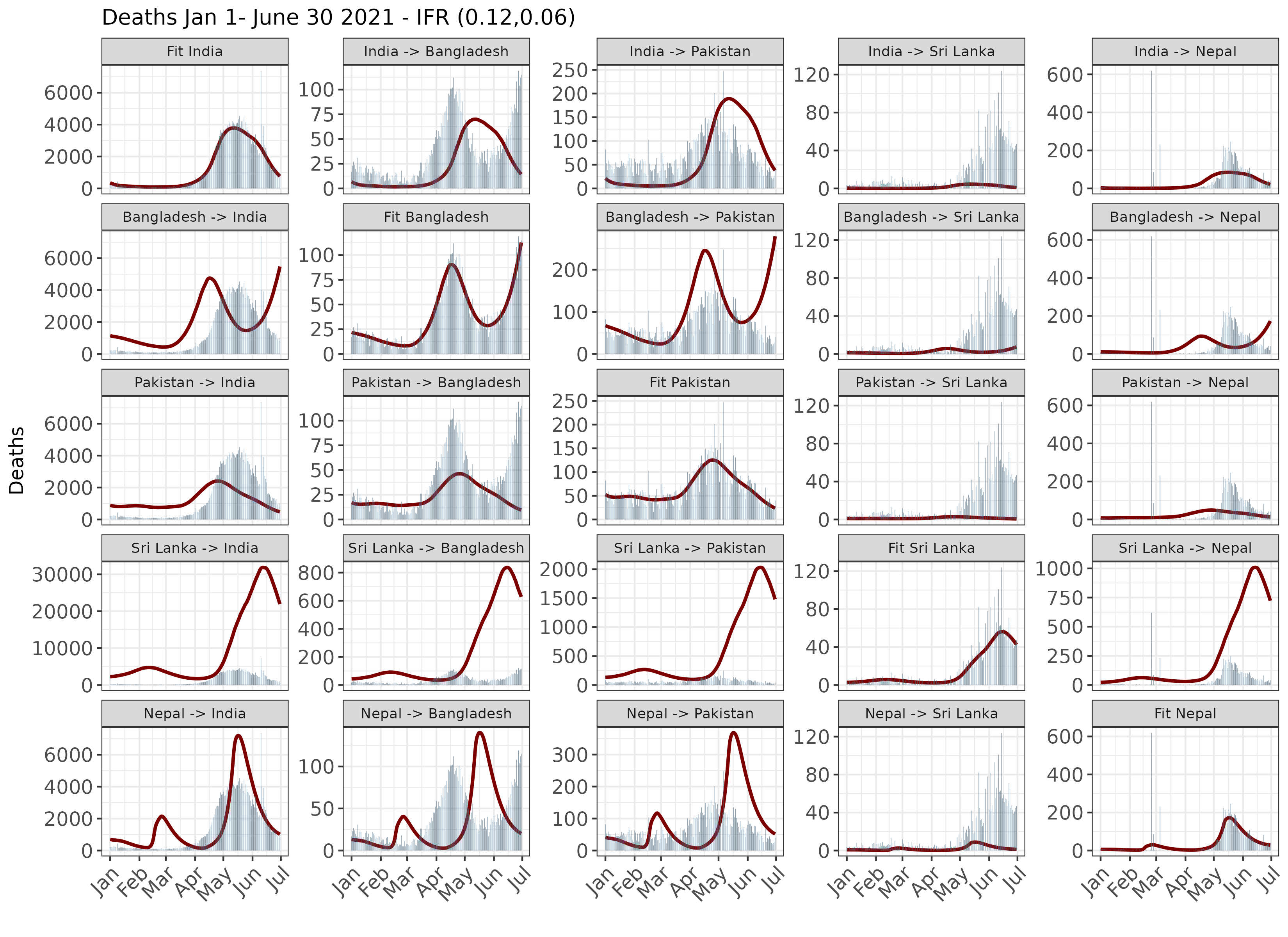

Supplement: S8 Fig — Recipient countries vary along the columns while the donor countries vary along the rows. The blue bars in the plots denote the actual daily death cases for the recipient country, while the red lines denote the counterfactual ones. The time period of analysis is from Jan 1, 2021 to June 30, 2021. (PNG) [file pgph.0002063.s018.png]

# Deaths Jan 1– June 30 2021 Generation Time = 5

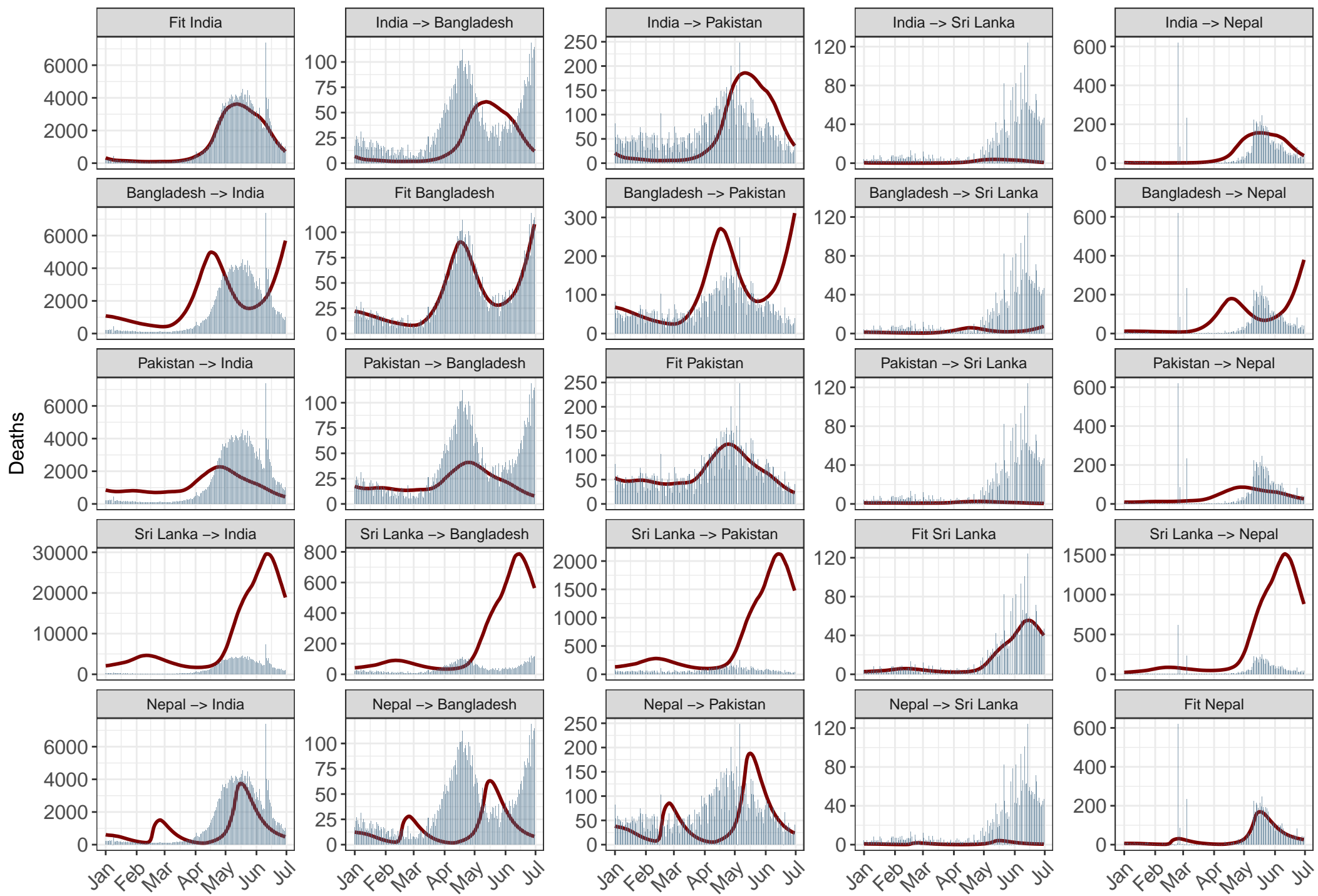

Supplement: S9 Fig — Recipient countries vary along the columns while the donor countries vary along the rows. The blue bars in the plots denote the actual daily death cases for the recipient country, while the red lines denote the counterfactual ones. The time period of analysis is from Jan 1, 2021 to June 30, 2021. (PDF) [file pgph.0002063.s019.pdf]

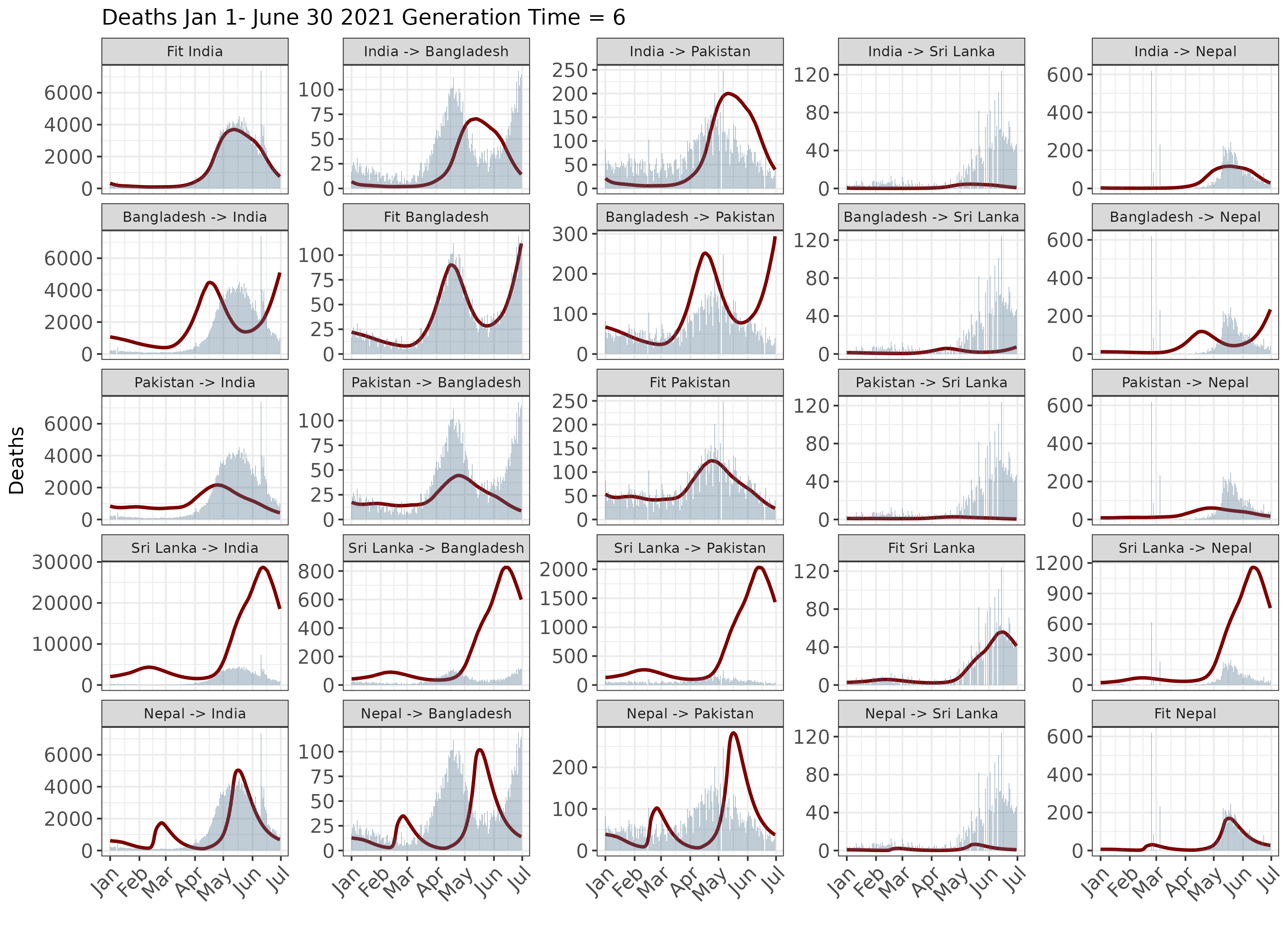

Supplement: S10 Fig — Recipient countries vary along the columns while the donor countries vary along the rows. The blue bars in the plots denote the actual daily death cases for the recipient country, while the red lines denote the counterfactual ones. The time period of analysis is from Jan 1, 2021 to June 30, 2021. (PNG) [file pgph.0002063.s020.png]

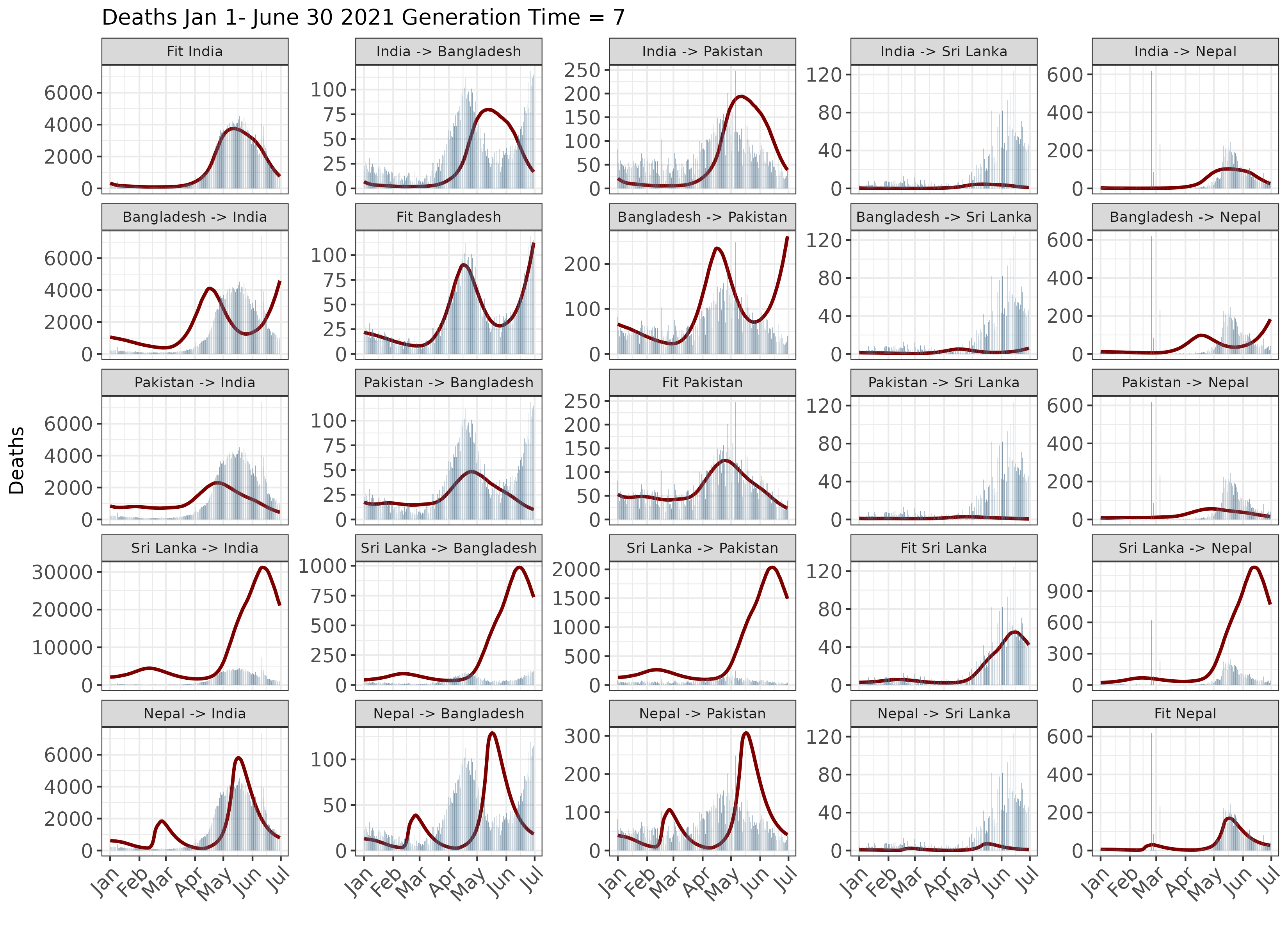

Supplement: S11 Fig — Recipient countries vary along the columns while the donor countries vary along the rows. The blue bars in the plots denote the actual daily death cases for the recipient country, while the red lines denote the counterfactual ones. The time period of analysis is from Jan 1, 2021 to June 30, 2021. (PNG) [file pgph.0002063.s021.png]

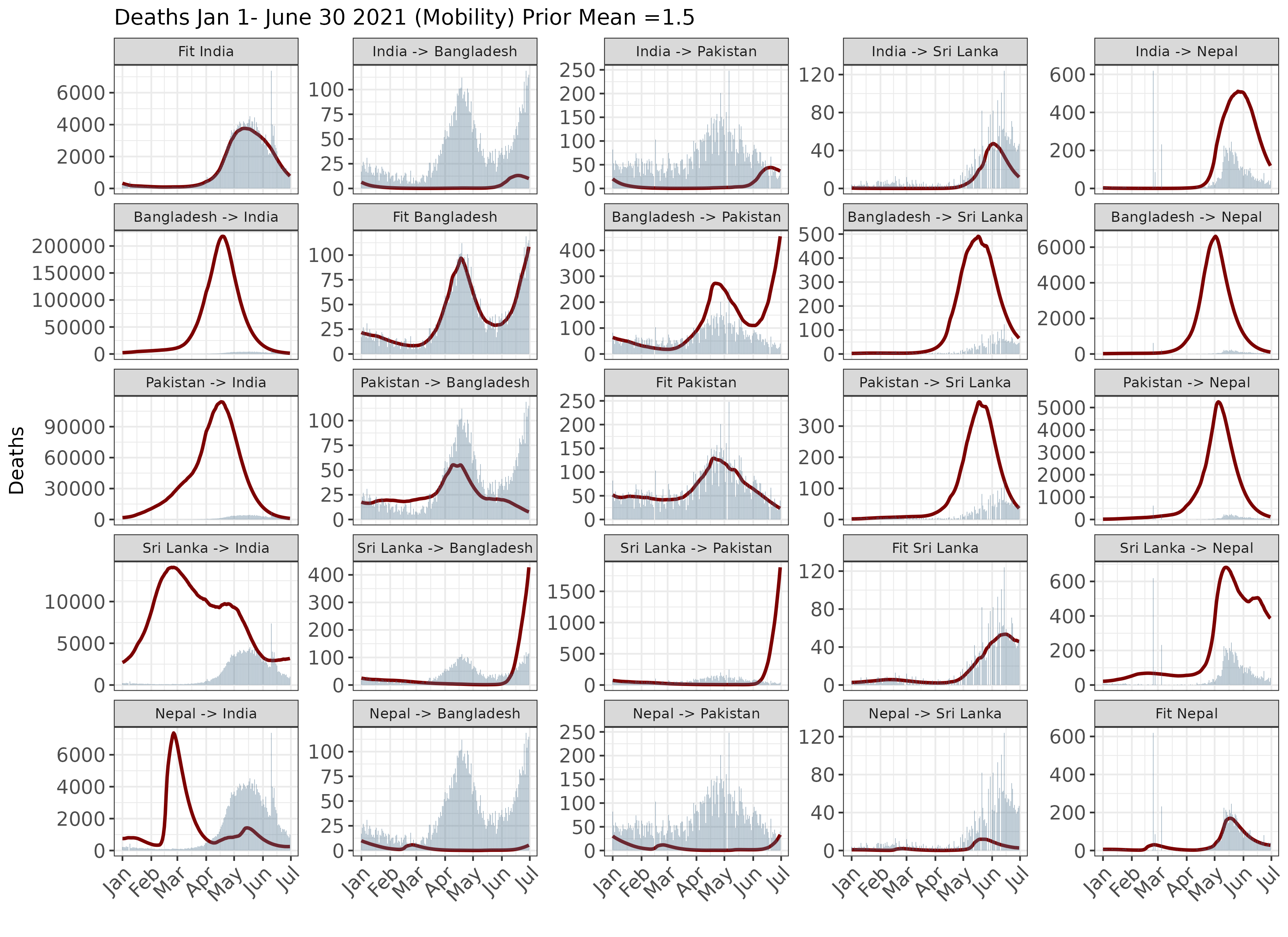

Supplement: S12 Fig — Recipient countries vary along the columns while the donor countries vary along the rows. The blue bars in the plots denote the actual daily death cases for the recipient country, while the red lines denote the counterfactual ones. The time period of analysis is from Jan 1, 2021 to June 30, 2021. (PNG) [file pgph.0002063.s022.png]

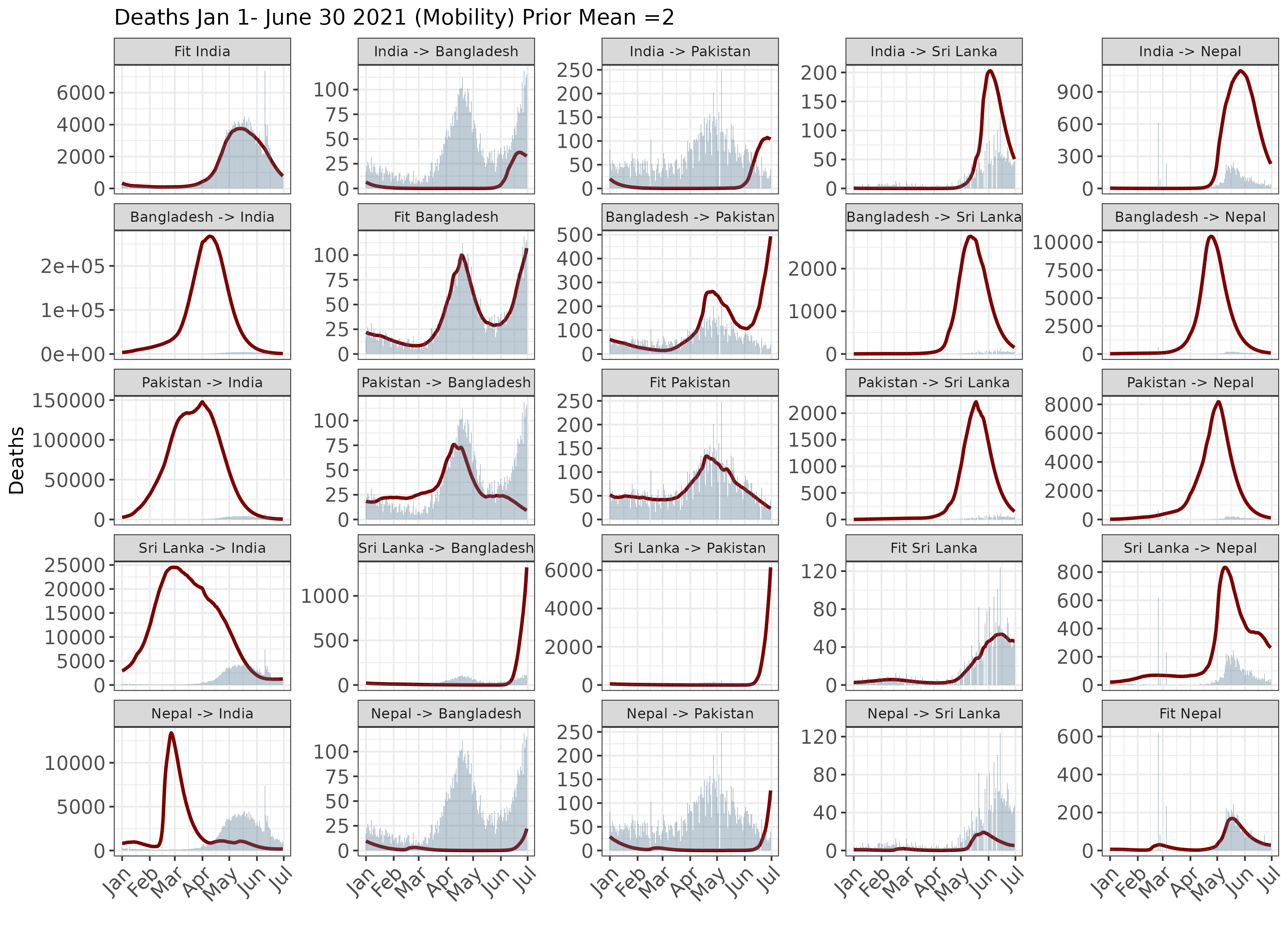

Supplement: S13 Fig — Recipient countries vary along the columns while the donor countries vary along the rows. The blue bars in the plots denote the actual daily death cases for the recipient country, while the red lines denote the counterfactual ones. The time period of analysis is from Jan 1, 2021 to June 30, 2021. (PNG) [file pgph.0002063.s023.png]
