# Supplementary material for: Comparative impact assessment of COVID-19 policy interventions in five South Asian countries using reported and estimated unreported death counts during 2020-2021
Source: PLOS Glob Public Health. 2023 Dec 27;3(12):e0002063. doi: 10.1371/journal.pgph.0002063 (PMC10752546; doi:10.1371/journal.pgph.0002063)
Supplement: S8 Table — (PDF) [file pgph.0002063.s009.pdf]

Table S5 : Timeline of COVID-19 interventions in India, Bangladesh, Nepal, Pakistan, and Sri Lanka from March 15 - December 31, 2020.

India	
March 25, 2020 - April 14, 2020 (21 Days)	Phase 1: Nationwide Lockdown<br><br>All services and factories were shut down.
April 15, 2020 - May 3, 2020 (19 Days)	Phase 2: Nationwide Lockdown<br><br>With a conditional relaxation after April 20 for regions where the spread has been contained.
May 4, 2020 - May 17, 2020 (14 Days)	Phase 3: Nationwide Lockdown<br><br>Extended the lockdown with more relaxations and divided the country into different zones : red, orange and green depending on the number of COVID cases.
May 18, 2020 - May 31, 2020 (14 Days)	Phase 4: Nationwide Lockdown<br><br>Allowed more relaxations to the states.
June 1, 2020 - June 30, 2020 (30 Days)	Unlock 1.0 : Lockdown Measures imposed only in containment zones. Permitted shopping malls, religious places, hotels, and restaurants to reopen from June 8.
July 1, 2020 - July 31, 2020 (31 Days)	Unlock 2.0 : Night curfews were in effect from 10 pm to 5 am in all areas.
August 1, 2020 - August 31, 2020 (31 Days)	Unlock 3.0 : Removed Night Curfews. Educational Institutions remain closed. All inter- and intrastate travel and transportation permitted.
September 1, 2020 - September 30, 2020 (30 Days)	Unlock 4.0 : Metro was allowed from September 7, 2020. Public ceremonies like marriages, religious, sports, political functions were allowed up to a limited number of people.
October 1, 2020 - October 31, 2020 (31 Days)	Unlock 5.0 : More flexibilities were allowed. However lockdown was still imposed in Containment zones.
November 1, 2020 - November 30, 2020 (30 Days)	Unlock 6.0 : Similar to Unlock 5.0. Extended ban for international flight.

Bangladesh	
March 26, 2020 - May 27, 2020	General Lockdown (“General Holiday”) :<br><br>Extended a lot of times. All public and private offices remained closed except emergency services.<br><br>Public transport limited. Army to ensure social distancing.
September 1, 2020	Final set of restrictions on public movement lifted.

Pakistan	
March 24, 2020	Sindh and Balochistan observed lockdown till April 7.<br>Azad Kashmir under lockdown till April 13. Punjab till April 6.
March 25, 2020	Several restrictions imposed in the capital territory of Islamabad.
April 1, 2020 - May 9, 2020	Country wide lockdown imposed.

Nepal	
March 22, 2020	Suspension of international flights and the Visit Nepal 2020 campaign
March 24, 2020 - July 21, 2020	Nationwide Lockdown

Sri Lanka	
March 20, 2020 - May 31, 2020	Nationwide Lockdown

***Source** : Wikipedia*
