# Supplementary material for: Comparative impact assessment of COVID-19 policy interventions in five South Asian countries using reported and estimated unreported death counts during 2020-2021
Source: PLOS Glob Public Health. 2023 Dec 27;3(12):e0002063. doi: 10.1371/journal.pgph.0002063 (PMC10752546; doi:10.1371/journal.pgph.0002063)
Supplement: S9 Table — (PDF) [file pgph.0002063.s010.pdf]

Table S6. Timeline of COVID-19 interventions in India, Bangladesh, Nepal, Pakistan, and Sri Lanka from January 1-December 31, 2021.

Date	***Intervention***
India	
Jan 16, 2021	◆ Nationwide vaccination program begins with initial prioritization of healthcare workers [1]
Jan 29, 2021	◆ Maharashtra extends ongoing partial lockdown until February 28, 2021 [2]
Feb 10, 2021	◆ Maharashtra government extends travel restrictions for travelers coming from Kerala, and a negative test report is mandated. Similar travel restrictions had been instituted in four states (Delhi, Rajasthan, Goa, Gujarat) since November 23, 2020 [3]
Feb 15, 2021	◆ In Maharashtra, in-person classes in colleges and universities reopen with restricted capacity start after >10 months of closure [4]
Feb 18, 2021	◆ Maharashtra sees spike in cases over the prior few days and the biggest single-day spike in cases in the last 70 days lead to restrictions on movement of people in 7 districts of Maharashtra [5]<br>◆ Authorities in some districts impose partial lockdowns [6]
Mar 10, 2021	◆ To curb a COVID-19 surge, Maharashtra districts are asked to ramp up measures like identifying micro-containment zones, increasing contact tracing, and disallowing weddings and other large gatherings [7]
Mar 22, 2021	◆ In Maharashtra, entry into shopping malls without a negative test report is prohibited [8]
Mar 28, 2021	◆ Maharashtra imposes statewide night curfew [9]
Apr 1, 2021	◆ Central government extends vaccine eligibility to adults aged ≥ 45, as announced on March 23 [10]
Apr 2	◆ In Maharashtra, testing capacity and hospital bed capacity are increased [11]
Apr 5, 2021	◆ Maharashtra issues restrictions on malls, restaurants, religious buildings, and cinemas, as announced on April 4 [12]
Apr 8, 2021	◆ Six states at least, including Andhra Pradesh, Chhattisgarh, Haryana, Maharashtra, Odisha and Telangana, report vaccine shortages [13]<br>◆ Madhya Pradesh imposes night curfew in all urban areas, as announced on April 7 [14]
Apr 9, 2021	◆ Maharashtra imposes complete weekend lockdown, in addition to night curfew, statewide [15]<br>◆ In Maharashtra, all shops (except essential ones), malls, and markets are to be shut until April 30 [16]<br>◆ Jammu and Kashmir imposes night curfew in urban regions across eight districts, as announced April 8 [17]<br>◆ Maharashtra suspends vaccine administration in multiple districts [18]<br>◆ Chhattisgarh district of Raipur issues lockdown until April 19, as announced on May 7 [19]

Apr 10, 2021	• Maharashtra enters statewide weekend lockdown, as announced on April 4, [12] which was extended until June 15 [20]<br>• Karnataka imposes night curfew until April 20, as announced on April 9 [21]
Apr 14	• Maharashtra Chief Minister declares a 15-day curfew from April 14 until May 1. All establishments, public places, and services (except essential services) are to remain closed statewide, but inter-district travel is allowed [22]
Apr 16, 2021	• Delhi imposes a weekend curfew, as announced on April 16 [23]<br>• Karnataka imposes restrictions on public gatherings and entertainment activities, as announced on April 16 [21]
Apr 17	• In Maharashtra, school certificate exams are deferred or cancelled [24]
Apr 19, 2021	• Delhi imposes a week-long lockdown until April 26, as announced on April 19, [23] which was extended until June 7 [20]
Apr 20, 2021	• Telangana issues an immediate statewide night curfew, as announced on April 20 [25]
Apr 22, 2021	• Jharkhand imposes lockdown-like restrictions until April 29, as announced on April 20 [26]<br>• Maharashtra goes into a complete lockdown mode after following curfew for a week [27]
Apr 23, 2021	• Puducherry imposes statewide lockdown, as announced on April 21, [28] which was extended until June 7 [20]<br>• Maharashtra places restrictions on inter-state and inter-district travel [29]
Apr 24, 2021	• Andhra Pradesh issues a statewide night curfew, as announced on April 23 [30]<br>• Uttar Pradesh issues a statewide weekend lockdown, as announced on April 20, [31] which was extended until June 1 [20]
Apr 27, 2021	• Assam imposes statewide night curfew through May 7, as announced on April 27 [32]<br>• Himachal Pradesh announces night curfew across 1/3 of all districts and issues weekend lockdown, as announced on April 25 [33]<br>• Karnataka imposes a two-week statewide close down, as announced on April 26 [34]
Apr 29, 2021	• Maharashtra extends the lockdown, with exemption of essential services until May 15 [35]
Apr 30, 2021	• Haryana issues a weekend curfew across nine districts, as announced on April 30 [32]<br>• West Bengal issues restrictions including a ban on gatherings as well as shutdown of cinemas, gyms, malls and restaurants, as announced on April 30 [32], which were extended until June 15 [20]<br>• Nagaland enters statewide lockdown until May 14, as announced on April 27, [36] which was extended until June 11 [37]
May 1, 2021	• Central government extends vaccine eligibility to adults aged ≥ 18 [38]

May 3, 2021	◆ Haryana imposes a statewide lockdown through May 10, as announced on May 3, [32] which was extended until June 7 [20]<br>◆ Select districts in Mizoram, including Aizawl, enter an eight-day lockdown, as announced on May 2, [39] which was extended until June 6 [40]<br>◆ Punjab issues a weekend lockdown and a night curfew through May 15, as announced on May 3, [41] which was extended until June 10 [20]
May 5, 2021	◆ Bihar issues statewide lockdown until May 15, as announced on May 4, [32] which was extended until June 8 [20]<br>◆ Odisha enters a 14-day statewide lockdown until May 19, as announced on May 2, [42] which was extended until June 17 [43]<br>◆ Andhra Pradesh enters a statewide 14-day partial curfew, as announced on May 3, [44] which was extended until June 10 [20]
May 7, 2021	◆ Gujarat issues night curfew on twenty-nine cities, and travel restrictions as well as social distancing measures through April 30, as announced May 7, [45] which was extended until June 4 [20]
May 8, 2021	◆ Kerala enters a statewide lockdown until May 16, as announced on May 6, [32] which was extended until June 9 [20]
May 9, 2021	◆ Goa imposes a statewide curfew until May 23, as announced on May 7 [32]
May 10, 2021	◆ Karnataka imposes a statewide lockdown until May 24, as announced on May 7, [32] which was extended until June 7 [20]<br>◆ Rajasthan enters a statewide lockdown until May 24, as announced on May 6, [32] which was extended to June 2 [20]<br>◆ Tamil Nadu issues a statewide lockdown until May 24, as announced on May 8, [32] which was extended until June 7 [20]
May 12, 2021	◆ Telangana imposes statewide lockdown, as announced on May 11, [46] which was extended until June 9 [20]
May 13	◆ Maharashtra lockdown is extended until June 1, with stricter restrictions implemented at the district level wherever cases are on the rise [47, p. 1]<br>◆ Maharashtra plans to create a separate database of mucormycosis (‘black fungus’) cases to assess its frequency during the second wave of COVID-19 [48]
May 23, 2021	◆ Rajasthan extends lockdown until June 8, as announced on May 23 [49]
May 27, 2021	◆ Maharashtra districts with poor vaccination rates are prioritized to get more vaccine doses [50, p. 19]<br>◆ Maharashtra state government announces a pediatric task force in preparation for the next wave [51]

May 31, 2021	◆ Maharashtra lockdown extended until June 15 but with relaxation of restrictions in districts showing reduced test positive rates and occupancy of hospital beds with oxygen support [52, p. 15]
Jun 5, 2021	◆ Himachal Pradesh extends night curfew statewide until June 14, as announced on June 5 [53]
Jun 7	◆ Across Maharashtra, 5-level unlocking plan starts; levels of relaxation of restrictions are defined based on an area's test positivity rate and availability of hospital beds with oxygen [54]
Jun 8, 2021	◆ Madhya Pradesh extends full lockdown through June 15, as announced on June 8, whilst allowing markets in Ujjain to remain open with a curfew and requiring shop employees to be vaccinated. Weekend curfew remains in effect for Bhopal [55]<br>◆ Rajasthan relaxes lockdown restrictions from June 8, as announced on June 8 [56]
Jun 9, 2021	◆ Bihar lifts full lockdown from June 9, as announced on June 9, with certain restrictions remaining [57]
Jun 27, 2021	◆ Rajasthan further eases restrictions from June 28, whilst mandating vaccination of shop employees and the allowance of those vulnerable to work remotely (e.g., those whom are pregnant or have comorbidities, etc.), as announced on June 27 [58]
Jun 28, 2021	◆ Maharashtra increases restrictions after cases from Delta+ variants rise; 5-level unlocking plan is reduced to a 3-level unlock plan, with the first 2 levels with maximum relaxations removed [59]
Jul 1, 2021	◆ Telangana extends restrictions through July 31, as announced on July 1 [60]
July 15	◆ In Maharashtra, schools for classes (grades) 8 to 12 to reopen in non-covid zones with low COVID-19 transmission [61]
July 25, 2021	◆ Jammu and Kashmir relaxes restrictions by lifting statewide weekend curfew, while night curfew remains in place, as announced on July 25 [62]
July 26	◆ Maharashtra state government relaxes some rules this week for districts with a low test positivity rate [63]
July 27, 2021	◆ Chhattisgarh extends lockdown through August 6, as announced on July 27 [64]
July 30, 2021	◆ Jharkhand extends restrictions and re-opens schools for grades 9-12, as announced July 31[65]<br>◆ Nagaland extends lockdown measures through August 31, as announced on July 30, with Kohima and 2 other cities under full lockdown [66]
July 31, 2021	◆ Karnataka extends restrictions through August 16, as announced on July 31, and announces target of having 72% of teachers vaccinated [67]<br>◆ Punjab plans to re-open school for all classes on August 2, as announced on July 31 [68]
August 1, 2021	◆ Jammu and Kashmir extend night curfews and schools to remained closed until further notice, as announced on August 1 [69]<br>◆ Himachal Pradesh extends night curfew in containment zones through August 31, as announced on August 1 [70]

	◆ Odisha relaxes restrictions on shopping malls and cinemas from August 1, with the exception of Bhubaneswar and two other cities were cases are high, as announced on August 1 [71]
August 2, 2021	◆ Maharashtra relaxes restrictions in all but 11 districts, where cases are high. Weekday and weekend curfew for shops and restaurants remain in effect in 11 districts, as announced on August 2 [72]
August 4, 2021	◆ Bihar relaxes restrictions by re-opening shops, malls, and cinemas, as well as through re-opening schools for classes 9-10 on August 7 and for classes 1-8 on August 16, as announced on August 4 [73]<br>◆ Kerala extends restrictions of allowing shops, banks, and markets to be open except Sunday from August 5 and mandating employees of shops and such establishment to be vaccinated, as announced on August 4. Kerala also lifts lockdown for Independence Day on August 15 [74]
August 6, 2021	◆ Karnataka tightens night curfew and plans to open schools on August 23 for classes 9-12, as announced on August 6 [75]
August 8, 2021	◆ Jammu and Kashmir temporarily lift restriction on gathering of 25+ people on August 15 for Independence Day, as announced on August 8 [76]<br>◆ Odisha relaxes weekend curfew in three cities for Independence Day on August 15, as announced on August 8 [77]<br>◆ Haryana extends lockdown restrictions to August 23, while lifting curfew restrictions, as announced on August 8 [78]
August 9, 2021	◆ Maharashtra relaxes restrictions for Pune and Pimpri Chinchwad allowing a later close time for shops and restaurants and malls to be open for the fully vaccinated, as announced on August 9 [79]<br>◆ Assam implements a partial curfew of night curfew from August 10, as announced on August 9 [80]
August 11, 2021	◆ Uttar Pradesh relaxes weekend lockdown to Sunday only (in addition to weekday curfew) from August 14, as announced on August 11, and plans to have schools open August 16 for grades 9-12 and September 1 for grades 6-8 [81]<br>◆ Himachal Pradesh states that all schools will remain closed until August 22, as announced on August 11 [82]
August 12, 2021	◆ West Bengal extends restrictions to August 30 while reducing the time window of the night curfews, as announced August on 12 [83]
August 15, 2021	◆ Puducherry extends restrictions through August 31, including night curfew, as announced on August 15 [84]
August 21, 2021	◆ Karnataka advises IT companies in Bengaluru to extend remote work through December 2022, as announced on August 21 [85]<br>◆ Andhra Pradesh extends restriction (i.e., night curfew) through September 4, as announced on August 21 [86]

	◆ Tamil Nadu extends lockdown until September 6 and eases curfew on shops, as well as plans to re-open schools for classes 9-12 on September 1 and classes 1-8 on September 15, as announced on August 21 [87]
August 23, 2021	◆ Delhi relaxes restrictions by easing curfew on markets, shops, malls, and restaurants on August 23, as announced on August 21 [88]<br>◆ Goa extends night curfew until August 30, as announced on August 23 [89]
August 24, 2021	◆ Telangana plans to reopen schools on September 1, as announced on August 24 [90]<br>◆ Gujarat relaxes night curfew in 8 cities on August 30 for Janmashtami and Ganesh Festivals, as announced on August 24 [91]
August 27, 2021	◆ Kerala extends restriction of weekend lockdown, as announced on August 27 [92]
Sep 1, 2021	◆ Delhi reopens secondary school (grades 9-12) [93]<br>◆ Tamil Nadu reopens secondary school (grades 9-12) [93]
Oct 25, 2021	◆ Karnataka reopens school grades 1-5 [93]
Nov 1, 2021	◆ Delhi reopens remaining schools [93]<br>◆ Kerala begins reopening school grades intermittently [93]<br>◆ Tamil Nadu reopens remaining schools [93]
Nov 16, 2021	◆ West Bengal reopens school grades 9-12 [93]
Nov 26, 2021	◆ India issues further screening for travel to and from South Africa, Botswana, Zimbabwe, United Kingdom, Brazil, Bangladesh, China, Mauritius, New Zealand, Singapore, Hong Kong and Israel [94]
Nov 27, 2021	◆ Maharashtra lifts remaining lockdown restrictions [95]
Dec 20, 2021	◆ Chandigarh closes schools, amidst Omicron COVID-19 variant [96]
Dec 28, 2021	◆ Delhi closes schools and colleges, amidst Omicron variant [97]
Bangladesh	
Jan 27, 2021	◆ Vaccination program begins in Bangladesh[98]
Mar 29, 2021	◆ Bangladesh announces guidelines including 50% reduced office, restaurant and transportation occupancy, as well as restricting political, religious, and other gatherings. [99] The statement also directed a ban of transportation services in areas of high-risk.
Apr 1, 2021	◆ Tourism minister announces closure of tourist and entertainment locations [100]<br>◆ Civil aviation regulator announces ban on travelers into Bangladesh from all European countries (except the UK) and twelve other countries to start on April 3, as announced April 1 [101]
Apr 3, 2021	◆ Bangladesh imposes full country-wide lockdown on April 5, as announced on April 3, [102] encompassing suspension of domestic travel and closure of shopping malls

Apr 9, 2021	◆	Bangladesh announces strict, week-long countrywide lockdown to begin April 14, as announced on April 9. [103] During this period, private and government offices, industries, factories, such as the garment factories remained closed. Transportation is also suspended, except for emergency services.
Apr 11, 2021	◆	Bangladesh announces ban on international travel to and from Bangladesh beginning April 14, as announced on April 11 [101]
Apr 25, 2021	◆	Bangladesh closes border with India from April 26, as announced April 25 [104]
May 3, 2021	◆	Bangladesh allows intra-district bus travel from May 6, as announced May 3, [105] while long-distance bus operations, as well as all train, inland water launches, and flight travel, remain suspended
May 23, 2021	◆	Bangladesh allows bus, train and inland water launches to resume at 50% capacity with other ongoing restrictions to remain in place [106]
Jul 5, 2021	◆	Vaccine eligibility extended to those 35 years and older [107]
Jun 21, 2021	◆	Seven districts within Dhaka issued strict lockdown from June 22, as announced June 21, [108] in response to rising transmission rates
Jun 27, 2021	◆	Bangladesh imposes countrywide lockdown from July 1, [109] which was extended from original announcement of starting June 28
Jul 15, 2021	◆	Bangladesh eases lockdown restrictions for Eid-al-Adha celebrations through July 23, as announced July 13 [110]
Jul 19, 2021	◆	Vaccine eligibility extended to those 30 years and older [111]
Jul 29, 2021	◆	Vaccine eligibility extended to those 25 years and older [112, p. 19]
Aug 1, 2021	◆	Bangladesh eases lockdown restrictions by allowing garment factories to reopen [113]
Aug 11, 2021	◆	Bangladesh lifts countrywide lockdown, allowing offices, shops, and public transportation services to resume [114]
Sep 13, 2021	◆	Bangladesh allows reopening of schools nationwide [115]
Sep 16, 2021	◆	Vaccination effort begins for university students, faculty, and other employees [116]
Oct 20, 2021	◆	Vaccine eligibility extended to those 18 years and older [112, p. 19]
Nov 1, 2021	◆	Vaccine eligibility extended to those 12 years and older [117]
Nov 27, 2021	◆	Bangladesh bans travel to and from South Africa, amidst Omicron COVID-19 variant [118]
Nepal		
Jan 27, 2021	◆	Vaccination program begins in Nepal [119]
Apr 19, 2021	◆	Central government closes school, as originally indicated on April 10 [120]
Apr 26, 2021	◆	Nepal issues country-wide lockdown from April 29, as announced April 26 [121]

Apr 30, 2021	◆ Kathmandu-based oxygen manufacturers suspend oxygen supply to industries, in order to serve hospital demand [122]
May 1, 2021	◆ Nepal closes 22 entry points with India border, leaving 13 entry points in operation [123]
May 3, 2021	◆ Nepal suspends international flights with exception of flights between Kathmandu and Delhi, India [124]
May 17, 2021	◆ Nepal-India border entry points still open report having insufficient COVID-19 tests and are only testing symptomatic individuals, amidst influx in Nepalese nationals fleeing India [125]
Jun 21, 2021	◆ Nepal in-part relaxes lockdown provisions, allowing private vehicles to return to the road, while extending lockdown to June 28 [126]
Jun 23, 2021	◆ Nepal announces that international and domestic flights may resume on June 24 and July 1, respectively, with 50% capacity and to limited destinations [127]
Aug 20, 2021	◆ Kathmandu students 18 years and older receive vaccine [128]
Sep 1, 2021	◆ Nepal lifts lockdown in Kathmandu Valley [129]
Sep 19, 2021	◆ Select schools in Kathmandu Valley reopen [130]
Nov 14, 2021	◆ Vaccine eligibility extended to those 12 years and older [131]
Dec 3, 2021	◆ Nepal bans travel to and from South Africa, Botswana, Zimbabwe, Namibia, Lesotho, Eswatini, Mozambique, Malawi and Hong Kong, and central government recommends against non-essential travel, amidst Omicron COVID-19 variant [132]
Pakistan	
Jan 18, 2021	◆ Secondary school (grades 9-12) resume in-person classes nationwide [133]
Feb 1, 2021	◆ Primary school and universities reopen nationwide [133]
Feb 3, 2021	◆ Vaccination program begins in Pakistan [134]
Apr 23, 2021	◆ NCC meeting for implementation of nonpharmaceutical interventions [135]
Mar 15, 2021	◆ Schools and universities close in 7 cities in Punjab, as announced March 10 [136]
Mar 18, 2021	◆ Three districts of Punjab enter “smart lockdown”, described as a short-term, neighborhood approach [137]
Mar 23, 2021	◆ Ten cities issued full lockdown through April 11, including Bahawalpur, Faisalabad, Hyderabad, Islamabad, Lahore, Multan, Muzaffarabad, Peshawar, Rawalpindi, and Swat [138]
May 1, 2021	◆ Pakistan suspends 80% of inbound international flights from May 5, as announced May 1 [139]
May 5, 2021	◆ Lowest rate of vaccination in South Asia reported for Pakistan, as 2 million at least partially vaccinated among 220 million, with considerable vaccine hesitancy [140]
May 8, 2021	◆ Pakistan issues countrywide lockdown from May 8 in concurrence with Eid holidays [141]

May 24, 2021	◆	Pakistan partially eases restrictions, allowing outdoor restaurants and the tourism sector to reopen, as well as schools to resume in districts below a threshold positivity rate, as announced May 19 [142]
May 26, 2021	◆	Vaccine eligibility extended to those 19 years and older [143]
Jun 1, 2021	◆	Outdoor marriage ceremonies with maximum of 150 persons and elective surgeries resume nationwide, as announced May 19 [142]
Jun 16, 2021	◆	Provinces Punjab and Sindh issue measure of banning cellphone service for those unvaccinated, additionally pay for government employees is suspended for those unvaccinated [144]
Jul 24, 2021	◆	Sindh province closes schools, which was extended to August 30 [145]
Jul 29, 2021	◆	Central government requires vaccination card for entry into public offices, schools, shopping centers, restaurants, travel services, and domestic flights [146]
Jul 31, 2021	◆	City of Karachi enters partial lockdown with closing of retail services, such as indoor and outdoor dining, as announced July 30 [146]
Nov 27, 2021	◆	Pakistan bans travel to and from South Africa, Lesotho, Eswatini, Mozambique, Botswana, Namibia and Hong Kong, amidst Omicron COVID-19 variant [147]
Dec 6, 2021	◆	Pakistan further bans travel to and from Croatia, Hungary, Netherlands, Ukraine, Ireland, Slovenia, Vietnam, Poland and Zimbabwe, amidst Omicron variant [148]
Sri Lanka		
Jan 28, 2021	◆	Sri Lanka begins vaccination program [149]
Mar 29, 2021	◆	Sri Lanka reopens schools, as announced March 25 [150]
Apr 23, 2021	◆	Sri Lanka issues Risk Alert Level 3 restrictions through May 31, including only 2 persons per household permitted to be outside, maximum of 150 persons at weddings, universities closed, 50% capacity for grade schools, restaurants, shopping malls, supermarkets, and 25% capacity at cinemas [151]
Apr 27, 2021	◆	Sri Lanka closes all schools [152]
May 6, 2021	◆	Sri Lanka closes border with India by suspending incoming flights [153]
May 10, 2021	◆	Sri Lanka restricts inter-province travel [154]
May 21, 2021	◆	Sri Lanka suspends domestic trains and bus services, except for use by essential services [155]
May 24, 2021	◆	Sri Lanka issues full lockdown from May 25, as announced May 24 [156]
Jun 20, 2021	◆	Sri Lanka issues new guidelines effective June 21, including two individuals permitted to leave household at given time and public transport allowed with 50% capacity, except in Western province where only essential services may utilize public transport [157]
Sep 3, 2021	◆	Vaccine eligibility extended to those 20 and older [158]
Oct 15, 2021	◆	Vaccine eligibility extended to those 18 and older [159]

Oct 21, 2021	◆ Primary schools reopen in-person with secondary with levels to reopen October 25 [160]
Nov 27, 2021	◆ Sri Lanka bans travel to and from South Africa, Botswana, Zimbabwe, Namibia, Lesotho and Eswatini [161]
Dec 10, 2021	◆ Sri Lanka lifts ban on travel from six countries [162]

[a] Entries for India are revised from Table S5 in Salvatore et al., 2021 (Salvatore, M., Bhattacharyya, R., Purkayastha, S., Zimmermann, L., …, & Mukherjee, B. (2021). *Resurgence of SARS-CoV-2 in India: Potential role of the B.1.617.2 (Delta) variant and delayed interventions*. medRxiv. https://doi.org/10.1101/2021.06.23.21259405).

[1] "Coronavirus | World's largest vaccination programme begins in India on January 16," *The Hindu*, New Delhi, Jan. 15, 2021. Accessed: Jun. 07, 2021. [Online]. Available: https://www.thehindu.com/news/national/coronavirus-worlds-largest-vaccination-programme-begins-in-india-on-january-16/article33582069.ece

[2] F. Malik, "Covid-19: Maharashtra govt extends lockdown restrictions till 28 February," *Hindustan Times*, Jan. 29, 2021. https://www.hindustantimes.com/cities/mumbai-news/maharashtra-extends-covid-19-restrictions-by-a-month-101611904456153.html (accessed Aug. 27, 2021).

[3] F. Malik, "Maharashtra extends travel restrictions for Kerala after rise in Covid cases," *Hindustan Times*, Feb. 10, 2021. https://www.hindustantimes.com/cities/mumbai-news/maharashtra-extends-travel-restrictions-for-kerala-after-rise-in-covid-cases-101612980127471.html (accessed Aug. 27, 2021).

[4] F. Malik, "Maharashtra to reopen colleges from February 15," *Hindustan Times*, Feb. 04, 2021. https://www.hindustantimes.com/cities/mumbai-news/maharashtra-to-reopen-colleges-from-february-15-101612379336390.html (accessed Aug. 27, 2021).

[5] S. P. Gangan, "Maharashtra sees biggest 1-day Covid case spike in 70 days; curbs slapped in 7 districts," *Hindustan Times*, Feb. 18, 2021. https://www.hindustantimes.com/cities/mumbai-news/maharashtra-sees-biggest-1-day-covid-case-spike-in-70-days-curbs-slapped-in-7-districts-101613587982988.html (accessed Aug. 27, 2021).

[6] F. Malik, "Maharashtra crosses 5,000 Covid cases after 76 days, strict measures imposed," *Hindustan Times*, Feb. 19, 2021. https://www.hindustantimes.com/cities/mumbai-news/maharashtra-crosses-5-000-covid-cases-after-76-days-strict-measures-imposed-101613674784270.html (accessed Aug. 27, 2021).

[7] T. Venkatraman, "Maharashtra districts asked to ramp up measures to curb Covid surge," *Hindustan Times*, Mar. 10, 2021. https://www.hindustantimes.com/cities/mumbai-news/maharashtra-districts-asked-to-ramp-up-measures-to-curb-covid-surge-101615316004370.html (accessed Aug. 27, 2021).

[8] "No entry in Mumbai shopping malls without Covid-19 negative report from today," *Hindustan Times*, Mar. 22, 2021. https://www.hindustantimes.com/cities/mumbai-news/no-entry-in-mumbai-shopping-malls-without-covid-19-negative-report-from-today-101616382209468.html (accessed Aug. 27, 2021).

[9] P. Singh, “Covid-19: Night curfew imposed in Maharashtra from March 28,” *Hindustan Times*, Mar. 26, 2021. https://www.hindustantimes.com/cities/mumbai-news/covid-19-night-curfew-imposed-in-maharashtra-from-march-28-101616769070432.html (accessed Aug. 27, 2021).
[10] “India expands vaccination drive, all above 45 years to be vaccinated from April 1,” *Mumbai Mirror*, Mar. 23, 2021. Accessed: Jun. 07, 2021. [Online]. Available: https://mumbaimirror.indiatimes.com/coronavirus/news/covid-19-india-expands-vaccination-drive-all-above-45-years-to-be-vaccinated-from-april-1/articleshow/81649964.cms
[11] K. Sarkar, “What CM Uddhav Thackeray said in Covid-19 address to Maharashtra,” *Hindustan Times*, Apr. 02, 2021. https://www.hindustantimes.com/cities/mumbai-news/what-cm-uddhav-thackeray-said-in-covid-19-address-to-maharashtra-101617380837736.html (accessed Aug. 27, 2021).
[12] PTI, “COVID-19: Weekend lockdown in Maharashtra, stricter curbs from Monday,” *The New Indian Express*, Apr. 04, 2021. Accessed: Jun. 07, 2021. [Online]. Available: https://www.newindianexpress.com/nation/2021/apr/04/covid-19-weekend-lockdown-in-maharashtra-stricter-curbs-from-monday-2285721.html
[13] “Odisha To Maharashtra: Six States Complain Of Vaccine Shortage, Centre Says ‘No Scarcity Anywhere’,” *Outlook*, Apr. 08, 2021. Accessed: Jun. 07, 2021. [Online]. Available: https://www.outlookindia.com/website/story/india-news-odisha-to-maharashtra-six-states-complain-of-vaccine-shortages-centre-says-no-scarcity-anywhere/379615
[14] “Madhya Pradesh govt announces night curfew amid Covid spike. Check details,” *mint*, Apr. 07, 2021. Accessed: Jun. 07, 2021. [Online]. Available: https://www.livemint.com/news/india/madhya-pradesh-govt-announces-night-curfew-in-urban-areas-check-details-11617808196014.html
[15] K. Sarkar, “Maharashtra to witness night curfew, weekend lockdown from Monday to control Covid-19 surge. A list of all curbs,” *Hindustan Times*, Apr. 04, 2021. https://www.hindustantimes.com/india-news/maharashtra-to-witness-night-curfew-weekend-lockdown-from-monday-to-control-covid-19-surge-a-list-of-all-curbs-101617540511250.html (accessed Aug. 27, 2021).
[16] K. Sarkar, “Maharashtra switches to ‘Break The Chain’ from ‘Mission Begin Again’ to curb Covid-19 spread. Check full list of curbs,” *Hindustan Times*, Apr. 04, 2021. https://www.hindustantimes.com/india-news/maharashtra-switches-to-break-the-chain-from-mission-begin-again-to-curb-covid-19-spread-check-full-list-of-restrictions-here-101617550067188.html (accessed Aug. 27, 2021).
[17] “Night Curfew In Urban Areas Of 8 J&K Districts Amid Surge In Covid Cases,” *NDTV.com*, Apr. 08, 2021. Accessed: Jun. 07, 2021. [Online]. Available: https://www.ndtv.com/india-news/night-curfew-in-urban-areas-of-8-districts-of-jammu-and-kashmir-amid-rising-covid-19-cases-2409420
[18] Julia Hollingsworth, Melissa Macaya, Melissa Mahtani, Veronica Rocha and Fernando Alfonso III, “The latest on the coronavirus pandemic and vaccines,” *CNN*, Apr. 09, 2021. Accessed: Jun. 07, 2021. [Online]. Available: https://edition.cnn.com/world/live-news/coronavirus-pandemic-vaccine-updates-04-09-21/h_b617a849bbb8ba5584bde255f7833080
[19] “Coronavirus | Lockdown in Chhattisgarh’s Raipur district from April 9 to 19,” *The Hindu*, Raipur, Apr. 07, 2021. Accessed: Jun. 07, 2021. [Online]. Available: https://www.thehindu.com/news/national/other-states/coronavirus-lockdown-in-chhattisgarhs-raipur-district-from-april-9-to-19/article34265131.ece

[20] T. H. N. Desk, “Coronavirus second wave | List of States that have imposed restrictions, curfew and lockdowns,” *The Hindu*, May 10, 2021. Accessed: Jun. 07, 2021. [Online]. Available: https://www.thehindu.com/news/national/coronavirus-second-wave-here-is-a-look-at-lockdowns-imposed-in-various-states/article34525655.ece
[21] Government of Karnataka, “Government Orders - COVID-19 INFORMATION PORTAL.” https://covid19.karnataka.gov.in/new-page/Government%20Orders/en (accessed Jun. 07, 2021).
[22] “Covid-19 2nd wave: Full list of weekend curfews, lockdowns imposed across states - Coronavirus Outbreak News.” https://www.indiatoday.in/coronavirus-outbreak/story/full-list-of-lockdown-curfews-across-india-1791896-2021-04-17 (accessed Aug. 27, 2021).
[23] “Lockdown in Delhi till April 26, health system may collapse with daily 25,000 Covid cases, says CM Kejriwal,” *India Today*, Apr. 19, 2021. Accessed: Jun. 07, 2021. [Online]. Available: https://www.indiatoday.in/cities/delhi/story/lockdown-in-delhi-till-april-26-health-system-may-collapse-kejriwal-1792519-2021-04-19
[24] “ICSE, ISC board exams deferred, Cambridge’s off | India News - Times of India,” *The Times of India*. https://timesofindia.indiatimes.com/india/icse-isc-board-exams-deferred-cambridges-off/articleshow/82110864.cms (accessed Aug. 27, 2021).
[25] Rahul V Pisharody, “Telangana Covid-19 Night Curfew: Here’s what is allowed and what isn’t,” *The Indian Express*, Apr. 20, 2021. Accessed: Jun. 07, 2021. [Online]. Available: https://indianexpress.com/article/india/telangana-covid-19-night-curfew-heres-what-is-allowed-and-what-isnt-7281397/
[26] “Jharkhand orders 7-day COVID-19 lockdown from 22 April; full list of what is allowed and what isn’t,” *Firstpost*, Apr. 20, 2021. Accessed: Jun. 07, 2021. [Online]. Available: https://www.firstpost.com/india/jharkhand-orders-7-day-covid-19-lockdown-from-22-april-full-list-of-what-is-allowed-and-what-is-not-9548451.html
[27] S. P. Gangan, “Maharashtra imposes full lockdown from today,” *Hindustan Times*, Apr. 22, 2021. https://www.hindustantimes.com/cities/mumbai-news/maharashtra-imposes-full-lockdown-from-today-101619034644371.html (accessed Aug. 27, 2021).
[28] “Complete lockdown in Puducherry this weekend,” *The Indian Express*, Apr. 21, 2021. Accessed: Jun. 07, 2021. [Online]. Available: https://indianexpress.com/article/india/complete-lockdown-in-puducherry-this-weekend-7282664/
[29] “Maharashtra brings back Covid-19 e-pass for inter-state, inter-district travel: Here’s how to apply,” *Hindustan Times*, Apr. 23, 2021. https://www.hindustantimes.com/cities/mumbai-news/maharashtra-brings-back-covid-19-e-pass-for-inter-state-inter-district-travel-here-s-how-to-apply-101619161303652.html (accessed Aug. 27, 2021).
[30] Victor Dasgupta, “Covid-19: Andhra Pradesh Govt To Impose Seven-Hour Night Curfew From April 24 | Details Here,” *India.com*, Apr. 23, 2021. Accessed: Jun. 07, 2021. [Online]. Available: https://www.india.com/news/india/covid-19-andhra-pradesh-govt-to-impose-seven-hour-night-curfew-from-april-24-details-here-4609178/

[31] Shivendra Srivastava, “UP govt imposes weekend lockdown across state, night curfew in districts with over 500 active cases,” *India Today*, Apr. 20, 2021. Accessed: Jun. 07, 2021. [Online]. Available: https://www.indiatoday.in/coronavirus-outbreak/story/up-govt-imposes-weekend-lockdown-across-state-night-curfew-in-districts-with-over-500-active-cases-1792981-2021-04-20
[32] “Covid-19 second wave: Here’s a list of states that have imposed full lockdown,” *The Indian Express*, May 09, 2021. Accessed: Jun. 07, 2021. [Online]. Available: https://indianexpress.com/article/india/covid-19-second-wave-heres-a-list-of-states-that-have-imposed-lockdowns-7306634/
[33] Anand Bodh, “Night curfew in Himachal Pradesh: Night curfew imposed in four districts of Himachal Pradesh,” *The Times of India*, Apr. 25, 2021. Accessed: Jun. 07, 2021. [Online]. Available: https://timesofindia.indiatimes.com/city/shimla/covid-19-night-curfew-imposed-in-four-districts-of-himachal-pradesh/articleshow/82241827.cms
[34] “Karnataka ‘close-down’: What’s allowed, what’s not,” *Deccan Herald*, Apr. 26, 2021. Accessed: Jun. 07, 2021. [Online]. Available: https://www.deccanherald.com/state/top-karnataka-stories/karnataka-close-down-whats-allowed-whats-not-979054.html
[35] H. Sabarwal, “Covid: Maharashtra extends lockdown-like restrictions till May 15,” *Hindustan Times*, Apr. 29, 2021. https://www.hindustantimes.com/cities/mumbai-news/covid-maharashtra-extends-lockdown-like-restrictions-till-may-15-101619706340267.html (accessed Aug. 27, 2021).
[36] “Nagaland to impose partial lockdown for a fortnight from April 30,” *Outlook*, Apr. 27, 2021. Accessed: Jun. 07, 2021. [Online]. Available: https://www.outlookindia.com/newsscroll/nagaland-to-impose-partial-lockdown-for-a-fortnight-from-april-30/2072484
[37] “Nagaland Extends Total Lockdown Till June 11 as Covid-19 Cases Spike,” *CNN-News 18*, May 28, 2021. Accessed: Jun. 07, 2021. [Online]. Available: https://www.news18.com/news/india/nagaland-extends-total-lockdown-till-june-11-as-covid-19-cases-spike-3787433.html
[38] “Everyone above the age of 18 to be eligible to get vaccine from May 1: Government,” *The Economic Times*, Apr. 20, 2021. Accessed: Jun. 07, 2021. [Online]. Available: https://economictimes.indiatimes.com/news/india/everyone-above-the-age-of-18-to-be-eligible-to-get-vaccine-from-may-1-government/articleshow/82146974.cms?from=mdr
[39] “Mizoram: Eight-day lockdown imposed in Aizawl, other district headquarters,” *mint*, May 02, 2021. Accessed: Jun. 07, 2021. [Online]. Available: https://www.livemint.com/news/india/mizoram-eight-day-lockdown-imposed-in-aizawl-other-district-headquarters-11619922863146.html
[40] “Mizoram extends lockdown in Aizawl till June 6,” *The New Indian Express*, May 29, 2021. Accessed: Jun. 07, 2021. [Online]. Available: https://www.newindianexpress.com/nation/2021/may/29/mizoram-extends-lockdown-in-aizawl-till-june-6-2309255.html
[41] “Punjab imposes lockdown-like curbs till May 15; check out what’s allowed, what’s not,” *Business Today*, May 03, 2021. Accessed: Jun. 07, 2021. [Online]. Available: https://www.businesstoday.in/coronavirus/punjab-imposes-lockdown-like-curbs-till-may-15-check-out-what-allowed-what-not/story/438098.html
[42] Debabrata Mohapatra, “Covid: Odisha announces 14-day lockdown from May 5,” *The Times of India*, May 02, 2021. Accessed: Jun. 08, 2021. [Online]. Available: https://timesofindia.indiatimes.com/city/bhubaneswar/14-day-lockdown-in-odisha-from-may-5/articleshow/82352596.cms

[43] Debabrata Mohapatra, “Odisha lockdown news: Lockdown in Odisha extended till June 17,” *The Times of India*, May 31, 2021. Accessed: Jun. 07, 2021. [Online]. Available: https://timesofindia.indiatimes.com/city/bhubaneswar/lockdown-extended-in-odisha-till-june-17/articleshow/83106749.cms
[44] “Andhra Pradesh Partial Curfew: Partial curfew in Andhra Pradesh for 14 days starting from May 5,” *The Economic Times*, May 03, 2021. Accessed: Jun. 07, 2021. [Online]. Available: https://economictimes.indiatimes.com/news/india/partial-curfew-in-andhra-pradesh-for-14-days-starting-may-5/articleshow/82368569.cms
[45] “Night curfew in Gujarat till April 30 amid surge in COVID-19 cases,” *Business Today*, Apr. 07, 2021. Accessed: Jun. 07, 2021. [Online]. Available: https://www.businesstoday.in/coronavirus/night-curfew-in-gujarat-till-april-30-amid-surge-in-covid-19-cases/story/435920.html
[46] “Telangana lockdown for 10 days starting tomorrow,” *The Economic Times*, May 11, 2021. Accessed: Jun. 07, 2021. [Online]. Available: https://economictimes.indiatimes.com/news/india/covid-19-telangana-announces-10-day-lockdown-from-may-12/articleshow/82546448.cms?from=mdr
[47] D. Sharma, “Lockdown-like curbs in Maharashtra till June 1. What’s allowed, what’s not,” *Hindustan Times*, May 13, 2021. https://www.hindustantimes.com/cities/mumbai-news/lockdownlike-curbs-in-maharashtra-till-june-1-what-s-allowed-what-s-not-101620894717555.html (accessed Aug. 27, 2021).
[48] “Maharashtra to compile database of mucormycosis to assess its spread,” *Hindustan Times*, May 13, 2021. https://www.hindustantimes.com/cities/mumbai-news/maharashtra-to-compile-database-of-mucormycosis-to-assess-its-spread-101620877579460.html (accessed Aug. 27, 2021).
[49] S. K. J. May 23, 2021UPDATED: May 23, and 2021 20:35 Ist, “Lockdown in Rajasthan extended till June 8,” *India Today*. https://www.indiatoday.in/coronavirus-outbreak/story/lockdown-in-rajasthan-extended-till-june-8-1806049-2021-05-23 (accessed Aug. 26, 2021).
[50] “Maharashtra districts with poor Covid-19 vaccination coverage to get more doses,” *Hindustan Times*, May 27, 2021. https://www.hindustantimes.com/cities/mumbai-news/maharashtra-districts-with-poor-covid-19-vaccination-coverage-to-get-more-doses-101622131785481.html (accessed Aug. 27, 2021).
[51] H. Sabarwal, “Maharashtra announces paediatric task force to prepare for 3rd wave of Covid-19,” *Hindustan Times*, May 27, 2021. https://www.hindustantimes.com/cities/mumbai-news/maharashtra-announces-paediatric-task-force-to-prepare-for-3rd-wave-of-covid19-101622107934885.html (accessed Aug. 27, 2021).
[52] S. P. Gangan, “Maharashtra government extends lockdown to June 15,” *Hindustan Times*, May 31, 2021. https://www.hindustantimes.com/cities/mumbai-news/maharashtra-government-extends-lockdown-to-june-15-101622399442158.html (accessed Aug. 27, 2021).
[53] I. com N. Desk, “Himachal Pradesh Extends ‘Corona Curfew’ Till June 14,” *India News, Breaking News | India.com*, Jun. 05, 2021. https://www.india.com/news/india/himachal-pradesh-lockdown-2021-news-today-june-5-2021-corona-curfew-extended-till-june-14-shimla-news-jai-ram-thakur-check-details-4718111/ (accessed Aug. 26, 2021).

[54] M. Ray, “Maharashtra to bring in 5-level unlock plan from Monday: Rules explained,” *Hindustan Times*, Jun. 05, 2021. https://www.hindustantimes.com/cities/mumbai-news/maharashtra-to-bring-in-5-level-unlock-plan-from-monday-rules-explained-101622879112587.html (accessed Aug. 27, 2021).
[55] I. com N. Desk, “Lockdown in Madhya Pradesh Extended Till June 15 With Relaxations | Check New Restrictions,” *India News, Breaking News | India.com*, Jun. 08, 2021. https://www.india.com/madhya-pradesh/madhya-pradesh-lockdown-unlock-news-today-8-june-2021-lockdown-in-mp-extended-till-june-15-shivraj-singh-chouhan-check-full-list-if-unlock-guidelines-in-ujjain-and-bhopal-4724196/ (accessed Aug. 26, 2021).
[56] I. com N. Desk, “Rajasthan Unlock: Lockdown Norms Relaxed From Today. Shops And Markets to Open Till 4 PM | Revised Guidelines Here,” *India News, Breaking News | India.com*, Jun. 08, 2021. https://www.india.com/news/india/rajasthan-lockdown-news-2021-rajasthan-unlock-lockdown-lifted-norms-relaxed-check-new-guidelines-for-shops-restaurants-travel-full-list-4723199/ (accessed Aug. 26, 2021).
[57] “Bihar Ends Lockdown from Today with Some Restrictions; Here is What’s Open and What’s Closed,” *News18*, Jun. 09, 2021. https://www.news18.com/news/india/bihar-lockdown-nitish-kumar-covid-19-coronavirus-patna-3822656.html (accessed Aug. 26, 2021).
[58] D. A. W. J. June 27, 2021UPDATED: June 27, and 2021 10:28 Ist, “Rajasthan govt relaxes curbs as Covid-19 cases dip. Here’s what’s allowed from Monday,” *India Today*. https://www.indiatoday.in/india/rajasthan/story/dip-covid-cases-rajasthan-relaxes-curbs-details-1819893-2021-06-27 (accessed Aug. 26, 2021).
[59] N. Kamath, “Covid in Maharashtra: Stricter curbs from Monday,” *Hindustan Times*, Jun. 27, 2021. https://www.hindustantimes.com/cities/mumbai-news/covid-in-maharashtra-stricter-curbs-from-monday-101624817587961.html (accessed Aug. 27, 2021).
[60] M. Rajeev, “Restrictions in Telangana extended till July 31,” *The Hindu*, Hyderabad, Jul. 01, 2020. Accessed: Aug. 26, 2021. [Online]. Available: https://www.thehindu.com/news/national/telangana/restrictions-in-telangana-extended-till-july-31/article31961013.ece
[61] Bhandary, “Schools in non-Covid zones can reopen for Class 8 to 12 from July 15: GR,” *Hindustan Times*, Jul. 08, 2021. https://www.hindustantimes.com/cities/mumbai-news/schools-in-non-covid-zones-can-reopen-for-class-8-to-12-from-july-15-gr-101625682860932.html (accessed Aug. 27, 2021).
[62] R. Hardaha, “COVID: Weekend lockdown lifted in Jammu and Kashmir,” Jul. 25, 2021. https://www.indiatvnews.com/news/india/jammu-kashmir-lockdown-update-covid-unlock-guidelines-weekend-curfew-relaxations-721906 (accessed Aug. 26, 2021).
[63] N. Kamath, “Maharashtra set to relax some restrictions this week,” *Hindustan Times*, Jul. 25, 2021. https://www.hindustantimes.com/cities/mumbai-news/maharashtra-set-to-relax-some-restrictions-this-week-101627237588717.html (accessed Aug. 27, 2021).
[64] “Chhattisgarh Lockdown News: Chhattisgarh extends lockdown till August 6 in affected districts | Raipur News - Times of India.” https://timesofindia.indiatimes.com/city/raipur/covid-19-chhattisgarh-extends-lockdown-till-august-6-in-affected-districts/articleshow/77200593.cms (accessed Aug. 26, 2021).

[65] “COVID-19: Jharkhand allows schools to conduct 1X-XII classes; extends lockdown-like curbs,” *The New Indian Express*. https://www.newindianexpress.com/nation/2021/jul/30/covid-19-jharkhand-allows-schools-to-conduct-1x-xii-classes-extends-lockdown-like-curbs-2337812.html (accessed Aug. 26, 2021).

[66] “Nagaland Extends Coronavirus Lockdown Till August 31 As Cases Rise,” *NDTV.com*. https://www.ndtv.com/india-news/nagaland-extends-coronavirus-lockdown-till-august-31-as-cases-rise-2271590 (accessed Aug. 26, 2021).

[67] “Karnataka extends Covid-19 containment measures till August 16 – Check the new guidelines here.” https://www.timesnownews.com/india/article/karnataka-extends-covid-19-containment-measures-till-august-16-check-the-new-guidelines-here/793070 (accessed Aug. 26, 2021).

[68] “Punjab Schools To Reopen For All Classes From August 2,” *NDTV.com*. https://www.ndtv.com/india-news/punjab-schools-to-reopen-for-all-classes-from-august-2-2499567 (accessed Aug. 26, 2021).

[69] “Jammu and Kashmir lockdown: Schools, educational institutions to remain closed, only 25 people allowed at gatherings,” *Zee News*, Aug. 01, 2021. https://zeenews.india.com/india/jammu-and-kashmir-lockdown-schools-educational-institutions-to-remain-closed-only-25-people-allowed-at-gatherings-2381010.html (accessed Aug. 26, 2021).

[70] I. com N. Desk, “Unlock 3.0 in Himachal: Lockdown in Containment Zones Extended Till August 31, No Night Curfew,” *India News, Breaking News | India.com*, Aug. 01, 2020. https://www.india.com/news/india/unlock-3-0-in-himachal-pradesh-state-extends-lockdown-in-containment-zones-till-august-31-no-night-curfew-details-here-4099676/ (accessed Aug. 26, 2021).

[71] Livemint, “Odisha relaxes Covid-19 curbs; here’s list of things allowed from August 1,” *mint*, Aug. 01, 2021. https://www.livemint.com/news/india/odisha-relaxes-covid-19-curbs-here-s-list-of-things-allowed-from-august-1-11627776552457.html (accessed Aug. 26, 2021).

[72] “Maharashtra Lockdown Guidelines: Maharashtra relaxes Covid-19 restrictions except for 11 districts; Check what’s allowed | Mumbai News - Times of India,” *The Times of India*. https://timesofindia.indiatimes.com/city/mumbai/maharashtra-relaxes-covid-19-restrictions-except-for-11-districts-heres-whats-allowed/articleshow/84976210.cms (accessed Aug. 26, 2021).

[73] A. N. Bureau, “Bihar Unlock: Shops, Schools, Cinema Halls To Reopen From Aug 7 With Curbs,” Aug. 04, 2021. https://news.abplive.com/news/india/bihar-unlock-from-7-to-25-august-know-government-corona-guideline-restrictions-check-details-1474166 (accessed Aug. 26, 2021).

[74] A. N. Bureau, “Kerala Govt Extends Covid Guidelines Amid Surge In Cases,” Aug. 04, 2021. https://news.abplive.com/health/kerala-extends-covid-restrictions-amid-surge-in-cases-eases-lockdown-curbs-details-here-1474102 (accessed Aug. 26, 2021).

[75] “Karnataka extends night curfew timing, schools for class 9-12 to begin from August 23,” *Zee News*, Aug. 06, 2021. https://zeenews.india.com/karnataka/karnataka-bengaluru-lockdown-update-night-curfew-timing-extended-schools-for-class-9-12-to-begin-from-august-23-2382405.html (accessed Aug. 26, 2021).

[76] Livemint, “Covid: J&K restricts public gatherings to 25, limit to be relaxed only for I-Day,” *mint*, Aug. 08, 2021. https://www.livemint.com/news/india/covid-jammu-kashmir-restricts-public-gatherings-to-25-limit-to-be-relaxed-only-for-independence-day-11628415852877.html (accessed Aug. 26, 2021).

[77] I. com N. Desk, “Odisha Relaxes Weekend Curfew Timing in 3 Towns For Independence Day 2021 | Check Details,” *India News, Breaking News | India.com*, Aug. 08, 2021. https://www.india.com/news/india/odisha-relaxes-weekend-curfew-timing-in-3-towns-for-independence-day-2021-check-details-bhubaneswar-cuttack-and-puri-4875284/ (accessed Aug. 26, 2021).
[78] “News headlines for Aug 8: Haryana extends lockdown; CM Kejriwal launches free food programme & other top news.” https://www.timesnownews.com/india/article/news-headlines-for-aug-8-haryana-extends-lockdown-cm-kejriwal-launches-free-food-programme-other-top-news/796404 (accessed Aug. 26, 2021).
[79] “Pune Lockdown guidelines: Maharashtra: Timings of shops and restaurants extended in Pune, malls to open for fully vaccinated - The Economic Times.” https://economictimes.indiatimes.com/news/india/maharashtra-timings-of-shops-restaurants-extended-in-pune-malls-to-open-for-fully-vaccinated/articleshow/85153094.cms (accessed Aug. 26, 2021).
[80] “Assam lockdown: Partial curfew imposed statewide as COVID-19 positivity rate rises,” *Zee News*, Aug. 09, 2021. https://zeenews.india.com/india/assam-lockdown-partial-curfew-imposed-statewide-as-covid-19-positivity-rate-rises-2383282.html (accessed Aug. 26, 2021).
[81] “UP Lockdown: No lockdown on Saturdays in Uttar Pradesh, Sunday restrictions to continue | Lucknow News - Times of India,” *The Times of India*. https://timesofindia.indiatimes.com/city/lucknow/covid-19-no-curfew-on-saturdays-in-uttar-pradesh-sunday-restrictions-to-continue/articleshow/85241564.cms (accessed Aug. 26, 2021).
[82] I. com N. Desk, “All Schools in Himachal Pradesh to Remain Closed Till August 22,” *India News, Breaking News | India.com*, Aug. 11, 2021. https://www.india.com/news/india/all-schools-in-himachal-pradesh-to-remain-closed-till-august-22-4881986/ (accessed Aug. 26, 2021).
[83] “West Bengal Lockdown News: Covid-restrictions extended till August 30 with relaxations | Kolkata News - Times of India,” *The Times of India*. https://timesofindia.indiatimes.com/city/kolkata/west-bengal-covid-restrictions-extended-till-august-30-with-relaxations/articleshow/85270674.cms (accessed Aug. 26, 2021).
[84] “Puducherry Govt Extends Covid-19 Lockdown Till Aug 31,” *News18*, Aug. 15, 2021. https://www.news18.com/news/india/puducherry-govt-extends-covid-19-lockdown-till-aug-31-4088942.html (accessed Aug. 26, 2021).
[85] N. P. B. August 24, 2021UPDATED: August 24, and 2021 14:59 Ist, “Karnataka govt asks Bengaluru IT firms to allow WFH till 2022 to avoid traffic mess,” *India Today*. https://www.indiatoday.in/cities/bengaluru/story/karnataka-govt-asks-bengaluru-it-firms-to-allow-wfh-till-2022-1844683-2021-08-24 (accessed Aug. 26, 2021).
[86] I. com N. Desk, “Andhra Pradesh Extends Night Curfew Till September 4. Check New COVID-19 Guidelines,” *India News, Breaking News | India.com*, Aug. 20, 2021. https://www.india.com/news/india/andhra-pradesh-extends-night-curfew-till-september-4-check-new-covid-19-guidelines-4901219/ (accessed Aug. 26, 2021).
[87] “Tamil Nadu extends Covid-19 curbs till September 6, but gives fresh relaxations,” *Hindustan Times*, Aug. 21, 2021. https://www.hindustantimes.com/india-news/tamil-nadu-extends-covid-19-curbs-till-september-6-but-gives-fresh-relaxations-101629553676993.html (accessed Aug. 26, 2021).

[88] I. com N. Desk, “Delhi Lockdown: Kejriwal Govt Lifts Restrictions, Allows Markets to Open as Per Normal Timing,” *India News, Breaking News | India.com*, Aug. 21, 2021. https://www.india.com/news/india/delhi-lockdown-unlock-news-today-arvind-kejriwal-govt-lifts-restrictions-allows-markets-to-open-as-per-normal-timing-from-monday-check-latest-guidelines-4903534/ (accessed Aug. 26, 2021).
[89] “Coronavirus News Highlights: Goa Govt extends COVID-induced curfew till August 30; Kerala reports 10,402 new cases,” *The Financial Express*, Aug. 23, 2021. https://www.financialexpress.com/lifestyle/health/coronavirus-india-latest-update-live-delhi-allows-markets-to-stay-open-beyond-8-pm-tamil-nadu-extends-covid-lockdown-by-2-weeks/2315198/ (accessed Aug. 26, 2021).
[90] T. H. N. Desk, “Coronavirus updates | August 24, 2021,” *The Hindu*, Aug. 24, 2021. Accessed: Aug. 26, 2021. [Online]. Available: https://www.thehindu.com/news/national/coronavirus-live-august-24-2021-updates/article36071918.ece
[91] “Gujarat Relaxes Night Curfew Time in These 8 Cities for Janmashtami, Ganesh Festivals,” *News18*, Aug. 24, 2021. https://www.news18.com/news/india/guj-relaxes-night-curfew-time-in-these-8-cities-for-janmashtami-ganesh-festivals-4123667.html (accessed Aug. 26, 2021).
[92] “Kerala extends weekend lockdown amid worrying surge in Covid-19 cases.” https://www.telegraphindia.com/india/kerala-extends-weekend-lockdown-amid-worrying-surge-in-covid-19-cases/cid/1824370 (accessed Aug. 26, 2021).
[93] S. Kashyap, “School Reopening News: List of states that announced school reopen dates in November 2021,” *https://www.oneindia.com*, Nov. 01, 2021. https://www.oneindia.com/india/school-reopening-news-list-of-states-that-announced-school-reopen-dates-in-november-2021-3330419.html (accessed Mar. 05, 2022).
[94] “India to tighten testing for tourists as new COVID variant found | Coronavirus pandemic News | Al Jazeera.” https://www.aljazeera.com/news/2021/11/26/india-covid-testing-tourists-south-africa-variant (accessed Mar. 05, 2022).
[95] “After 20 months, Maharashtra govt lifts all lockdown restrictions | Business Standard News.” https://www.business-standard.com/article/current-affairs/after-20-months-maharashtra-govt-lifts-all-lockdown-restrictions-121112700727_1.html (accessed Mar. 05, 2022).
[96] “Schools in Chandigarh closed from December 20 | Chandigarh News - Times of India.” https://timesofindia.indiatimes.com/city/chandigarh/schools-in-chandigarh-closed-from-december-20/articleshow/88355895.cms (accessed Mar. 05, 2022).
[97] P. J. N. D. December 28, 2021UPDATED: December 28, and 2021 22:08 Ist, “Yellow alert in Delhi, schools and colleges to be closed immediately,” *India Today*. https://www.indiatoday.in/education-today/news/story/yellow-alert-in-delhi-schools-and-colleges-to-be-closed-immediately-1893302-2021-12-28 (accessed Mar. 05, 2022).
[98] “Bangladesh starts COVID vaccination drive.” https://www.aljazeera.com/news/2021/1/28/bangladesh-starts-covid-vaccination-drive (accessed Jan. 21, 2022).
[99] P. T. of India, “Bangladesh issues Covid-19 guidelines as cases surge,” *Business Standard India*, Mar. 29, 2021. Accessed: Jan. 21, 2022. [Online]. Available: https://www.business-standard.com/article/current-affairs/bangladesh-issues-covid-19-guidelines-as-cases-surge-121032900754_1.html

[100] M. A. M. I. K. L. N. S. W. D. S. A. D. J. H. Developer), “Cox’s Bazar falls silent as restrictions on tourists kick in,” *unb.com.bd*. https://unb.com.bd/category/Bangladesh/coxs-bazar-falls-silent-as-restrictions-on-tourists-kick-in/67025 (accessed Jan. 21, 2022).
[101] “International flights to remain suspended from 14 April.” https://www.tbsnews.net/economy/aviation/bangladesh-suspends-international-flights-14-april-230227 (accessed Jan. 21, 2022).
[102] “Bangladesh goes into full lockdown for a week from Monday,” *Dhaka Tribune*, Apr. 03, 2021. https://archive.dhakatribune.com/bangladesh/2021/04/03/quader-govt-to-announce-week-long-countrywide-lockdown-from-monday (accessed Jan. 21, 2022).
[103] “Bangladesh to go into week-long hard lockdown from April 14,” *Dhaka Tribune*, Apr. 09, 2021. https://archive.dhakatribune.com/health/coronavirus/2021/04/09/government-considering-week-long-full-lockdown-from-april-14 (accessed Jan. 21, 2022).
[104] “Bangladesh closes border with India amid rise in COVID-19 cases,” *The Hindu*, Dhaka, Apr. 25, 2021. Accessed: Jan. 21, 2022. [Online]. Available: https://www.thehindu.com/news/national/bangladesh-closes-border-with-india-amid-rise-in-covid-19-cases/article34407522.ece
[105] “Bangladesh extends restrictions on public movement till May 16,” *Dhaka Tribune*, May 03, 2021. https://archive.dhakatribune.com/bangladesh/2021/05/03/lockdown-extended-till-may-16 (accessed Jan. 21, 2022).
[106] “Ban on bus, train, launch services lifted,” *Dhaka Tribune*, May 23, 2021. https://archive.dhakatribune.com/bangladesh/2021/05/23/ban-on-bus-train-launch-services-lifted (accessed Jan. 21, 2022).
[107] S. Correspondent and bdnews24.com, “Bangladesh lowers minimum age for COVID vaccines to 35 years.” https://bdnews24.com/health/2021/07/05/bangladesh-lowers-minimum-age-for-covid-vaccines-to-35-years (accessed Mar. 01, 2022).
[108] “Seven districts put into strict lockdown to safeguard Dhaka,” *Dhaka Tribune*, Jun. 21, 2021. https://archive.dhakatribune.com/bangladesh/2021/06/21/covid-19-lockdown-in-seven-districts-from-june-22 (accessed Jan. 21, 2022).
[109] ANI, “Bangladesh to impose nationwide lockdown from July 1 instead of June 28,” *Business Standard India*, Jun. 27, 2021. Accessed: Jan. 21, 2022. [Online]. Available: https://www.business-standard.com/article/international/bangladesh-to-impose-nationwide-lockdown-from-july-1-instead-of-june-28-121062700318_1.html
[110] A. Agency, “Bangladesh relaxes COVID-19 lockdown due to Eid al-Adha holiday,” *Daily Sabah*, Jul. 13, 2021. https://www.dailysabah.com/world/asia-pacific/bangladesh-relaxes-covid-19-lockdown-due-to-eid-al-adha-holiday (accessed Mar. 01, 2022).
[111] “DGHS: Age limit for Covid-19 vaccination lowered to 30 years,” *Dhaka Tribune*, Jul. 19, 2021. https://archive.dhakatribune.com/health/coronavirus/2021/07/19/dghs-age-limit-for-covid-19-vaccination-lowered-to-30-years (accessed Mar. 01, 2022).
[112] “Bangladesh lowers Covid-19 vaccine age limit to 18 years.” https://www.dhakatribune.com/bangladesh/2021/10/20/bangladesh-lowers-covid-19-vaccine-age-limit-to-18-years (accessed Mar. 01, 2022).

[113] “Bangladeshis rush back to work as factories reopen despite virus surge,” *The Economic Times*, Jul. 31, 2021. Accessed: Mar. 01, 2022. [Online]. Available: https://economictimes.indiatimes.com/news/international/business/bangladeshis-rush-back-to-work-as-factories-reopen-despite-virus-surge/articleshow/84918964.cms
[114] “Bangladesh govt announces to lift lockdown from Aug 11,” *NewsOnAIR* -, Aug. 08, 2021. https://newsonair.com/2021/08/08/bangladesh-govt-announces-to-lift-lockdown-from-aug-11/ (accessed Mar. 01, 2022).
[115] F. Mahmud, “Bangladesh reopens schools after 18-month COVID shutdown.” https://www.aljazeera.com/news/2021/9/13/bangladesh-reopens-schools-after-18-month-covid-shutdown (accessed Mar. 05, 2022).
[116] S. Correspondent and bdnews24.com, “UGC launches COVID vaccine registration weblink for university students.” https://bdnews24.com/education/2021/09/16/ugc-launches-covid-vaccine-registration-weblink-for-university-students (accessed Mar. 01, 2022).
[117] “Covid vaccination campaign for school students kicks off.” https://www.dhakatribune.com/coronavirus/2021/11/01/covid-vaccination-campaign-for-school-students-kicks-off (accessed Mar. 01, 2022).
[118] “Bangladesh suspends travel from South Africa amid fears of new COVID-19 variant Omicron,” *The New Indian Express*. https://www.newindianexpress.com/world/2021/nov/27/bangladesh-suspends-travel-fromsouth-africa-amid-fears-of-new-covid-19-variant-spread-2388909.html (accessed Mar. 05, 2022).
[119] A. Chaudhary, “COVID-19 vaccination drive to commence from Wednesday in Nepal,” *The Himalayan Times*, Jan. 24, 2021. https://thehimalayantimes.com/nepal/covid-19-vaccination-drive-to-commence-from-wednesday-in-nepal (accessed Jan. 22, 2022).
[120] “Government decides to close schools in city areas until May 14.” https://kathmandupost.com/national/2021/04/19/government-decides-to-close-schools-in-city-areas-until-may-14 (accessed Mar. 05, 2022).
[121] “Nepal’s lockdown 2.0, new Covid curbs on travel.” https://kathmandupost.com/money/2021/04/28/explained-nepal-s-lockdown-2-0-new-covid-curbs-on-travel (accessed Jan. 22, 2022).
[122] “Kathmandu-based oxygen plants suspend supplying oxygen to industries.” https://kathmandupost.com/valley/2021/04/30/kathmandu-based-oxygen-plants-suspend-supplying-oxygen-to-industries (accessed Jan. 22, 2022).
[123] “Nepal to close 22 entry points with India amid COVID-19 surge,” *The Hindu*, Kathmandu, May 01, 2021. Accessed: Jan. 22, 2022. [Online]. Available: https://www.thehindu.com/news/international/nepal-to-close-22-entry-points-with-india-amid-covid-19-surge/article34457578.ece
[124] “Nepal shuts all international flights save two a week between Kathmandu and Delhi.” https://kathmandupost.com/national/2021/05/03/nepal-shuts-all-international-flights-save-two-a-week-between-kathmandu-and-delhi (accessed Jan. 22, 2022).
[125] “Covid crisis reaches Nepal with a surge in cases and hospitalizations,” *NBC News*. https://www.nbcnews.com/health/health-news/covid-crisis-reaches-nepal-surge-cases-hospitalizations-n1267419 (accessed Jan. 22, 2022).

[126] “Nepal extends lockdown in Kathmandu Valley with relaxed provisions - Xinhua | English.news.cn.” http://www.xinhuanet.com/english/asiapacific/2021-06/21/c_1310018660.htm (accessed Jan. 22, 2022).
[127] “Nepal to resume domestic and international flights with restrictions: Civil aviation ministry,” *The Economic Times*, Jun. 23, 2021. Accessed: Mar. 05, 2022. [Online]. Available: https://economictimes.indiatimes.com/news/international/world-news/nepal-to-resume-domestic-and-international-flights-with-restrictions-civil-aviation-ministry/articleshow/83780869.cms
[128] T. H. T. Online, “Kathmandu metropolis inoculating +2 students above 18 years from today, 40+ population to get vaccinated too,” *The Himalayan Times*, Aug. 20, 2021. https://thehimalayantimes.com/kathmandu/kathmandu-metropolis-inoculating-2-students-above-18-years-from-today-40-population-to-get-vaccinated-too (accessed Mar. 02, 2022).
[129] “Four-month lockdown ends in Nepal’s Kathmandu Valley.” http://www.xinhuanet.com/english/asiapacific/2021-09/01/c_1310162115.htm (accessed Mar. 01, 2022).
[130] “Schools in Nepal’s Kathmandu Valley reopen amid pandemic.” http://www.news.cn/english/2021-09/17/c_1310194226.htm (accessed Mar. 05, 2022).
[131] “COVID-19: Nepal starts vaccinating nationals aged under 18,” *ANI News*. https://www.aninews.in/news/world/asia/covid-19-nepal-starts-vaccinating-nationals-aged-under-1820211114220643/ (accessed Mar. 01, 2022).
[132] G. Sharma, “Nepal to ban arrivals from 8 African countries, Hong Kong over Omicron fears,” *Reuters*, Dec. 03, 2021. Accessed: Mar. 05, 2022. [Online]. Available: https://www.reuters.com/business/healthcare-pharmaceuticals/nepal-ban-arrivals-8-african-countries-hong-kong-over-omicron-fears-2021-12-03/
[133] “Schools, universities in Pakistan reopen after coronavirus-imposed hiatus,” *Arab News PK*, Feb. 01, 2021. https://arab.news/8wqnv (accessed Mar. 05, 2022).
[134] “Pakistan begins COVID-19 vaccination programme,” *The Hindu*, Islamabad, Feb. 03, 2021. Accessed: Jan. 22, 2022. [Online]. Available: https://www.thehindu.com/news/international/pakistan-begins-covid-19-vaccination-programme/article33743052.ece
[135] I. Junaidi, “NCC may consider imposing further curbs today,” *DAWN.COM*, Apr. 23, 2021. https://www.dawn.com/news/1619846 (accessed Jan. 23, 2022).
[136] D. com | N. Siddiqui, “Education institutes in 7 Punjab cities to close for 2 weeks from March 15,” *DAWN.COM*, Mar. 10, 2021. https://www.dawn.com/news/1611743 (accessed Mar. 05, 2022).
[137] “COVID-19: Pakistan imposes smart lockdown in worst-hit areas,” *The New Indian Express*. https://www.newindianexpress.com/world/2021/mar/18/covid-19-pakistan-imposes-smart-lockdown-in-worst-hit-areas-2278343.html (accessed Jan. 22, 2022).
[138] I. Junaidi, “10 cities put under stiff lockdown till April 11,” *DAWN.COM*, Mar. 23, 2021. https://www.dawn.com/news/1614089 (accessed Jan. 22, 2022).
[139] “Pakistan reduces inbound international flights to 20%,” *The Express Tribune*, May 01, 2021. http://tribune.com.pk/story/2297685/pakistan-reduces-inbound-international-flights-to-20 (accessed Jan. 22, 2022).

[140] P. Beaumont and S. M. Baloch, “India’s neighbours close borders as Covid wave spreads across region,” *The Guardian*, May 05, 2021. Accessed: Jan. 22, 2022. [Online]. Available: https://www.theguardian.com/world/2021/may/05/indias-neighbours-close-borders-as-covid-wave-spreads-across-region
[141] ANI, “Pak imposes 10-day countrywide lockdown from May 8 to control Covid,” *Business Standard India*, May 08, 2021. Accessed: Jan. 22, 2022. [Online]. Available: https://www.business-standard.com/article/current-affairs/pak-imposes-10-day-countrywide-lockdown-from-may-8-to-control-covid-121050800752_1.html
[142] “Pakistan eases certain virus restrictions, allows reopening of schools from May 24 - GulfToday.” https://www.gulftoday.ae/news/2021/05/19/pakistan-eases-certain-virus-restrictions-allows-reopening-of-schools-from-may-24 (accessed Mar. 05, 2022).
[143] Reuters, “Pakistan opens coronavirus vaccination drive to all adults,” *Reuters*, May 26, 2021. Accessed: Mar. 05, 2022. [Online]. Available: https://www.reuters.com/business/healthcare-pharmaceuticals/pakistan-opens-coronavirus-vaccination-drive-all-adults-2021-05-26/
[144] Z. ur-Rehman, “Unvaccinated in Pakistan? You might lose your cellphone service.,” *The New York Times*, Jun. 15, 2021. Accessed: Jan. 22, 2022. [Online]. Available: https://www.nytimes.com/2021/06/15/world/pakistan-vaccine-cellphones.html
[145] Dawn.com, “Schools to remain closed till Aug 30: Sindh govt,” *DAWN.COM*, Aug. 20, 2021. https://www.dawn.com/news/1641644 (accessed Mar. 05, 2022).
[146] Reuters, “Pakistan’s largest city to undergo partial lockdown to curb virus spread,” *Reuters*, Jul. 30, 2021. Accessed: Mar. 05, 2022. [Online]. Available: https://www.reuters.com/world/asia-pacific/pakistans-largest-city-undergo-partial-lockdown-curb-virus-spread-2021-07-30/
[147] “Pakistan bans travel from 6 countries, Hong Kong amid concern over new Covid variant - Pakistan - DAWN.COM.” https://www.dawn.com/news/1660582 (accessed Mar. 05, 2022).
[148] N. Siddiqui, “Pakistan extends travel ban to 9 more countries, tightens up protocols for 13 others,” *DAWN.COM*, Dec. 06, 2021. https://www.dawn.com/news/1662249 (accessed Mar. 05, 2022).
[149] A. B. C. News, “India donates first 500,000 doses of vaccine to Sri Lanka,” *ABC News*. https://abcnews.go.com/Health/wireStory/india-donates-500000-doses-vaccine-sri-lanka-75532989 (accessed Jan. 22, 2022).
[150] “Sri Lanka to reopen all schools from March 29 - Times of India.” https://timesofindia.indiatimes.com/world/south-asia/sri-lanka-to-reopen-all-schools-from-march-29/articleshow/81683666.cms (accessed Mar. 05, 2022).
[151] “New health guidelines to be followed till 31st May issued (English),” *NewsWire*, Apr. 23, 2021. https://www.newswire.lk/2021/04/23/new-health-guidee-to-be-followed-till-31st-may-issued-english/ (accessed Jan. 23, 2022).
[152] “All schools will be closed until April 30, says Sri Lanka’s Education Minister,” *ANI News*. https://www.aninews.in/news/world/asia/all-schools-will-be-closed-until-april-30-says-sri-lankas-education-minister20210427211125/ (accessed Mar. 05, 2022).

[153] "India's neighbours close borders over virus rampage," *France 24*, May 06, 2021. https://www.france24.com/en/live-news/20210506-india-s-neighbours-close-borders-over-virus-rampage (accessed Jan. 23, 2022).
[154] "Sri Lanka imposes inter-provincial travel restrictions to combat Covid-19," *mint*, May 10, 2021. https://www.livemint.com/news/world/sri-lanka-imposes-inter-provincial-travel-restrictions-to-combat-covid19-11620666026924.html (accessed Jan. 23, 2022).
[155] "The Latest: Sri Lanka halts trains, buses to curb virus," *AP NEWS*, May 21, 2021. https://apnews.com/article/india-coronavirus-vaccine-coronavirus-pandemic-lifestyle-travel-27b05e57b38dac7cc774428d059e82b7 (accessed Jan. 23, 2022).
[156] "Sri Lanka set for week-long lockdown as COVID-19 cases surge," *The New Indian Express*. https://www.newindianexpress.com/world/2021/may/24/sri-lankaset-for-week-long-lockdown-ascovid-19-cases-surge-2306955.html (accessed Jan. 23, 2022).
[157] "Revised New Health Guidelines Issued : Special restrictions for Western Province," *NewsWire*, Jun. 20, 2021. https://www.newswire.lk/2021/06/20/es-issued-more-restrictions-for-western-province/ (accessed Jan. 23, 2022).
[158] "Sri Lanka begins vaccinating 20-30 yrs age group - Times of India." https://timesofindia.indiatimes.com/world/south-asia/sri-lanka-begins-vaccinating-20-30-yrs-age-group/articleshow/85891330.cms (accessed Mar. 05, 2022).
[159] Ap, "Sri Lanka begins vaccinating 18-19 age group," *The Hindu*, Colombo:, Oct. 15, 2021. Accessed: Mar. 05, 2022. [Online]. Available: https://www.thehindu.com/news/international/sri-lanka-begins-vaccinating-18-19-age-group/article37001914.ece
[160] "Schools reopen across Sri Lanka even as teachers' strike continues," *The Hindu*, Colombo:, Oct. 21, 2021. Accessed: Mar. 05, 2022. [Online]. Available: https://www.thehindu.com/news/international/schools-reopen-across-sri-lanka-even-as-teachers-strike-continues/article37109624.ece
[161] "Sri Lanka imposes travel ban from six southern African countries over 'Omicron' Covid variant," *The New Indian Express*. https://www.newindianexpress.com/world/2021/nov/27/sri-lanka-imposes-travel-ban-from-six-southern-african-countries-over-omicron-covid-variant-2388818.html (accessed Mar. 05, 2022).
[162] "Sri Lanka : Sri Lanka lifts travel ban imposed on arrivals from Southern African countries." http://www.colombopage.com/archive_21B/Dec10_1639155824CH.php (accessed Mar. 05, 2022).
