# Supplementary material for: Comparative impact assessment of COVID-19 policy interventions in five South Asian countries using reported and estimated unreported death counts during 2020-2021
Source: PLOS Glob Public Health. 2023 Dec 27;3(12):e0002063. doi: 10.1371/journal.pgph.0002063 (PMC10752546; doi:10.1371/journal.pgph.0002063)
Supplement: Attachment — Submitted filename: Response to Reviewers.pdf [file pgph.0002063.s024.pdf]

SCHOOL OF PUBLIC HEALTH
BIOSTATISTICS
UNIVERSITY OF MICHIGAN

To
Dr. Catherine Kyobutungi and Dr. Madhukar Pai
Editors-in-Chief, Plos Global Public Health

Dear Dr. Kyobutungi and Dr. Pai,

Thank you for the opportunity to submit a revised version of the article titled "*Assessing Effects of Interventions on COVID-19 Mortality in South Asian Countries using Counterfactual-based Transmission Models*" (ID: PGPH-D-23-00912), co-authored with Dr. Jyotishka Datta, Dr. Debashree Ray, Dr. Swapnil Mishra, Dr. Rupam Bhattacharyya, Lauren Zimmermann and Dr. Bhramar Mukherjee. We have carefully revised the manuscript addressing each of the comments made by the reviewers. As a major change we have revised the introduction and the discussion sections to motivate the relevance of this work taking into account all the comments posed by the reviewers. Along with the response to the reviewers document, we have submitted both highlighted (in blue) and non-highlighted versions of the revised manuscript as per the journal revision guidelines.

Thank you for your consideration of our revised manuscript, and we believe that we have addressed all remaining concerns. We sincerely hope that the attached submission meets the standards of *PLoS Global Public Health* and hope for a positive evaluation.

Sincerely,
Ritoban Kundu, MStat
PhD Student,
Department of Biostatistics,
University of Michigan.

Reviewer 1

1. This is an analysis of COVID-19 mortality trends in South Asia for the period 2021 in relation to policies and largely relies upon the IHME excess mortality analysis. It does not strangely provide any data for the 2020 period where much of the virus spread and stringent policies were implemented, and the argument that this was not done given lack of data from Sri Lanka, also does not wash.

Response : We thank the reviewer for this comment. Following your suggestion, we have added the analysis of 2020 data using both reported and total estimated deaths (still using the IHME model) in the revised version.

Variability in COVID-19-related government interventions, lockdowns and vaccination initiatives across the five countries:

In 2020, India imposed four phases of nationwide lockdowns starting from March 25 to May 31 with dynamically adaptive measures to contain the spread of the virus (*vide* Supplementary Table S5). Starting from June 1, 2020 Indian government started to relax the restrictions with the six different unlock periods. Bangladesh observed a nationwide lockdown on similar time periods (March 26 - May 27). In contrast, countrywide lockdown measures were imposed for a short span of time in Pakistan (April 1 - May 9) compared to India or Bangladesh. Nepal experienced the longest period of lockdown which contributed towards lower COVID-19 mortality counts in the first half of 2020 (Figure 1 of Main Text). Moreover, each country differed in terms of varying restrictions imposed during their respective lockdown period.

Variability in observed pandemic-related metrics across countries

For 2020, Supplementary Table S1 and Supplementary Figure S1 show that the mean daily R_t for all the 5 countries were close to 1 and comparably similar at the start of the time period of analysis. The mean Case Fatality Rate (CFR) for Sri Lanka was lower than the other countries, while the test positive rates (TPR) for India and Pakistan were less than that of the other 3 countries, with Nepal and Bangladesh having the highest mean TPR of 0.13. From Table R1 (Table 2 of Main Text) and Figure R1 (Figure 1 of Main Text), we observe that India experienced the highest number of reported deaths per million (77) in 2020 compared to the other countries. In contrast Nepal has the highest number of estimated total (reported and unreported) deaths per million (763) as observed from Table R2 (Table 3 of Main Text) and Figure R1. The estimated total deaths per million for India (725) and Pakistan (708) are comparable. We observe a decline in terms of both reported and total deaths due to COVID for India starting November to the end of 2020. Supplementary Figure S5 shows the percent change from baseline for all the types of mobilities other than Residential is high for Pakistan. In the four categories other than Grocery-Pharmacy and Residential, the percent changes in mobility were moderate for India and for Nepal, in all of the cases, it was either on the lower or moderate side.

For Sri Lanka other than Residency, there was a similar trend of rise till September, 2020 and then there was a decline.

We exclude Sri Lanka from the 2020 counterfactual analysis due to the small number of deaths (both reported and total) and cases in 2020 as observed from Figure R1 (Figure 1 of Main Text).

Reported Deaths Analysis

From Table R1 (Table 2 of the main paper) and Figure 2, we observe that in terms of our counterfactual analysis based on **just reported deaths** India could have averted a substantial number of fatalities in all counterfactual situations where India is the recipient except the scenario Nepal → India. In terms of the counterfactual scenarios Bangladesh → Pakistan (CrI Averted 5 [-168,34]) and Pakistan → Bangladesh (CrI Averted -1 [-218,34]), we observe that both these countries are comparable in terms of pandemic performance. In contrast Nepal could have averted a considerable number of deaths in counterfactual scenarios with Bangladesh and Pakistan as donors (95% $CrI_{Bangladesh \rightarrow Nepal}$ 38 [-110,52] and 95% $CrI_{Pakistan \rightarrow Nepal}$ 34 [-155,52]). Overall in terms of reported mortality in 2020, Pakistan and Bangladesh were ahead in terms of pandemic control followed by Nepal and India.

Total Estimated Deaths Analysis

From Table R2 (Table 3 of Main Text) in terms of **estimated total (reported and unreported) deaths**, we observe that Nepal could have averted a substantial number of total deaths per million (at least 761 [712,763]) in all the three counterfactual scenarios with Nepal as the recipient. In terms of total deaths Bangladesh has the lowest estimated total deaths per million (509) compared to the other three countries. The pandemic performances of Bangladesh, Pakistan and India are comparable in terms of counterfactual scenarios. We observe that the relations are not symmetric. For example, the counterfactual scenarios (95% $CrI_{India \rightarrow Pakistan}$ -712 [-1639,-201] and 95% $CrI_{Pakistan \rightarrow India}$ -151 [-1103,361]) substantiate the inference that both nations would have incurred losses had they implemented the policy of the other country, indicating the uncertainty in the resultant estimates. In the context of total mortality in 2020, Bangladesh, Pakistan, and India demonstrate similar trends in pandemic control, while Nepal appears to fare the worst.

2020 reported deaths counterfactual table

Donor \ Recipient	India	Bangladesh	Pakistan	Nepal
**India**	77	Counterfactual : 147 [21,528]<br>Actual : 38<br>Averted : -109 [-490,17]	Counterfactual : 137 [17,486]<br>Actual : 37<br>Averted : -100 [-449,20]	Counterfactual : 74 [3,440]<br>Actual : 53<br>Averted : -21 [-387,50]
**Bangladesh**	Counterfactual : 12 [1,89]<br>Actual : 77<br>Averted : 65 [-12,76]	38	Counterfactual : 32 [5,205]<br>Actual : 37<br>Averted : 5 [-168,34]	Counterfactual : 15 [0,162]<br>Actual : 53<br>Averted : 38 [-110,52]
**Pakistan**	Counterfactual : 13 [1,101]<br>Actual : 77<br>Averted : 64 [-24,75]	Counterfactual : 39 [3,256]<br>Actual : 38<br>Averted : -1 [-218,34]	37	Counterfactual : 19 [0,209]<br>Actual : 53<br>Averted : 34 [-155,52]
**Nepal**	Counterfactual : 78 [4,540]<br>Actual : 77<br>Averted : -1 [-463, 72]	Counterfactual : 139 [10,740]<br>Actual : 38<br>Averted : -101 [-701,28]	Counterfactual : 135 [9,708]<br>Actual : 37<br>Averted : -98 [-670,28]	53

Table R1: Reported cumulative deaths per million with the 95% CrI on December 31, 2020 calculated starting from March 15, 2020 in different counterfactual situations. The rows correspond to the countries which are the donors, while the columns correspond to the recipients. The diagonal cells denote the reported deaths per million in the 5 countries. The off diagonal cells correspond to the counterfactuals, which consists of 3 quantities: Counterfactual Deaths Per Million, Actual Deaths per million and difference of deaths between the actual and counterfactuals that could have been averted.

2020 estimated total (reported + unreported) deaths counterfactual table

Donor \ Recipient	India	Bangladesh	Pakistan	Nepal
**India**	725	Counterfactual : 869 [437,7886]<br>Actual : 509<br>Averted : -360 [-7377,72]	Counterfactual : 1420 [909,2347]<br>Actual : 708<br>Averted : -712 [-1639,-201]	Counterfactual : 2 [0,51]<br>Actual : 763<br>Averted : 761 [712,763]
**Bangladesh**	Counterfactual : 583 [13,1350]<br>Actual : 725<br>Averted : 142 [-625,712]	509	Counterfactual : 857 [89,1388]<br>Actual : 708<br>Averted : -149 [-680,619]	Counterfactual : 1 [0,11]<br>Actual : 763<br>Averted : 762 [752,763]
**Pakistan**	Counterfactual : 877 [365,1829]<br>Actual : 725<br>Averted : -151 [-1103,361]	Counterfactual : 444 [242,10542]<br>Actual : 509<br>Averted : 65 [-10033,267]	708	Counterfactual : 1 [0,34]<br>Actual : 763<br>Averted : 762 [729,763]
**Nepal**	Counterfactual : 10360 [281,31973]<br>Actual : 725<br>Averted : -9635 [-31247, 444]	Counterfactual : 9201 [1303,34934]<br>Actual : 509<br>Averted : -8692 [-34425,-794]	Counterfactual : 10267 [931,30463]<br>Actual : 708<br>Averted : -9559 [-29755,-222]	763

Table R2: Total cumulative deaths (Reported and Unreported) with the 95% CrI on December 31, 2020 calculated starting from March 15, 2020 in different counterfactual situations. The rows correspond to the countries which are the donors, while the columns correspond to the recipients. The diagonal cells denote the estimated actual deaths per million in the 4 countries. The off diagonal cells correspond to the counterfactuals, which consists of 3 quantities: Counterfactual Deaths Per Million, Actual Deaths per million, and difference of deaths between the actual and counterfactuals that could have been averted. A cell is coloured red or blue depending upon whether the 95% CrI of the number deaths averted in a counterfactual situation lies entirely in the negative or positive side.

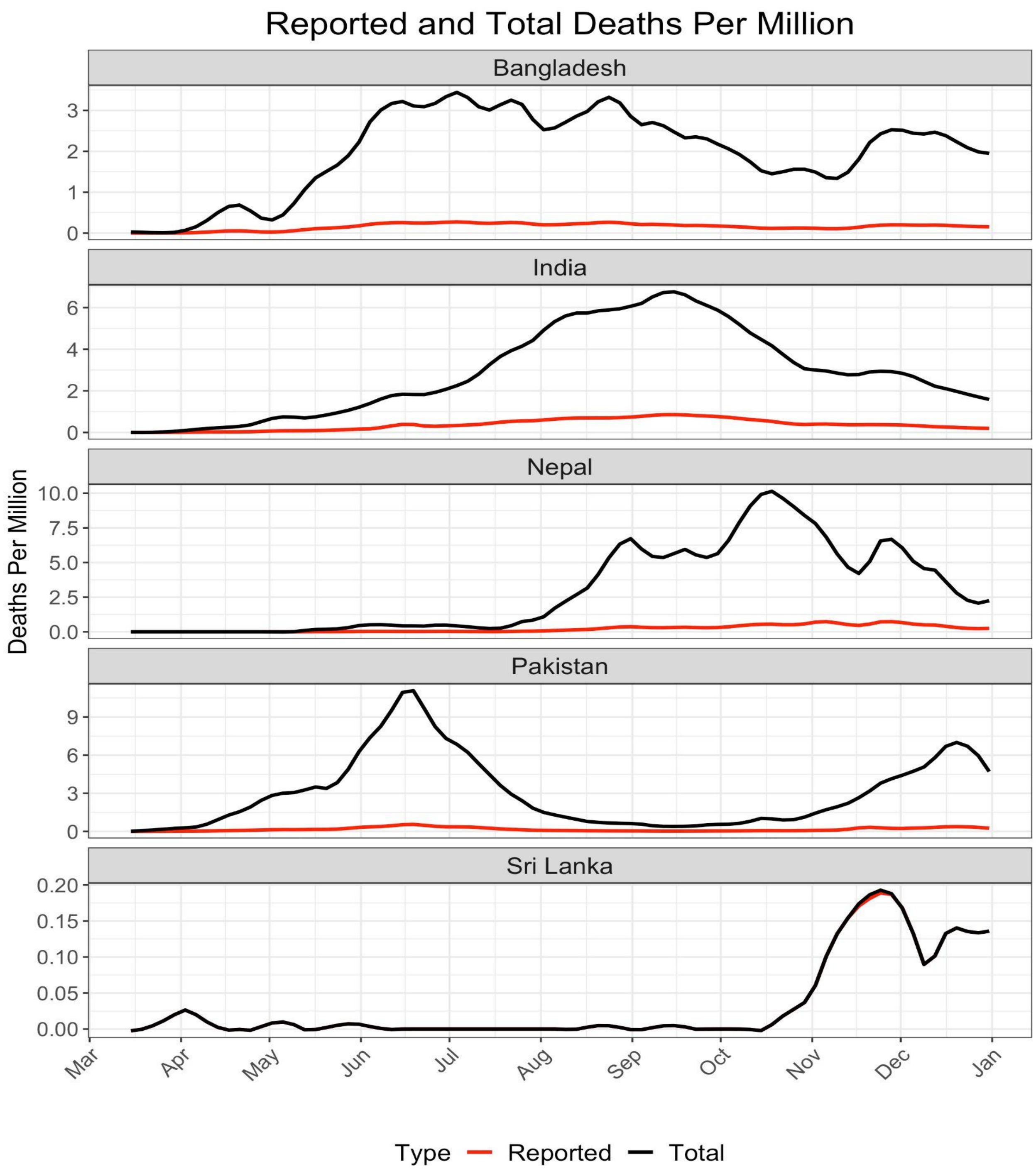

Figure R1: Daily Reported and Total (Reported + Unreported) Deaths Per Million in 2020 (March 15 2020 - Dec 31 2020) for each country. The red and the black lines correspond to the daily reported and total estimated death trajectories respectively.

2. My major concern is that the paper does not present any counter arguments as to why the IHME estimates might have been erroneous especially as they did not take into account several local studies assessing mortality estimates from graveyard surveillance and existing DHIS systems. While some impact on mortality and reduction in health system coverage in 2020 continuing to 2021 is undisputed (UNICEF ROSA South Asia study 2021), and recent analysis by Subu Subramaniam from the NFHS-5 rounds in India suggests a small impact on neonatal mortality, a counter analysis using local data on COVID-19 related deaths from India, Pakistan, Nepal and Bangladesh would have greatly strengthened the arguments.

Response: Thank you for bringing this up. To address this, we have incorporated the points you raised about the IHME estimates into our discussion as well as included a table (Table R3 below same as Supplementary Table S4) presenting a comparison of the disparities in excess mortality estimates along with 95% Confidence Interval using the three most prominent global COVID-19 mortality estimation studies: IHME, WHO, and The Economist for both 2020 and 2021. One critique directed at IHME is around the apparent implausibility of their estimates for certain countries such as Japan or Italy. Namely, the estimates for these two countries suggest that, without COVID-19, average expected mortality rates would have declined, which contradicts historical trends. Another noteworthy criticism of IHME pertains to its significant overestimations of deaths for certain regions, particularly sub-Saharan countries like Kenya and European countries like Denmark, when compared to the WHO estimates.

Table R3 (Supplementary Table S4) reveals that the IHME's combined estimate of excess mortality for India in 2020 and 2021 is the lowest among all three methods. Moreover, both the Economist and IHME estimates for these five countries demonstrate similarity when compared to WHO estimates. This gives us more confidence in going forward with the IHME predictions. We have included this rationale in the new discussion.

It is also crucial to emphasize that the primary focus of the analysis presented in our paper is on the ***relative ordering of the five countries*** in terms of pandemic trajectory performance rather than the *absolute magnitude of the actual death counts*. In terms of combined excess mortality in 2020 and 2021 together, the IHME's relative ordering (from highest to lowest excess deaths per million attributed to COVID-19) is: Nepal > Pakistan > India > Bangladesh > Sri Lanka. In contrast, the Economist's relative ordering is India > Bangladesh > Nepal >Pakistan >Sri Lanka. Lastly, the WHO's relative ordering is India > Nepal > Pakistan > Bangladesh > Sri Lanka. The apparent discordance between the rankings (*i.e.*, that none of the three methods agree with each other in terms of the relative ordering of the five countries regarding excess mortality estimates) shows the general lack of consensus and potential difference disparities in their assessments. Consequently, we agree that it is essential for researchers and policymakers to exercise caution and be mindful of the limitations and uncertainties when utilizing estimates from any of these three methods, not just limited to IHME.

Study	Year	India	Bangladesh	Pakistan	Nepal	Sri Lanka
Institute for Health Metrics and Evaluation (IHME)	2020	844 [661,1054]	579 [402,793]	889 [581,1219]	865 [627,1147]	0 [0,0]
	2021	1732 [1345,2163]	1583 [1095,2159]	1744 [1125,2383]	2741 [1987,3637]	694 [694,694]
	Total	2576 [2006,3217]	2162 [1497,2952]	2633 [1706,3602]	3606 [2614,4784]	694 [694,694]
World Health Organization (WHO) & Knutson (2022)	2020	609 [282,966]	281 [-96,681]	432 [-166,1070]	274 [-154,747]	-803 [-947,-649]
	2021	2861 [2141,3778]	579 [399,772]	632 [367,903]	842 [585,1108]	-158 [-395,948]
	Total	3470 [2423,4744]	860 [303,1453]	1064 [201,1973]	1116 [431,1855]	-408 [-1105,299]
The Economist & Solstad (2021)	2020	850 [393,1449]	821 [298,1280]	712 [381,1437]	680 [178,1236]	-115 [-484,227]
	2021	2284 [1247,3141]	1563 [928,2300]	1404 [979,2303]	1680 [1175,2570]	1176 [998,2006]
	Total	3133 [1640, 4590]	2384 [1222,3587]	2116 [1360,3740]	2360 [1353,3806]	1061 [514,2233]

Table R3: Total mortality estimates per million (reported and unreported) in 2020, 2021 and combined total till 2021 by IHME, WHO and the Economist for the five countries of interest. References : [1-3]

3. What about the virus spread and development of immunity at population level in 2020 and early 2021, even prior to widespread immunization strategies. There are cogent arguments that besides different population pyramids, both innate immunity and sub-clinical infections might have played a role in lower levels of COVID mortality in South Asia (see BMJ 2021 Jun 25;373:n1544. doi: 10.1136/bmj.n1544.).

Response : We thank the reviewer for this insight. We have included this important point and the reference paper in the discussion section. Our analysis is a retrospective evaluation of the pandemic control of the five different countries. It is important to acknowledge that we compared and generated the counterfactual scenarios by interchanging the time-varying reproduction number (R_t) series for each country. In this framework, the R_t time-series serves as a representative measure of the COVID pandemic for a country as a whole, encompassing the effects of innate immunity and other factors unique to each country. Incorporating R_t in our analysis adds robustness and depth to our findings, as it considers the complex interplay of

various elements influencing the pandemic's trajectory in each country. On the other hand, it loses the specificity of incorporating particular reasons that may alter transmission and lead to change in (R_t) . We would also like to point the readers to the analysis by *Quick et al.* (4) who adopted a similar viewpoint but posed a model for log (R_t) as a function of covariates. This type of hybrid models will need careful consideration for unpacking the specific causes that change transmission such as vaccination, seroprevalence and other healthcare related metrics.

It is essential to acknowledge that no model or approach is free from limitations, and we encourage further accumulation of data, research methods and discussion on refining causal methodologies and accounting for additional factors to enhance the accuracy and reliability of our findings.

4. The impact of vaccination is considered as a uniform variable whereas vastly different vaccines were used and coverage rates in many countries remained low (and mainly consisted of Chinese vaccines). How was vaccine efficacy factored in? What about several population based serological studies indicating high population level immunity by end 2020/early 2021. Were these

Response : We thank the reviewer for this important comment. In Supplementary Tables S1 and S2, we have included vaccination rates per million just as a metric of comparison across the five countries. We did not use vaccination proportion or count as a variable or introduce a new compartment in our mechanistic model. Similar to our response to Question 3, we compared and generated the counterfactual scenarios by interchanging the time-varying reproduction number (R_t) series for each country. This approach takes into account the varying effects of vaccinations and many other factors through the summary R_t series, thus indirectly considering the influence of vaccination efforts on the pandemic dynamics along with other factors in each scenario. It is worth noting that similar studies, such as the one conducted by *Salvatore et al.* (5), have employed R_t to generate counterfactual situations.

In addition, in our study we derived the prior parameters for infection fatality rates by utilizing the estimates obtained from serial serological surveys conducted in India. A comprehensive description of this integration can be found in the Methods section and Supplementary section S1. Notably, there exist other COVID models that employ different methodologies for incorporating serosurvey estimates. For instance, some models directly integrate these estimates into the R_t modeling, while others treat serosurvey data or percent immune at time *t* as a distinct compartment within the model, as demonstrated in the studies by *Quick et al.* (4) and *Zhou et al.* (6), respectively. By implementing our indirect approach and providing references to relevant studies, we contribute to the advancement of knowledge in the field of COVID-19 modeling and underscore the importance of methodological diversity and work that remains to be done in achieving a comprehensive understanding of the pandemic's dynamics.

5. Finally and perhaps most importantly, the policy analysis appears to be largely based on reported events or decisions, as opposed to implementation and is way past the most stringent periods of control (in 2020). By mid 2021 in many places, especially rural populations, it was largely game over for major COVID measures beyond masking. The excess mortality effect of the impact of economic downturn in the region, isn't really discussed in the context of mitigation measures and if they were effective or otherwise.

Response: We thank the reviewer for raising this important point. We have now included brief segments in the discussion section of the main manuscript that addresses the economic impact of the increase in the excess deaths attributed to COVID-19. In particular, we reference the study by *Zimmermann et al*., (7) that found that, by performing a value of a statistical life (VSL) calculation, the total COVID-19 deaths may be contextualized as an economic cost of approximately 30% of the annual GDP in India. We reproduce the relevant segment from the discussion section below for your convenience:

"Moreover, the lives and corresponding livelihoods lost from COVID-19 bore a compounding cost on the economy. *Zimmermann et al*., (7) found that this cost amounted to at least 30% (based on reported and unreported COVID-19 deaths) of India's GDP, when applying the value of a statistical life estimate by *Majumder & Madheswaran* (8) to the meta-analyzed estimates (7)."

6. In summary, though laudable, this effort falls short of the level of objective scrutiny any modeling exercise would undergo, and does not sufficiently take into account counterfactuals or arguments that the IHME excess mortality estimates might be gross over-estimates.

Response : We thank the reviewer for this comment. We understand and appreciate the critiques of our work that we have tried to address in the revised version. Please refer to our response to Question 1 along with Table R3 which provides a comparison of the excess mortality estimates from three highly cited sources: The Economist, WHO and IHME.

While we appreciate your critique for general overestimation of worldwide excess mortality by IHME, Table R3 (Supplementary Table S4) shows that Economist estimates of excess mortality till 2021 attributed to COVID-19 for India, Bangladesh, and Sri Lanka are higher than those of IHME. We acknowledge that while the IHME excess death estimate for India is conservative compared to the WHO, the IHME estimates for the other countries exceeds that of WHO. We have included sentences in the discussion noting the strengths and weaknesses of these three global excess deaths studies, as well as Table R3 for comparison. An uniform ordering is not possible across the methods, for countries across the world.

Assuming similarity in excess deaths estimation methodology across countries (*i.e.*, estimates from the same IHME global study), allows for a comparison of pandemic control efforts and policies among the nations under study. As such, in the relative scale of analysis, the excess

mortality rankings from IHME can still provide valuable insights and be of some reliability, despite the limitations of the IHME estimates.

Reviewer 2

Quantifying and assessing the impact of interventions on COVID-19 mortality is important to inform the public health management and could help for such other directly transmitted infectious diseases. While measures of mortality are usually estimated retrospectively, the real-time changes in the health capacity, settings and interventions (including effective contact tracing) could impact both the denominator and numerators of the measures. Therefore, such study should be carefully adopted for the implementation further.

Authors have used their existing framework to analyze the data from different locations of some low and middle income countries in south Asia. The main concern is the rationale of the study is not clearly presented in the introduction with potential hypotheses, this is not merely the same analysis under their reported framework for UK and European countries. Though several such studies on assessing the direct and indirect impact of COVID-19 related interventions found to be significant for COVID-19 infection, transmissibility, severity and mortality. I found this study interesting for the art of linking the measures of transmissibility to mortality for assessing impact of these interventions on COVID-19 mortality, but has several scopes of improvements as stated below.

Question 1. As mentioned above, authors have used their existing framework to analyze the data from other locations, I found the introduction should be improved by including the rationale of this study to take up clearly.
- I see authors have mentioned the challenges and limitations of the public health setting and data access for these locations and tried to illustrate by providing a table 1 in the introduction itself but the text is not in order to follow for general readers.
- Further several parts should not be in the introduction, "… A detailed description of the different interventions implemented by the five South Asian countries of interest in 2021 are provided in Supplementary Table Interventions. Upon observation of the five countries under scrutiny, it is noteworthy that the commencement of their respective vaccination programs occurred in close temporal proximity. Specifically, the initiation of the vaccination campaigns in these countries took place approximately between the late January and early February in the year of 2021. With the exception of India, all four countries undertook comprehensive nationwide lockdowns for multiple days during the initial half of 2021. There were disparities in the length and commencement of these lockdowns across the countries. Conversely, India implemented region-specific lockdowns within individual states as opposed to a unified lockdown throughout the country in 2021. There existed notable heterogeneity in the restrictions imposed on various facets such as schools, country borders, as well as domestic and international flights across the different countries. …". This section should be in the data section and some parts may be in discussion. I wonder if the authors are using these results and data to

establish their hypothesis, that should be clearly mentioned in the text and presented accordingly. In that case, I'd suggest for presenting the evidence to establish the hypothesis, authors could present some summary statistics with the references instead of using Tables directly, which are any way based on secondary data.

Response : We thank the reviewer for the overall positive reception of the paper and appreciate the constructive suggestions. We have reorganized the entire manuscript by restructuring the introduction section and, in particular, we have moved the segment on pharmaceutical and non-pharmaceutical interventions to a later more appropriate section, as suggested. We would also like to clarify that the comparative metrics on Table 1 of Main Text are not used in establishing or forming our hypotheses about transmission rates and lives saved, they do not feature as covariates in our Bayesian mechanistic model. Their sole purpose is to establish the contextual differences among the South Asian LMICs that are not directly captured by our model and to situate our findings in the pre-pandemic socio-economic and demographic indicators for these LMICs. Many of the interventions and the pandemic control measures were documented on local government and municipal websites and are hard to scrape from the web now. In that sense our paper also serves an archiving purpose of pandemic control measures across the five countries through Supplementary Tables S5 and S6.

We also thank the reviewer for recognizing that the current analysis is not "merely the same analysis under their reported framework for UK and European countries", and we have highlighted and emphasized this point further in our revised introduction (see below). The use of estimated total deaths instead of reported deaths is indeed a new feature of the paper. We have reproduced the revised introduction below for your convenience:

"COVID-19 created a polycrisis all over the world and the impact has been felt everywhere, with almost no country spared. Low and middle-income countries (LMICs) initially faced a steep challenge in providing medical services with a fragile health infrastructure (9) and scarce social safety-nets. However, during the course of the pandemic, the overall performances of the LMICs in responding to the global crisis improved, and some LMICs, like Vietnam (10), were able to effectively contain the spread of virus through early and aggressive public health measures such as testing, tracing and isolating cases. In this article, we focus on South Asian LMICs, *viz.*, India, Bangladesh, Pakistan, Sri Lanka and Nepal through a counterfactual lens of their relative mortality burdens via unreported deaths. Our study is motivated by three key factors. Firstly, as *Younus (2021)* (11) commented, the pandemic created a severe public health and economic crises pushing millions of households into poverty and substantially increasing income and wealth inequalities. Despite the huge literature on the impact of Covid-19 on these countries, there is essentially no prior work on comparing their relative performances that could provide a basis for policy comparison and build the ground for future prevention plans.

Secondly, with hybrid immunity against the virus acquired via the cumulative effect of natural and vaccine immunity, LMICs like India faced a less severe Omicron compared to the previous two waves (12). For example, Babu *et al.* (13) analyzed the pandemic responses of eight South Asian countries and found that these countries had lower mortality rates from COVID-19 than

many other countries worldwide, making counterfactual comparisons between UK and European countries (14) less informative in the context of SA LMICs. Finally, as we discuss below, while there are pronounced similarities in certain aspects of their social determinants of health, existing comorbidities and the demographic indicators of the five countries (*vide* Table 1 of Main Text), there are also marked variations in these key indicators, and this duality underscores the importance of the relative comparison of pandemic performance while recognizing the contextual differences. We would also like to note here that the comparison of these pre-pandemic metrics only serve as a rationale for this study and not used as evidence or input in our methodology.

A key challenge in modeling pandemic trajectory in LMICs is the significantly lower data resolution and quality compared to high-income countries, as highlighted by prior research (15). This disparity in data quality contributes to the challenge of under-reporting of deaths in LMICs, thereby complicating policy evaluation in these countries (7). As such, assessing data quality and developing reliable estimates of total infections and fatalities are crucial prerequisites for modeling and conducting counterfactual evaluations in LMICs. It is recommended that total mortality estimates be used in these evaluations to address issues of selection bias and under-reporting (16,17). Any fair comparison across LMIC countries should consider death underreporting instead of relying on the reported number of deaths. Several such estimates for excess deaths during the pandemic are now available (18,1,2,3).

Mishra et al. (14) explored using a semi-mechanistic Bayesian model how timing and effectiveness of control measures in the UK, Sweden and Denmark shaped COVID-19 mortality in each country using a counterfactual assessment: *what would the impact have been, had each country adopted the others' policies?* Their results show that small changes in the timing or effectiveness of interventions have disproportionately large effects on reported mortality due to COVID-19 pandemic. We extend the work of *Mishra et al.* (14) by including an in-depth evaluation of five LMICs from South Asia, namely India, Bangladesh, Pakistan, Sri Lanka and Nepal in 2020-2021. We choose to focus on these five countries as they are home to 23.5% of the population of the world and more importantly, there exists no prior across-country *`what if?'* analysis for these LMICs. Furthermore, the critical issue of under-reporting of deaths was accounted for in our analysis by using the IHME (The Institute for Health Metrics and Evaluation) excess mortality estimates. We compare and contrast the counterfactual results from both reported and estimated total mortality. The analysis of total estimated deaths using a counterfactual framework is a novel contribution of this paper. This has been a first attempt to compare and contrast the five South Asian countries of interest in terms of both reported and estimated total deaths.

Although we cannot comprehensively account for all the differences within and between countries, our analysis provides a starting point for performing counterfactual analysis in the LMIC context. This is further substantiated by *Salvatore et al.* (5), who consider similar analysis but only for India generating counterfactuals for policy interventions, but no work on pairwise counterfactuals across LMICs are available till date. These countries under consideration are geographically close to each other and share common social determinants of health (SDOH),

such as, poverty, inequality, rapid urbanization, unhygienic living conditions, strong child immunization programs, healthcare accessibility issues, and challenges with disease surveillance and public health systems (19,20). Our approach using total estimated deaths tries to place all countries on the same playing field and control for differences in reporting COVID deaths.

To set the backdrop, a pre-pandemic summary of these countries' socio-economic and demographic relevant factors for COVID-19 is presented in Table 1 of Main Text. The details are discussed in the Results Section. Despite the variations in GDP, comorbidities, and age distributions presented in Table 1, there are similarities in culture, geography, poverty levels, and healthcare quality among the five countries of interest, making a quasi-experimental counterfactual assessment of their COVID-19 situations valuable from a *global public health* perspective. In terms of reported COVID-19 deaths, about 70% of the deaths from 2020 till 2021 occurred in 2021 for these five South Asian countries of interest. Our main focus of interest in this study is assessing the COVID-19 pandemic situation in 2021 in terms of reported and estimated total deaths. We also consider the 2020 data separately as some of the policy measures were more centrally coordinated and stringent in 2020, the variant and the immunity landscape was different from 2021. A detailed description of the different interventions implemented by the five South Asian countries of interest in 2020 and 2021 are provided in Supplementary Table S5 and S6 respectively. The summary synthesis of these tables are presented in the Results Section. This table serves as an important archive of the policy interventions implemented by these countries since several of the sources of information are not publicly available anymore. The measures undertaken by the different governments were contingent upon the distinct circumstances, socio-political influences and health infrastructure in each country, leading to an ensemble of divergent approaches in managing the pandemic situation. Hence, a comprehensive counterfactual analysis to assess the impact of the policy measures undertaken in these five countries is of high significance.“

One important note is that the information presented in Table 1 of Main Text and Supplementary Tables S5 and S6 are solely for the purpose of setting a background of the disparities of the five countries of interest. None of this information is used for modeling purposes. Please also refer to the responses to Questions 3 and 4 of Reviewer 1 and Questions 4 and 7 of Reviewer 2.

Question 2. The supplementary tables are not numbered, rather presented with the ‘table title’ in the text, I am not sure it is the requirement of the journal. In general, we numbered them as supplementary tables and cited them in the main text as required.

Response: We thank the reviewer for this correction. We have numbered each supplementary Table and Figure. For example, the Supplementary Table on Interventions is now labeled as Supplementary Table S5.

3. The data description is not clear for each level of data and countries. I suggest authors could mention country-wise data diversity and may consider some text from introduction to the data section in case.

Response : We thank the reviewer for this suggestion. We inserted the following Table (Table R4) in the supplementary material as Table S3 which comprises information on data sources of different data types used in our analysis. We included two paragraphs at the beginning of the results section and removed the suggested paragraph from the introduction.

**Data Types**	**Data Sources**	**Link**
Reported Mortality Data	Johns Hopkins Coronavirus Resource Center	https://coronavirus.jhu.edu/data
Excess Mortality Estimates (IHME)	The Institute of Health Metrics and Evaluation	covid19.healthdata.org/
Mobility Data	Google Mobility Data	google.com/covid19/mobility/

Table R4 : Different data sources used in our analysis along with the website link.

Variability in socio-economic and demographic factors relevant for COVID-19 across the five countries.

The prevalence of comorbidities associated with COVID-19 varied significantly across the five south Asian countries. A pre-pandemic summary of their socio-economic and demographic factors relevant for COVID-19 is presented in Table 1 of the main text. Sri Lanka had the highest percentage of the population with asthma (15.8%) and India the lowest (3.9%). The percentage of people with diabetes ranges from 9.7% in Sri Lanka to 16.4% in Nepal. However, some commonalities exist among all or subsets of these countries. For instance, India had only 0.71 hospital beds per million, while the others had around 0.10, which made the high number of severe symptomatic COVID-19 cases a significant concern for these countries. Furthermore, in Pakistan and Nepal, a high proportion of the population comprised young people aged 0-14 years, with percentages of 35.7% and 34.9%, respectively. India, Bangladesh, and Nepal have similar proportions of elderly people (65+), around 5.3%. Despite the variations in GDP, comorbidities, and age distributions, there are similarities in culture, geography, poverty levels, and healthcare quality among the five countries of interest, making a quasi-experimental counterfactual assessment of their COVID-19 situations valuable from a global public health perspective.

Variability in COVID-19-related government interventions, lockdowns and vaccination initiatives across the five countries.

2020 Description: In 2020, India imposed four phases of nationwide lockdowns starting from March 25 to May 31 with dynamically adaptive measures to contain the spread of the virus (Supplementary Table S5). Starting from June 1, 2020 Indian government started to relax the

restrictions with the six different unlock periods. Bangladesh observed a nationwide lockdown on similar time periods (March 26 - May 27). In contrast, countrywide lockdown measures were imposed for a short span of time in Pakistan (April 1 - May 9) compared to India or Bangladesh. Nepal experienced the longest period of lockdown which contributed towards lower COVID-19 mortality counts in the first half of 2020 (Figure 1 of Main Text). Moreover each country differed in terms of varying restrictions imposed during their respective lockdown period.

2021 Description : The initiation of the vaccination campaigns in these countries took place approximately between the late January and early February in the year of 2021 (Supplementary Table S6). With the exception of India, all four countries undertook comprehensive nationwide lockdowns for multiple days during the initial half of 2021. There were disparities in the length and commencement of these lockdowns across the countries. Conversely, India implemented region-specific lockdowns within individual states as opposed to a unified lockdown throughout the country in 2021. There existed notable heterogeneity in the restrictions imposed on various facets such as closing of schools, sealing country borders, as well as limiting domestic and international flights across the different countries."

4. Authors opted the mobility baselines, calculated as the median value for the corresponding day of the week during January 3, 2020 - February 6, 2020. What is the rationale to consider such base line? Better to mention it clearly in the text with evidence of supports. How this baseline for one month could represent the other timing of the year? By January 2020, the human mobility behavior already started changing even though the Governments had not implemented any specific intervention and travel restrictions. Authors could consider the baseline for each week of a year from the data during earlier years. I have not seen any sensitivity analysis on these baselines over the counterfactual results and hence the final outcomes.

Response : We thank the reviewer for this inquiry. In our analysis, we incorporated Google mobility data as a comparative measure to study five South Asian countries of interest and to explore potential reasons for sudden surges in the time-varying reproduction number series. Google mobility data is based on a baseline established during a specific period (Jan 3–Feb 6, 2020) and represents changes in mobility for each country relative to this baseline (see the Google COVID-19 Community Mobility Reports").

As this granular mobility data was released by Google to aid in mitigating the impact of COVID-19, there is no pre-2020 information available at such a detailed level for these countries. This is the sole reason behind the choice of the baseline period. Consequently, the changes in mobility with respect to the fixed baseline offer insights into human mobility trends throughout the course of the COVID-19 pandemic in 2020 and 2021.

It is important to note that we did not employ mobility trends as variables in our transmission models. Instead, we used them to provide a sense of variation across different countries. By placing the transmission model results in the context of societal (Table 1 of Main Text) and

policy variation (Supplementary Tables S5 and S6), we aimed to provide a more comprehensive and nuanced understanding of the factors that influence pandemic dynamics and ultimately lead to fluctuations in the reproduction number.

5. In equations 1 and 2, authors used the IFR for reported and total retrospectively and assumed to be constant. While the expected deaths d_t,m was temporally evaluated by introducing the temporal components as daily infection and their density to death from the time of infections as the backward looking.
- First how density (Pi) could be constant across the epidemics, in fact several factors could change the distribution over time? If authors assumed this constant should be clearly mentioned in the text and discuss in the limitation section.

Response: We thank the reviewer for this astute observation. At first we introduce the required renewal equations relevant to this question. Let $d_{t,m}$ denote the expected number of reported daily deaths attributed to COVID-19, where *t* denotes the day or time and *m* denotes the country. The relation between $d_{t,m}$ with the past number of daily infections $c_{\tau,m}$ (reported + unreported) is

$$d_{t,m} = \mathrm{IFR}_{\mathrm{Rep}} \sum_{\tau=0}^{t-1} c_{\tau,m}\pi_{t-\tau}, \qquad \textbf{(R1)}$$

$$\mathrm{IFR}_{\mathrm{Rep}} = \frac{\text{Cumulative number of deaths reported}}{\text{Cumulative number of infections}}$$

Where π is the infection to death distribution. On the other hand let $d^*_{t,m}$ be the expected number of total (reported + unreported) daily death estimates on a given day *t* for country *m*. The modified renewal for total mortality is

$$d^*_{t,m} = \mathrm{IFR}_{\mathrm{Tot}} \sum_{\tau=0}^{t-1} c_{\tau,m}\pi_{t-\tau}, \qquad \textbf{(R2)}$$

$$\mathrm{IFR}_{\mathrm{Tot}} = \frac{\text{Cumulative number of deaths (reported + unreported)}}{\text{Cumulative number of infections}}.$$

We used two different prior distributions for both IFR_{rep} and IFR_{tot} in 2020 and 2021 respectively. We have clarified the distributions of the IFRs in detail in the Methods Section and Supplementary Section S1. Previously we only specified the distributions of IFR that we used in our 2021 analysis and now we have added the same for 2020.

The IFRs in India in 2020 and 2021 were derived based on the serial serosurvey estimates in *Zimmermann et al. (7)*. Based on these serosurvey estimates for India and using the

assumption that the ratio of IFRs are same as that of CFR, we obtained the estimates of mean and standard deviation of the distributions of IFRs in other countries that did not have serial serosurveys. The details of these assumptions and calculations are provided in the Supplementary Section S1. Specifically in 2020 we assumed the prior distribution of IFR_{rep} and IFR_{tot} follows normal distributions with (mean, sd) as (0.05,0.03) and (0.37,0.21) respectively.

In contrast in 2021 we assumed the prior distribution of IFR_{rep} and IFR_{tot} follows normal distributions with (mean, sd) as (0.08,0.05) and (0.39,0.23) respectively. However, within a given year of analysis, we adopted a fixed prior for Infection Fatality Rate (IFR) to ensure the identifiability of the parameters in equations R1 and R2. We include this issue as a limitation of our analysis in the Discussion section. The serosurvey estimates in India were roughly at a six months gap. During the period of 2020 and 2021, only 4 serosurveys were conducted in India and we incorporated those results to obtain the prior parameters on the IFR. Note that our study specifies prior distributions for the IFRs and subsequently derives posterior estimates of transmission model parameters by incorporating data and prior knowledge. This approach enables us to combine existing information and empirical evidence with the data at hand, yielding more informed and robust shrinkage estimates of the IFR for the selected countries.

In our study, we modeled the infection-to-death distribution (π) as the sum of two independent distributions: the incubation period (time from infection to the onset of symptoms) and the time between the onset of symptoms and deaths. To capture variations in the virus strain, we considered two different incubation period distributions based on the specific time period under investigation. For instance, in the year 2020, the mean incubation period of the ancestral COVID variant was set at 5 days based on existing literature. Consequently, we formulated the incubation period as a random variable following the Gamma distribution with shape and scale parameters of 5.8 and 0.94, respectively (21).

In contrast, in 2021 with delta strain as the major variant of COVID, we used an incubation period a distribution with mean 4 days [95 CI (3,5), https://wwwnc.cdc.gov/eid/article/29/4/22-0854_article]. Therefore the distributions of the incubation period differ across 2020 and 2021 in our analysis. We used the same onset to death distribution (Gamma distribution with shape and scale parameters 1.45 and 10.43 respectively (14)) .

- Authors have rightly considered the unobserved cases in the formulation, but I could not find how they have estimated the unobserved cases over time and not clear in the text. Please clarify or provide the details.

Response : We thank the reviewer for this inquiry. In Equation 1, the latent variable $c_{t,m}$ symbolizes the count of COVID-19 infections for a given day denoted as '*t*' and a specific country indicated as '*m*,' encompassing both reported and unreported/covert cases. The

Bayesian analysis utilizes the COVID-19 death data as input, rather than directly utilizing reported cases or estimated infection counts.

We use two versions of this input death data, first use reported deaths and then use estimated total deaths as reported by IHME in Equations R1 and R2 respectively. The implicit information on infection counts come through the prior selection on the infection fatality rate or the IFR. The quantification of infections is then determined by the available data on observed and total estimated fatalities, as articulated through Equations R1 and R2 correspondingly.

For example, let us place our attention on Equation R1. In this equation, IFR_{rep} denotes the ratio characterizing observed deaths relative to the aggregate count of cases, encompassing both reported and unreported infections. The prior distribution applied to IFR_{rep} uses serosurveys conducted in India (see previous paragraph and supplementary methods section S1.
The following equation R3 link $c_{t,m}$ to past infection $c_{\tau,m}$ where $\tau \leq t$.

$$c_{t,m} = S_{t,m} R_{t,m} \sum_{\tau=0}^{t-1} c_{\tau,m} g_{t-\tau}$$

$$S_{t,m} = 1 - \frac{\sum_{i=1}^{t-1} c_{i,m}}{N_m} \qquad \textbf{(R3)}$$

Using Equation R3 and R1, the resulting posterior estimates are obtained for $c_{t,m}$. Analogously, we derive posterior estimates concerning total cases (encompassing both reported and unreported cases) by employing data related to the comprehensive estimation of total deaths, as outlined in Equation R2.

6. The selling part is the ‘linking Rt to the mortality’. I found this part is not clear enough. - Authors used the renewal process to estimate Rt from daily number of cases. Where generation time distribution is approximated with the serial interval distribution as g ~ Gamma (6.5, 0.62), but not cited the evidence for this parametric distribution. I wonder authors have evaluated from the line-list data for each country. Please clarify or revise the text with the relevant information.

Response : We thank the reviewer for the feedback and agree that we needed to improve our explanation of the model description. Please find below the answers to the specific questions.

We would like to clarify first that the renewal equation used in our work doesn’t use the daily cases to estimate the time varying reproduction number but rather uses the **daily deaths** to estimate it. This can be seen from equations R1-R3. In a nutshell our formulation uses infections (not just positive cases- this is basically true infections) as a latent variable and then

links infections to deaths via Equation R1 or R2. Then the input data in the model are daily deaths as illustrated in Equations R1 and R2. More details for this formulation can be found in (22–24). The source for our generation distribution is (22,25), where authors used the line-list data from China during the start of the pandemic. We would like to emphasize that we have not used the line-list data from countries of interest in the paper. The access to line-list data is not available to us. Moreover in these countries there was absence of any effective contact-tracing to really estimate the generation distribution from the line-list data. We have added this in the paper now for clarity.

- Considering the Rt as random process with exponential form is very crude assumption, in fact, the Rt usually considered to follow gamma distribution, which surely an exponential family of distribution but should not be an extreme member of the family. I suggest to provide the evidence for this assumption and cite the original study for this.

Response : We would like to clarify here that we have not assumed any parametric form for the time varying reproduction number (R_t). In our formulation, exponentiating the random walk process essentially serves the purpose of restricting the latent random process to be strictly positive which is different from assuming the R_t to have an exponential distribution. For a more nuanced discussion of this approach we encourage the reviewer to refer to the detailed discussion provided in (22,24).

- The measure Rt is mostly driven by the susceptibility, but while reconstruction via. random process, the authors estimated e^α0,m, which is R0,m the basic reproduction number for country m. Then multiplied a quantity e^lϵw(t),m, which fixed to 1 at the start of the epidemic and then follow the random walk. I am not sure how it will ensure to have decreasing trend accounting the depletion of susceptible in the population.

Response : We are sorry for the confusion here. However, we would like to clarify that our model accounts for the depletion in the susceptible population explicitly as given by Equation 5 (Equation R3) in the main text and with more details about it below Equation 10 (Equation R3) in the Supplementary Section S1. We have now added text to the main text explicitly stating that Equation R3 accounts for the population effect (depleting susceptible population).

- Finally, the linking of transmissibility to the mortality is not found in the text, I can't assess this part as it not available in their original article clearly. Please provide the details how these Rt could be translated to the mortality in temporal scale.

Response : We agree with the reviewer that we have not explicitly emphasized this link in our text. The main reason for this was that the information was already provided in prior work (*22,*14). As we have stated in response to the first question above, equations R1-R2 in the main text show how we link deaths to latent (true) infections, and then equation R3 link R_t to latent infections. We have emphasized the process in the main text while describing our methods again.

7. How the pharmaceutical intervention (vaccination) was used to model along with non-pharmaceutical interventions, the text is not clear to locate the formulation. Please provide the details in the main text itself. Even I couldn't find then in the supplementary except the data.

Response : We thank the reviewer for this inquiry. Please refer to our response to Q4 of reviewer 1. In summary, we did not use a specific compartment for vaccination/immune faction in the population.We have tried to clarify that we only use the daily mortality data in the Bayesian mechanistic model and assume a prior structure on the IFR.

8. Accordingly, the result section should be revised, I don't know why the result section's title is "Results in 2021"? I found the discussion part is poorly written with no proper literatures' links and comparison. Several recent publications were not discussed in context. The discussion ended up with no data driven conclusion.

Response: We thank the reviewer for the critique. We changed the title of the results section to "Results". We additionally divided the results into two parts : "Results for 2021" and "Results for 2020" as suggested by Reviewer 1.

We thank the reviewer for the suggestion of improving the discussion section. We have structured the discussion section with thematic sub-headings. Following are the segments of the discussion section with major changes:

Why is this work important?

In this paper, we focus on comparing the effect of PHIs on reported and estimated total deaths via a counterfactual assessment framework for five south Asian countries, namely, India, Bangladesh, Pakistan, Sri Lanka, and Nepal in 2020-2021, with the main focus on 2021. While geographically proximal, these countries differ significantly in terms of economic, cultural, religious factors, age pyramid and most importantly, population-level comorbidities and healthcare infrastructure like number of hospital beds as observed from Table 1 of the main text (45). The considerable similarities and differences in these determinants of pandemic outcomes motivate a comparison of the five countries in terms of their policy implementation and efficacy.

Using a renewal process-based Bayesian transmission model from Mishra et al. (2021) (14), we adopted a counterfactual-based approach where one country could hypothetically experience the transmission patterns of the pandemic quantified by time-varying reproduction numbers of another country. Using this R_t transfer schema, we analyzed the consequences of shifting policies across the five countries of interest for the two halves of 2021 separately and for 2020. We considered a relative approach for constructing the counterfactuals to preserve certain

inherent features of the recipient countries while artificially and hypothetically modifying the policy interventions. Since relatively small changes in R_t can lead to huge variations in the number of cases and deaths, it is not reasonable to compare the countries in terms of their time-varying reproduction numbers only, and we focused on comparing the number of deaths in different countries in different counterfactual situations. To this end, the counterfactuals offer an improved common ground to compare the mortality situations over absolute comparisons of the death time series.

Since data capture and coverage of death registration systems differ across the five countries, using the estimated total deaths as opposed to reported deaths add to the novelty of this work.

What are the major findings and key takeaways?
In 2020 in terms of reported deaths, except for Nepal, in all the other counterfactual scenarios where India is the recipient, India could have averted a significant number of deaths. Overall, our study in 2020 in terms of reported mortality, highlights that Bangladesh and Pakistan made slightly better decisions than the other countries, while Nepal performed the worst in terms of pandemic evaluation. Sri Lanka had a negligible number of reported deaths in 2020. In context of total estimated (reported and unreported) mortality in 2020, Bangladesh, Pakistan, and India demonstrate similar trends in pandemic control, while Nepal appears to fare the worst. Therefore the pandemic performance of India in terms of total deaths is better compared to reported deaths in 2020.

On the other hand, our analyses for the reported deaths in 2021 indicate that Pakistan and Nepal undertook the overall best measures on the whole of 2021 followed by Bangladesh. India lagged slightly in the first half of 2021, but starting from July 2021, it recovered substantially, whereas Sri Lanka performed the worst among the five countries through 2021. The counterfactual analyses for total deaths indicate that Pakistan and India led throughout, followed by Bangladesh, while Nepal lagged behind. Like in case of the reported deaths, Sri Lanka was behind the other countries in terms of total deaths too. Evaluation of the under reporting factor for cases for all the five countries at the end of 2021, using the results from the model fitting and the serosurveys in India shed further light on the data quality differences across the countries.

What are the major limitations of this work?
The quality of input reported deaths: As mentioned in the section Introduction, the five countries of interest faced a lot of challenges in regard to the quality of the reported deaths data. The major possible factors owing to this challenge are high population density, limited testing capacity, variation in reporting across different regions, government transparency and inadequate death registration. These factors contribute towards high under reporting of mortality due to COVID-19 (26-30).

The quality of input excess death estimates: First, the reliability of COVID-19 Excess Mortality estimates, in particular, the individual country-specific estimates have been questioned by several authors (31-35) who caution us against their use in policy evaluations. As a comparative tool we include a Table R3 (Supplementary Table S4) presenting the disparities in

excess mortality estimates along with 95% Confidence Interval using the three most prominent and comprehensive global COVID-19 mortality estimation studies: IHME, WHO, and The Economist for both 2020 and 2021.

One critique directed at IHME is around the apparent implausibility of their estimates for certain countries such as Japan or Italy. Namely, the estimates for these two countries suggest that, without COVID-19, average expected mortality rates would have declined, which contradicts historical trends. Another noteworthy criticism of IHME pertains to its significant overestimations of deaths for certain regions, particularly sub-Saharan countries like Kenya and European countries like Denmark, when compared to the WHO estimates. Table R3 (Supplementary Table S4) reveals that the IHME's combined estimate of excess mortality for India in 2020 and 2021 is the lowest among all three methods. Moreover, both the Economist and IHME estimates for these five countries demonstrate similarity when compared to WHO estimates. This gives us more confidence in going forward with the IHME predictions.

It is also crucial to emphasize that the primary focus of the analysis presented in our paper is on the ***relative ordering of the five countries*** in terms of pandemic trajectory performance rather than the *absolute magnitude of the actual death counts*. In terms of combined excess mortality in 2020 and 2021, the IHME's relative ordering (from highest to lowest excess deaths per million attributed to COVID-19) is: Nepal > Pakistan > India > Bangladesh > Sri Lanka. In contrast, the Economist's relative ordering is India > Bangladesh > Nepal >Pakistan >Sri Lanka. Lastly, the WHO's relative ordering is India > Nepal > Pakistan > Bangladesh > Sri Lanka. The apparent discordance between the rankings (i.e., that none of the three methods agree with each other in terms of the relative ordering of the five countries regarding excess mortality estimates) shows the general lack of consensus and potential difference disparities in their assessments. Consequently, we agree that it is essential for researchers and policymakers to exercise caution and be mindful of the limitations and uncertainties when utilizing estimates from any of these three methods, not just limited to IHME.

The choice of prior on IFRs: Second, while there is existing knowledge on the infection fatality rates for India using the serosurveys, for the other countries, the IFRs are calculated using an additional assumption: the ratio of case fatality rates is the same as the ratio of infection fatality rates. Although this is not a completely invalid assumption to make, more granular information on these metrics from the other countries would have been of help. Moreover due to unavailability of the serosurvey estimates in India on a regular basis, we adopted a fixed prior for Infection Fatality Rate (IFR) to ensure the identifiability of the parameter estimation in our models within a year of analysis (different for 2020 and 2021).

Consideration of Lives versus Livelihoods:

Moreover, the lives and corresponding livelihoods lost from COVID-19 bore a compounding cost on the economy. *Zimmermann et al.* (7) found that this cost amounted to at least 30% (based on reported and unreported COVID-19 deaths) of India’s GDP, when applying the value of a

statistical life estimate by *Majumder & Madheswaran,* (8) to the meta-analyzed mortality estimates. *Karunathilaka* (2021) (36) studies the positive and negative impacts of COVID-19 with reference to challenges on the supply chain in South Asian countries. *Deyshappriya* (2020) (37) reveals that there was a greater potential of increasing poverty incidence related to all poverty types under COVID-19 pandemic situation in Sri Lanka. *Khalid et al.* (2020) (38) studies the effects of COVID-19 in Pakistan from a sociological and economic perspective.

Finally, one major limitation of the fundamental premise of this work is that it may be entirely infeasible to impose the restrictions mandated by one country to another. For a diverse country like India, it is almost impossible to impose an uniform set of rules and regulations all over the country, which has been pointed out in recent literature (39). The National lockdown in 2020 on 1.4 billion people will remain historic and possibly unprecedented. A more sensible way of swapping the intervention scenarios between countries would be to map spatial (administrative units) and temporal (pandemic timeline and waves) on a case-by-case basis to ensure improved calibration of the real scenarios as opposed to a fictional national level counterfactual setting we considered.

What are some open problems that merit future investigation?

Incorporating uncertainty in excess death estimates: Using estimated total deaths as input in the model does not account for the huge uncertainty in these estimates. There is no way to validate the model against ground truth as unreported deaths are and will remain unobservable. A way to mitigate this would be to construct synthetic pandemic timelines based on known generative epidemiological models and construct and evaluate the counterfactuals based on the synthetic data. We leave these options for future exploration in a more integrated hierarchical framework.

Modifying the approach for generation of counterfactuals: We generated the counterfactual scenarios by interchanging the time-varying reproduction number (R_t) series for each country. This framework assumes that R_t serves as a representative measure of the COVID pandemic for a country as a whole, capturing the effects of innate immunity and other factors unique to each country. Incorporating this R_t transfer schema in our analysis implicitly assumes that the complex interplay of various factors that influence the pandemic's trajectory in each country is summed into this one metric . Other studies such as the one conducted by *Salvatore et al.* (5) have also employed R_t to generate counterfactual situations. However, generating counterfactuals in a meaningful way is a potential vast area of research.

There are alternative ways to model the differences in countries based on varying factors like innate immunity, subclinical infections, vaccinations, population based serological surveys, patterns of mobility and several others. For example, there are cogent arguments that besides different population pyramids, both innate immunity and sub-clinical infections might have played a role in lower levels of COVID mortality in South Asia (40). *Shyamsunder et al.* (41)

studies the impact of vaccination on COVID-19 outbreaks by categorizing infected people into non-vaccinated, first dose-vaccinated, and second dose-vaccinated groups and exploring the transmission dynamics of the disease outbreaks. There exist other COVID models that directly integrate serosurvey estimates into the R_t modeling, while others treat serosurvey data as a distinct compartment within the model, as demonstrated in the studies by *Quick et al.* (4) and *Zhou et al.* (6), respectively. There have been recent developments in implementing synthetic control methods for evaluating the effectiveness of different interventions on the spread of COVID 19 contagion (42-44).

In summary, despite the limitations in the formulation of methods adopted in this study, it is important to recognize the data quality differences while evaluating policies in the global south. With better data and transparent methods we can arrive at less biased comparisons across countries in terms of their pandemic performance. Naively comparing the reported number of total deaths at a national level across countries, without normalizing by age structure, population size and differences in death reporting, the ultimate objective of data-driven policy making will always remain elusive to a policymaker.

9. I suggest to revise the title for general readers, no need to mention 'counterfactual-based' terms in the title itself. In fact, it is not 'counterfactual-based' transmission model rather the transmission models were used to analyzed for counterfactual scenarios. Further the terms 'Assessing Effects' can be revised as 'Impact assessment'.

Response: We thank the reviewer for this suggestion. We propose a new title for our paper:

"Comparative impact assessment of COVID-19 policy interventions in five South Asian countries using reported and estimated unreported death counts during 2020-2021"

Bibliography

1. COVID-19 Projections. *Inst. Health Metr. Eval. IHME*, (available at https://covid19.healthdata.org/).
2. Knutson V, Aleshin-Guendel S, Karlinsky A, Msemburi W, Wakefield J. Estimating Global and Country-Specific Excess Mortality During the COVID-19 Pandemic. arXiv; 2022 May. Report No.: arXiv:2205.09081. doi:10.48550/arXiv.2205.0908
3. The Economist, Solstad SU. The pandemic's true death toll. The Economist. 2021. (github link and page link)
4. Quick, Corbin, Rounak Dey, and Xihong Lin. "Regression models for understanding COVID-19 epidemic dynamics with incomplete data." Journal of the American Statistical Association 116.536 (2021): 1561-1577.
5. Salvatore, Maxwell, et al. ``Lessons from SARS-CoV-2 in India: A data-driven framework for pandemic resilience." \textit{Science Advances} 8.24 (2022): eabp8621
6. Zhou, Yiwang, et al. "A spatiotemporal epidemiological prediction model to inform county-level COVID-19 risk in the United States." (2020).
7. Zimmermann, L., Bhattacharya, S., Purkayastha, S., Kundu, R., Bhaduri, R., Ghosh, P., \& Mukherjee, B. (2021). SARS-CoV-2 infection fatality rates in India: systematic review, meta-analysis and model-based estimation. \textit{Studies in Microeconomics}, 9(2), 137-179.

8. Majumder, Agamoni, and S. Madheswaran. Value of statistical life in India: A hedonic wage approach. Institute for Social and Economic Change, 2018.
9. Owain David Williams, Ka Chun Yung \& Karen A. Grépin (2021) The failure of private health services: COVID-19 induced crises in low- and middle-income country (LMIC) health systems, \textit{Global Public Health}, 16:8-9, 1320-1333, DOI: 10.1080/17441692.2021.1874470
10. Tran, Bach Xuan, et al. ``Reaching further by Village Health Collaborators: The informal health taskforce of Vietnam for COVID-19 responses." \textit{Journal of global health} 10.1 (2020).
11. Younus, Uzair. ``The Impact of COVID-19 on South Asian Economies." (2021).
12. Bhattacharya, Manojit, et al. ``Hybrid immunity against COVID-19 in different countries with a special emphasis on the Indian scenario during the Omicron period." \textit{International Immunopharmacology} 108 (2022): 108766.
13. Babu, G. R., Khetrapal, S., John, D. A., Deepa, R., \& Narayan, K. V. (2021). Pandemic preparedness and response to COVID-19 in South Asian countries. \textit{International Journal of Infectious Diseases}, 104, 169-174.
14. Mishra, S., Scott, J.A., Laydon, D.J. et al. Comparing the responses of the UK, Sweden and Denmark to COVID-19 using counterfactual modelling. \textit{Sci Rep} 11, 16342 (2021).
15. Sethy, P. The myth of South Asian Exceptionalism (2021). https://www.thinkglobalhealth.org/article/myth-south-asian-exceptionalism
16. Bhaduri, R., Kundu, R., Purkayastha, S., Kleinsasser, M., Beesley, L. J., Mukherjee, B., \& Datta, J. (2022). Extending the susceptible‐exposed‐infected‐removed (SEIR) model to handle the false negative rate and symptom‐based administration of COVID‐19 diagnostic tests: SEIR‐fansy. \textit{Statistics in Medicine}, 41(13), 2317-2337.
17. Datta, J., \& Mukherjee, B. (2021). Discussion on ``Regression Models for Understanding COVID-19 Epidemic Dynamics With Incomplete Data". \textit{Journal of the American Statistical Association}, 116(536), 1583-1586.
18. Wang, Haidong, et al. ``Estimating excess mortality due to the COVID-19 pandemic: a systematic analysis of COVID-19-related mortality, 2020–21." \textit{The Lancet} 399.10334 (2022): 1513-1536.
19. Bishwajit, Ghose, Seydou Ide, and Sharmistha Ghosh. ``Social determinants of infectious diseases in South Asia." \textit{International Scholarly Research Notices} 2014 (2014).
20. Zaidi, Anita KM, Shally Awasthi, and H. Janaka deSilva. ``Burden of infectious diseases in South Asia." \textit{Bmj} 328.7443 (2004): 811-815
21. Lauer, Stephen A., et al. "The incubation period of coronavirus disease 2019 (COVID-19) from publicly reported confirmed cases: estimation and application." *Annals of internal medicine* 172.9 (2020): 577-582.
22. Flaxman S, Mishra S, Gandy A, Unwin HJT, Mellan TA, Coupland H, et al. Estimating the effects of non-pharmaceutical interventions on COVID-19 in Europe. Nature. 2020;584: 257–261.
23. Sharma M, Mindermann S, Rogers-Smith C, Leech G, Snodin B, Ahuja J, et al. Understanding the effectiveness of government interventions against the resurgence of COVID-19 in Europe. Nat Commun. 2021;12: 5820.
24. Bhatt S, Ferguson N, Flaxman S, Gandy A, Mishra S, Scott JA. Semi-Mechanistic Bayesian modeling of COVID-19 with Renewal Processes. J R Stat Soc Ser A Stat Soc. 2023; qnad030.
25. Verity R, Okell LC, Dorigatti I, Winskill P, Whittaker C, Imai N, et al. Estimates of the severity of coronavirus disease 2019: a model-based analysis. Lancet Infect Dis. 2020;20: 669–677.
26. Biswas, Raaj Kishore, Awan Afiaz, and Samin Huq. "Underreporting COVID-19: the curious case of the Indian subcontinent." *Epidemiology & Infection* 148 (2020): e207.
27. Hanifi, Syed Manzoor Ahmed, et al. "Insights into excess mortality during the first months of the COVID-19 pandemic from a rural, demographic surveillance site in Bangladesh." *Frontiers in Public Health* 9 (2021): 622379.
28. Rahaman, Khan Rubayet, Md Sultan Mahmud, and Bishawjit Mallick. "Challenges of testing COVID-19 cases in Bangladesh." *International Journal of Environmental Research and Public Health* 17.18 (2020): 6439.
29. Khalid, Atiqa, and Sana Ali. "COVID-19 and its Challenges for the Healthcare System in Pakistan." *Asian bioethics review* 12.4 (2020): 551-564.
30. Giri, Anil K., and Divya RSJB Rana. "Charting the challenges behind the testing of COVID-19 in developing countries: Nepal as a case study." *Biosafety and Health* 2.02 (2020): 53-56.
31. O'Neill D. Conflicting COVID-19 excess mortality estimates. \textit{Lancet}. 2023 Feb 11;401(10375):431.

32. Moeti, Matshidiso, et al. Conflicting COVID-19 excess mortality estimates. \textit{The Lancet} 401.10375 (2023): 431.
33. Bager, Peter, et al. ``Conflicting COVID-19 excess mortality estimates." \textit{The Lancet} 401.10375 (2023): 432-433.
34. Donzelli A. Conflicting COVID-19 excess mortality estimates. \textit{Lancet.} 2023 Feb 11;401(10375):432.
35. Schöley J, Karlinsky A, Kobak D, Tallack C. Conflicting COVID-19 excess mortality estimates. \textit{Lancet.} 2023 Feb 11;401(10375):431-432.
36. Karunathilake, Kankanige. "Positive and negative impacts of COVID-19, an analysis with special reference to challenges on the supply chain in South Asian countries." Journal of social and economic development 23.Suppl 3 (2021): 568-581.
37. Deyshappriya, N. P. "Economic impacts of COVID-19 macro and microeconomics evidences from Sri Lanka." Economic Impacts of COVID-19 Macro and Microeconomics Evidences from Sri Lanka (May 10, 2020) (2020).
38. Khalid, Atiqa, and Sana Ali. "COVID-19 and its Challenges for the Healthcare System in Pakistan." Asian bioethics review 12.4 (2020): 551-564.
39. Bhagat, Ram B., \textit{et al}. ``The COVID-19, migration and livelihood in India: challenges and policy issues." \textit{Migration Letters} 17.5 (2020): 705-718.
40. Bhutta, Zulfiqar A., et al. "Beyond the numbers: understanding the diversity of covid-19 epidemiology and response in South Asia." bmj 373 (2021).
41. Bhatter, S., et al. "A new fractional mathematical model to study the impact of vaccination on COVID-19 outbreaks." Decision Analytics Journal 6 (2023): 100156.
42. Alfano, Vincenzo, Salvatore Ercolano, and Lorenzo Cicatiello. "School openings and the COVID-19 outbreak in Italy. A provincial-level analysis using the synthetic control method." *Health Policy* 125.9 (2021): 1200-1207.
43. Mills, Melinda C., and Tobias Rüttenauer. "The effect of mandatory COVID-19 certificates on vaccine uptake: synthetic-control modelling of six countries." *The Lancet Public Health* 7.1 (2022): e15-e22.
44. Lim, Jue Tao, et al. "Estimating direct and spill-over impacts of political elections on COVID-19 transmission using synthetic control methods." *PLoS computational biology* 17.5 (2021): e1008959.
45. Chongsuvivatwong, Virasakdi, et al. "Health and health-care systems in southeast Asia: diversity and transitions." *The Lancet* 377.9763 (2011): 429-437.
