# Supplementary material for: Comparative impact assessment of COVID-19 policy interventions in five South Asian countries using reported and estimated unreported death counts during 2020-2021
Source: PLOS Glob Public Health. 2023 Dec 27;3(12):e0002063. doi: 10.1371/journal.pgph.0002063 (PMC10752546; doi:10.1371/journal.pgph.0002063)
Supplement: Attachment — Submitted filename: Response to Reviewers.pdf [file pgph.0002063.s025.pdf]

SCHOOL OF PUBLIC HEALTH
BIOSTATISTICS
UNIVERSITY OF MICHIGAN

To
Dr. Catherine Kyobutungi and Dr. Madhukar Pai
Editors-in-Chief, Plos Global Public Health

Dear Dr. Kyobutungi and Dr. Pai,

Thank you for giving us the opportunity to submit a re-revised version of the article titled "*Assessing Effects of Interventions on COVID-19 Mortality in South Asian Countries using Counterfactual-based Transmission Models*" (ID: PGPH-D-23-00912), co-authored with Dr. Jyotishka Datta, Dr. Debashree Ray, Dr. Swapnil Mishra, Dr. Rupam Bhattacharyya, Lauren Zimmermann and Dr. Bhramar Mukherjee.

In this second revision, we incorporated some major changes:

- We have rewritten the Methods section in full addressing each of the comments made by the second reviewer.
- We carried out a detailed sensitivity analysis with respect to prior specifications on Infection Fatality Ratio (IFR), generation time distribution and inclusion of mobility data into the stochastic model for the time varying reproduction number.

Along with the response to the reviewers document, we have submitted both highlighted (in blue) and non-highlighted versions of the re-revised manuscript as per the journal guidelines.

Thank you for your consideration of our re-revised manuscript. We hope that the revisions we made address the remaining concerns of the *PLoS Global Public Health* editorial panel and hope for a positive decision.

Sincerely,
Ritoban Kundu, MStat
PhD Student,
Department of Biostatistics,
University of Michigan.

**Response to Reviewers**

Before we proceed with the itemized response, we would like to address an overarching concern about the methods not being clear. Part of it was due to relegating the methods to the supplementary section. In the revised version, we have rewritten the Models subsection of the Methods section in the main text with more details on each component of the model and also included the following flowchart to provide an overview of the Bayesian semi-mechanistic model.

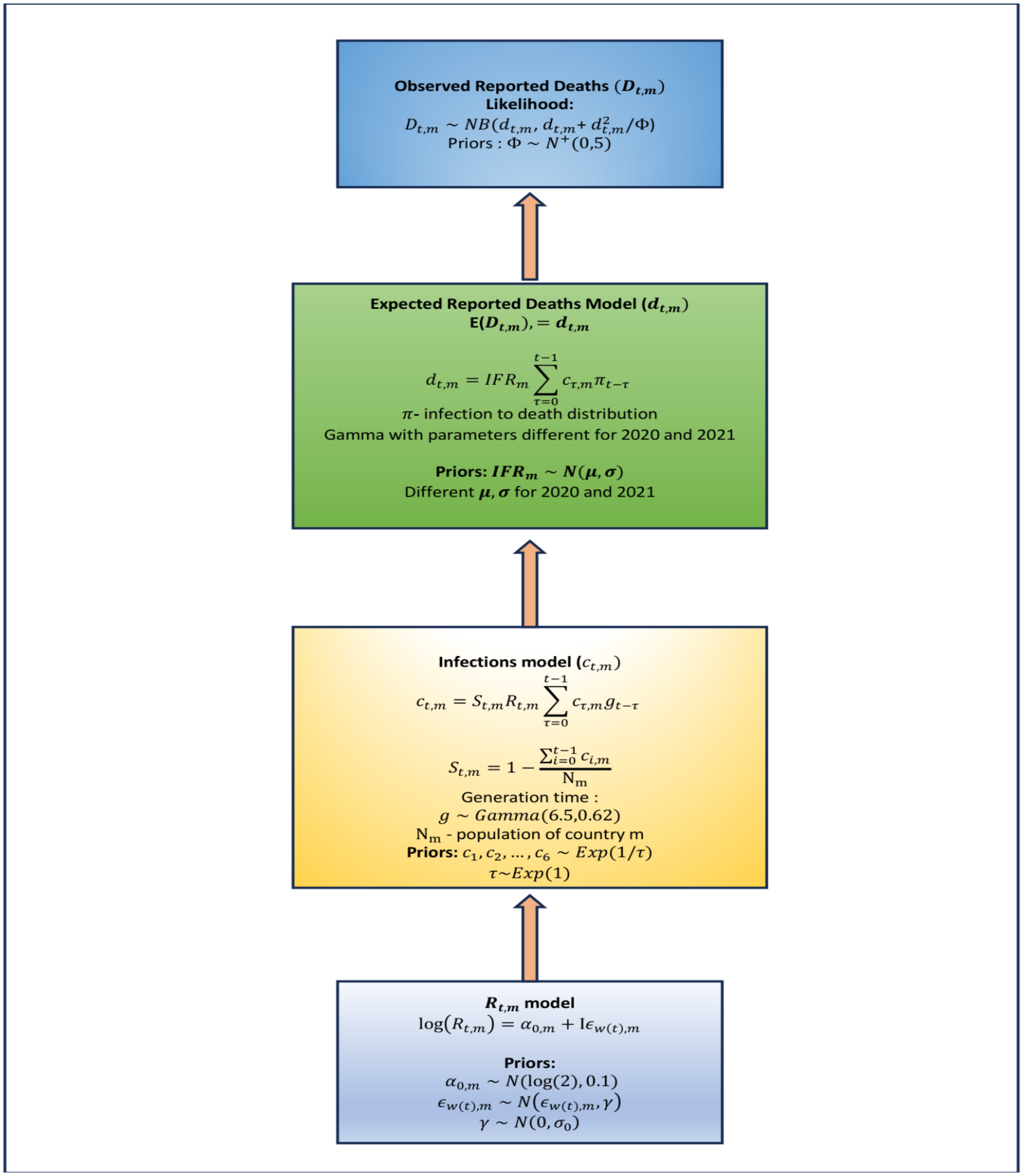

**Figure R1 : Schematic representation of the different components of the Bayesian Semi-Mechanistic Transmission Model.** Each box represents and provides details of the components that define the overall stochastic structure of the model.

**COMPONENTS OF THE BAYESIAN SEMI-MECHANISTIC MODEL**

We used a Bayesian semi-mechanistic renewal process model for both reported and estimated total deaths for all five countries. Figure R1 describes a summary of the specifications of the stochastic components of the model. One important point to note is that the renewal equations in our work **do not use** the daily reported cases as input data to estimate the model parameters but rather use the daily reported and total estimated deaths from IHME as the only input data. Infections (not reported cases) are inferred from the death data, the infection fatality rate (IFR) and the model for country specific time varying reproduction number using the renewal equation.

1. **Transmission Model :**

**Observed Data :**

$D_{t,m}$ - Reported Number of deaths attributed to COVID-19, where $t$ denotes the day or time and $m \in \{1, 2, 3, 4, 5\}$ denotes the country.

$D^{*}_{t,m}$ - Total Number of estimated deaths from IHME (sum of reported and unreported) attributed to COVID-19, where $t$ denotes the day or time and $m \in \{1, 2, 3, 4, 5\}$ denotes the country. However, one can use estimates from any source.

**Likelihood :**

Let $d_{t,m} = E(D_{t,m})$ denote the expected number of reported deaths. The distributional assumptions for $D_{t,m}$ used here to define the likelihood is given by

$$D_{t,m} \sim Negative\ Binomial\ (d_{t.m},\ d_{t.m} + d^{2}_{t,m}/\ \Phi), \qquad \Phi \sim N^{+}\ (0, 5) \qquad \textbf{(R1)}$$

Similarly for estimated total mortality analysis we defined $d^{*}_{t,m} = E(D^{*}_{t,m})$ to denote the expected number of total (reported and unreported) estimated deaths. The distributional assumptions for $D^{*}_{t,m}$ used here to define the likelihood is given by

$$D^{*}_{t,m} \sim Negative\ Binomial\ (d^{*}_{t.m},\ d^{*}_{t.m} + d^{*2}_{t,m}/\ \Phi), \qquad \Phi \sim N^{+}\ (0, 5) \qquad \textbf{(R2)}$$

where, $N^{+}\ (\mu, \sigma)$ is a half-normal distribution with mean μ and standard deviation σ.
$X \sim N^{+}\ (\mu, \sigma)$ if, $X \sim |Y|$ and $Y \sim N(\mu, \sigma)$ .

Note that one limitation is that we consider the point estimates $D^*_{t,m}$ obtained from IHME as the estimated total deaths and ignore the uncertainty associated.

2. **Renewal Equations :**

**Linking Mortality to Infections:**

The relation between the observed expected number of deaths, $d_{t.m}$ on a given day $t$ for the country $m$ with the past number of daily infections $c_{\tau,m}$ (observed + unobserved) is given by the discrete sum :

$$d_{t,m} = IFR_{Rep,m} \sum_{\tau=0}^{t-1} c_{\tau,m} \pi_{t-\tau} \qquad \textbf{(R3)}$$

where $IFR_{Rep,m}$ denotes the infection fatality rate for the reported deaths analysis defined as probability of a reported death given infection.

On the other hand, the relation between the observed expected number of total estimated deaths, $d^*_{t.m}$ on a given day $t$ for the country $m$ with the past true number of daily infections $c_{\tau,m}$ (observed + unobserved) is:

$$d^*_{t,m} = IFR_{Tot,m} \sum_{\tau=0}^{t-1} c_{\tau,m} \pi_{t-\tau} \qquad \textbf{(R4)}$$

where $IFR_{Tot,m}$ denotes the infection fatality rate for the total estimated deaths analysis defined as probability of total estimated death (reported and unreported) given infection.

Here $\pi$ is the discretized density of the infection to death distribution**.** Let us concentrate on equation (3) for the time being. The intuition behind equation R4 is similar. The expression $IFR_{Rep,m}. c_{\tau,m} \pi_{t-\tau}$ denotes the reported expected number of patients to die on day $t$ for the country $m$ who developed their infection on day $\tau$. Therefore $IFR_{Rep,m} \sum_{\tau=0}^{t-1} c_{\tau,m} \pi_{t-\tau}$ denotes the number of expected deaths on day $t$.

**Linking Current Infections to Past Infections Using Reproduction Number :**

**The true number of infected individuals is modeled using a discrete renewal process:**

The true number of infected individuals is related to the past infected counts using the following equation :

$$c_{t,m} = S_{t,m} R_{t,m} \sum_{\tau=0}^{t-1} c_{\tau,m} g_{t-\tau} \qquad \textbf{(R5)}$$

$$S_{t,m} = 1 - \sum_{i=0}^{t-1} c_{i,m} / N_m$$

where $g$ denotes the discretized version of the generation time density. The population size of country $m$ is specified by $N_m$. The infections at day $t$ depend on the number of infections in previous days weighted by the discretized generation time distribution. This weighting is then multiplied by the country specific time varying reproduction number $R_{t,m}$ which denotes the average number of secondary infections per infection at a given time. The adjustment factor $S_{t,m}$ accounts for the depletion of susceptibles in the population because even in absence of interventions herd immunity will reduce the number of daily infections through susceptible populations. One important point is that $R_{t,m}$ **is unadjusted for population depletion,** however the term $S_{t,m} R_{t,m}$ takes into account the adjusted reproduction number for population depletion. Without including the depletion factor into $R_{t,m}$ helps to considerably improve posterior topology and inference. However while generating the counterfactuals, the adjustment factor is always included to account for susceptible depletion.

### 3. Structure of the model for $R_{t,m}$

$R_{t,m}$ for day $t$ and country $m$ is defined as the country specific time varying reproduction number which models the average number of secondary infections per infection at a given time. In our main analysis, the unadjusted $R_{t,m}$ for day $t$ and country $m$ is formulated as a random process

$$log(R_{t,m}) = \alpha_{0,m} + I\epsilon_{w(t),m} \qquad \textbf{(R6)}$$

where the conversion from days to weeks is encoded in $w(t)$.

Therefore $R_{0,m} = e^{\alpha_{0,m}}$. Every 7 days, $w$ is incremented, i.e we set $w(t) = [(t - t^{start})/7] + 1$, where $t^{start}$ is the date of intervention. For all $t < t^{start}$ , $\epsilon_{w(t),m}$ is set to 0. For $t \geq t^{start}$, to specify the random walk process, we introduce the parameter $\gamma \sim N(0, \sigma_0)$. and then model $\epsilon$ as

$\epsilon_{w(t),m} \sim N(\epsilon_{w(t)-1,m}, \gamma)$. We model $log(R_{t,m})$ to ensure positivity of $R_{t,m}$. The indicator function is used to ensure that the random walk can only start at the date of intervention which is $t^{start}$.

## 4. Specification/choice of priors and other input parameters

### Prior Specification on the Infection Fatality Rate (IFR)

A major challenge exists in obtaining realistic values of the prior distribution parameters for IFR(s) in four of the countries we consider (Pakistan, Nepal, Sri Lanka and Bangladesh). These four countries lack reliable serosurveys or linelist (cases/deaths) data. We tackle this challenge by using the IFR estimates of India based on serosurveys and assuming that the ratio of IFRs between two countries is the same as that of Case Fatality Ratios (CFR) in those two countries. Case fatality Ratio (CFR) for a country at day $t$ is defined as the ratio of cumulative number of reported deaths and cumulative number of reported cases till day $t$. More formally, for any two countries $x$ and $y$, we assume that $IFR_x/IFR_y = CFR_x/CFR_y$ where $IFR$ can be both $IFR_{Rep}$ and $IFR_{Tot}$ .

The IFRs for India in 2020 and 2021 were derived based on the serial serosurvey estimates in *Zimmermann et al. (2021)* [1]. The CFR for all of the countries can be obtained using the JHU CSSE COVID database (https://coronavirus.jhu.edu/map.html). Based on these serosurvey estimates for India and using the assumption that the ratio of IFRs are same as that of CFR, we obtained the estimates of mean and standard deviation of the prior distributions of IFRs in other countries that did not have serial serosurveys.

Specifically, in 2020 we assumed the prior distributions of $IFR_{rep}$ and $IFR_{tot}$ (Infection fatality rates for reported and estimated total mortality analysis respectively) to be normal distributions with (mean, sd) as (0.05,0.03) and (0.37,0.21) respectively. In contrast, in 2021 we assumed the prior distribution of $IFR_{rep,m}$ and $IFR_{tot,m}$ follows normal distributions with (mean, sd) as (0.08,0.05) and (0.39,0.23) respectively. The means and standard deviations of the IFRs are chosen based on the obtained IFRs of all the five countries. However, within a given year of analysis, we adopted a fixed prior for Infection Fatality Rate (IFR) to ensure the identifiability of the parameters in equations R3 and R4.

### Infection to death distribution

In our study, we modeled the infection-to-death distribution (π) in equations R3 and R4 as the sum of two independent distributions: the incubation period (time from infection to the onset of symptoms) and the time between the onset of symptoms and deaths. To capture variations in the virus strain, we considered two different incubation period distributions based on the specific time period under investigation. For instance, in the year 2020, the mean incubation period of

the ancestral COVID variant was set at 5 days based on existing literature (*Lauer, Stephen A., et al. (2020)* [2]). Consequently, we formulated the incubation period as a random variable following the Gamma distribution with shape and scale parameters of 5.8 days and 0.94, respectively. We used the same onset to death distribution (Gamma distribution with shape and scale parameters 1.45 days and 10.43 respectively (*Mishra, S et al. (2021)* [3]) for 2020 and 2021. Therefore in 2020, the infection to death distribution is the sum of two independent Gamma variables given by,

$$\pi \sim Gamma(5.8, 0.9) + Gamma\,(1.45, 10.43)$$

In contrast, in 2021 with delta strain as the major variant of COVID, we used an incubation period with a distribution of mean 4 days [95 CI (3,5), *Zeng et al. (2023)* [4]. Therefore the distributions of the incubation period differ across 2020 and 2021 in our analysis. In 2021, the infection to death distribution is the sum of two independent Gamma variables given by,

$$\pi \sim Gamma(62.22, 0.06) + Gamma\,(1.45, 10.43)$$

The discrete probability mass function (pmf) $\pi_t$ for day $t$ was obtained using the expression

$\pi_t = \int_{t-0.5}^{t+0.5} \pi(\tau)\, d\tau$ for $t = 0, 1, 2, 3, ...$ .

### Generation Time Distribution

Generation time is defined as the time between when a person becomes infected and when they subsequently infect another person. Usually the generation time is unknown. Similar to *Mishra et al. (2021)* [3] ,we approximate the generation time distribution $g$ with density $g(\tau)$ by the serial interval distribution (time from symptom onset in one person to the time of symptom onset in the person they infect). We chose both the generation time and the serial interval to be Gamma distribution with mean 6.5 days and standard deviation 4.2 (*Bi, Q et al. (2020)* [5]), where authors used the line-list data from China during the start of the pandemic. We would like to emphasize that we have not used the line-list data from countries of interest in the paper. The access to line-list data is not available to us. Moreover in these countries there was absence of any effective contact tracing to really estimate the generation distribution from the line-list data. The generation time distribution is discretized by $g_t$ for day $t$ was obtained using the expression

$g_t = \int_{t-0.5}^{t+0.5} g(\tau)\, d\tau$ for $t = 2, 3, ..,$ and $g_1 = \int_{0}^{1.5} g(\tau) d\tau$.

### Priors on the parameters of the model for $R_{t,m}$ and choice of the seeding parameters

For 2020, we used $t^{start}$ to be March 15, 2020. On the other hand, $t^{start}$ for the 1st and 2nd halves of 2021 were given by January 1, 2021 and July 1, 2021. Since the basic reproduction number is given by $R_{0,m} = e^{\alpha_{0,m}}$ , the prior distribution of $\alpha_{0,m}$ was chosen to be $N(log(2),\ 0.1)$ which can represent a broad range of plausible mean basic reproduction numbers with support

from 1 to 3 (obtained by the EpiEstim package in R for the five countries of interest in 2020 and 2021). We seeded the model with six sequential days of an equal number of infections given by $c_{1,m} = c_{2,m} = ... = c_{6,m} \sim Exp(1/\tau)$. In Supplementary Section S1, we discuss in details the estimation of true number of infections $c_{t,m}$ and the choice of $\tau$ used. The transmission model parameters are estimated in the probabilistic programming language *Stan (Carpenter et al. (2017)* [6]*)* using an adaptive Hamiltonian Monte HMC sampler.

## 5. Constructing Counterfactual Scenarios

We follow the exact structure for creating the counterfactual model as detailed in *Mishra et al*. $R_{o,m}$ for country $m$ is defined as the average number of secondary infections produced by a typical case of an infection in a population where everyone is susceptible. In contrast, $R_{t,m}$ for day $t$ and country $m$ is defined as the country specific time varying reproduction number which models the average number of secondary infections per infection at a given time. Under this setting, a country's time-varying reproduction number ($R_{t,m}$) is replaced by the $R_{t,m}$ of a different country. Then, the new resulting $R_{t,m}$ is used to simulate the evolving epidemics for that country. For the remainder of the text, we refer to the country from which the intervention scenario is considered for constructing a counterfactual situation as the *donor*, and the country whose mortality data under the *donated* intervention is being analyzed as the *recipient*. We apply the *relative reductions to the donor countries* $R_{t,m}$ *starting from the intervention date* for generating the counterfactual. Denoting $x$ as the donor country and $y$ as the recipient country, the $R_{t,y}$ becomes $R_{0,y}/R_{0,x}.R_{t,x}$ after the intervention date. The relative approach ensures that the counterfactual decrease is proportional to the initial dynamics of the recipient country.

Mathematically, for two countries $x$ and $y$, the time varying reproduction number $R_t$ are modeled as $R_{t,x} = e^{\alpha_{0,x} + I\epsilon_{w(t),x}}$ and $R_{t,y} = e^{\alpha_{0,y} + I\epsilon_{w(t),y}}$. Using the above mentioned relative approach in the counterfactual scenario where $x$ and $y$ are the donor and the recipient countries respectively, we have $R_{t,x\to y} = e^{\alpha_{0,x} + I\epsilon_{w(t),x}}$ if $t < t_{start}$ and $R_{t,x\to y} = e^{\alpha_{0,x}}/e^{\alpha_{0,y}} .\ e^{\alpha_{0,y} + I\epsilon_{w(t),y}}$ if $t \geq t_{start}$. After canceling the term, we obtain $R_{t,x\to y} = e^{\alpha_{0,x} + I\epsilon_{w(t),x}}$ if $t < t_{start}$ and $R_{t,x\to y} = e^{\alpha_{0,x} + I\epsilon_{w(t),y}}$ if $t \geq t_{start}$.

Using Equation (R5), $R_{t_{start}, y\to x}$ and fitted true infections for $\tau < t_{start}$, we obtain estimates of $c_{t,x}$ for all $t \geq t_{start}$. Finally the estimates of $d_{t,x}$ and $d^*_{t,x}$ are simulated for the counterfactual situations putting the estimates of $c_{\tau,x}$ in Equations 3 and 4 respectively.

**We thank Reviewer 1 for being satisfied with the revised version of our manuscript. Next we provided responses to the questions 4,5,6 of the first revision posed by Reviewer 2.**

1. **Authors opted the mobility baselines, calculated as the median value for the corresponding day of the week during January 3, 2020 - February 6, 2020. What is the rationale to consider such base line? Better to mention it clearly in the text with evidence of supports. How this baseline for one month could represent the other timing of the year? By January 2020, the human mobility behavior already started changing even though the Governments had not implemented any specific intervention and travel restrictions. Authors could consider the baseline for each week of a year from the data during earlier years. I have not seen any sensitivity analysis on these baselines over the counterfactual results and hence the final outcomes.**

**Response :** We thank the reviewer for this comment. In our original and primary counterfactual analysis for both reported and estimated total deaths, **we did not use mobility as a variable in the semi-mechanistic transmission models.** Instead the six measures of mobility reported in the previous Results Section were solely used **as descriptive statistics** to set the backdrop for variability of factors that may influence the pandemic control dynamics and ultimately lead to fluctuations in effective reproduction number ($R_{t,m}$). Many papers have used a hybrid mechanistic model with regression on multiple covariates for the reproduction number, but we **did not pursue** that path in our initial and primary analysis.

Second, the reviewer asked us to perform a sensitivity analysis of how the counterfactual results are varying by changing the time period used for the baseline mobility. Even if we did use mobility as a predictor of ($R_{t,m}$), the Google mobility data is based on a specific baseline established during a specific period (Jan 3–Feb 6, 2020) and represents changes in mobility for each country relative to this baseline (see the Google COVID-19 Community Mobility Reports).

This baseline **was adopted by Google not by us.** This granular mobility data was released by Google to aid in mitigating the impact of COVID-19, there is **no pre-2020 data** available for these countries. For all the five South Asian countries of interest in our analysis, the mobility data were available starting from **February 15, 2020.** Therefore **it is not possible to change the baseline period** to dates before 2020 as suggested by the reviewer due to lack of data availability.

However, in response to the reviewer comment, we performed a **sensitivity analysis** to infer how different the counterfactual results will be affected if we included mobility as a predictor of ($R_{t,m}$). For this analysis, we just concentrated on the first half of 2021 from Jan 1, 2021 - June 30, 2021 using reported mortality data only. We added the following text in the Sensitivity Analysis Section:

“In equation R6, we postulated the model for the time varying reproduction number $R_{t,m}$ as a random walk process. One can incorporate other covariates for example mobility into the $R_{t,m}$ model, treating this as a regression model with correlated error structure. We defined $mob_{t,m}$ as the sum of the six types of variables available from mobility data at day $t$ for country $m$, where each of these variables have been standardized with respective means and standard deviations. In order to incorporate mobility data into the transmission model we modified the $R_{t,m}$ model as

$$log(R_{t,m}) = \alpha_{0,m} + \beta mob_{t,m} + I\epsilon_{w(t),m}$$

One can also use country specific $\beta_m$ in the above equation. Before fitting the above model we standardized the mobility data with respective mean and standard deviation for each country. Since there is no information available regarding the prior value for the coefficient of mobility, $\beta$, we compared counterfactual results using two different choices of means of the assumed prior distributions, specifically $N(log(2), 0.1)$ and $N(log(1.5), 0.1)$.

In our original analysis, the counterfactual results in the first half of 2021 (Figure R3) indicate that Pakistan and Nepal undertook the best measures followed by Bangladesh and India whereas Sri Lanka performed the worst among the five countries. In contrast, the counterfactual results from the mobility analysis (Figures R4 and R5) suggest that the pandemic performances of Pakistan and Bangladesh were inferior compared to the findings of the original analysis. Overall from the mobility analysis, Nepal and India appear to take the best measures followed by Sri Lanka. Pakistan and Bangladesh could have averted a significant number of deaths due to COVID-19 in all the counterfactual situations where they were the recipients. The counterfactual mortality curves exhibit an identical pattern for both prior mean values of the coefficient of mobility, $\beta$ in the $R_{t,m}$ model with the only distinction being that the peaks of the curves associated with a prior mean value of 2 are greater than those corresponding to a value of 1.5 (Figures R4 and R5). However, the relative ranking of the countries in terms of pandemic performance remains the same if we change the prior distribution parameters.

| Country | With Vs Without Mobility | With Mobility Vs EpiEstim* | Without Mobility Vs EpiEstim* |
|---|---|---|---|
| India | 0.86 | 0.86 | 0.74 |
| Bangladesh | 0.82 | 0.84 | 0.65 |
| Pakistan | 0.64 | 0.46 | 0.26 |
| Nepal | 0.99 | 0.27 | 0.26 |
| Sri Lanka | 0.72 | 0.73 | 0.48 |

Table R1 : **Comparison between different $R_{t,m}$ estimates for all the countries in the first half of 2021 (Jan 1, 2021 - June 30, 2021)** . $Estimated\ R_{t,m}$ series are obtained using three ways : Not including mobility (original analysis- only based on reported mortality data), including mobility into the model and finally using the R Package EpiEstim* which calculates $R_{t,m}$ based on COVID-19 reported cases data. * EpiEstim using the method "parametric_si" and specifying the mean and sd (4 days and 1 respectively) for the Delta strain of COVID-19.

Figure R2 illustrates the percentage variations in mobility across six distinct categories, specifically Transit Stations, Retail and Recreation, Grocery and Pharmacy, Parks, Workplaces, and Residential, for the year 2021 within the context of all five countries. Nonetheless, one must exercise caution when drawing conclusions. Figure R2 underscores the significant influence of percent changes in mobility on the counterfactual results derived from this mobility analysis. Notably the countries with higher percent changes in mobility, specifically Bangladesh and Pakistan in this context, exhibited relatively poorer pandemic outcomes compared to other nations. This approach of incorporating mobility into the $R_{t,m}$ model makes it quite driven by the mobility data. There may be other variables like mobility that play a role in determining the $R_{t,m}$ series. Small changes in values of $R_{t,m}$ can lead to large changes in counterfactual death estimates so we should be cautious while using covariates in this model.

Table R1 shows there is a high positive correlation between $R_{t,m}$ estimates with or without mobility. The correlation with the *EpiEstim* estimates (based on COVID-19 reported cases data) is higher for the $R_{t,m}$ estimates from the original analysis without mobility. In light of these considerations, we present the findings derived from the original analysis in the main text and mention the mobility analysis as a part of our sensitivity analysis.

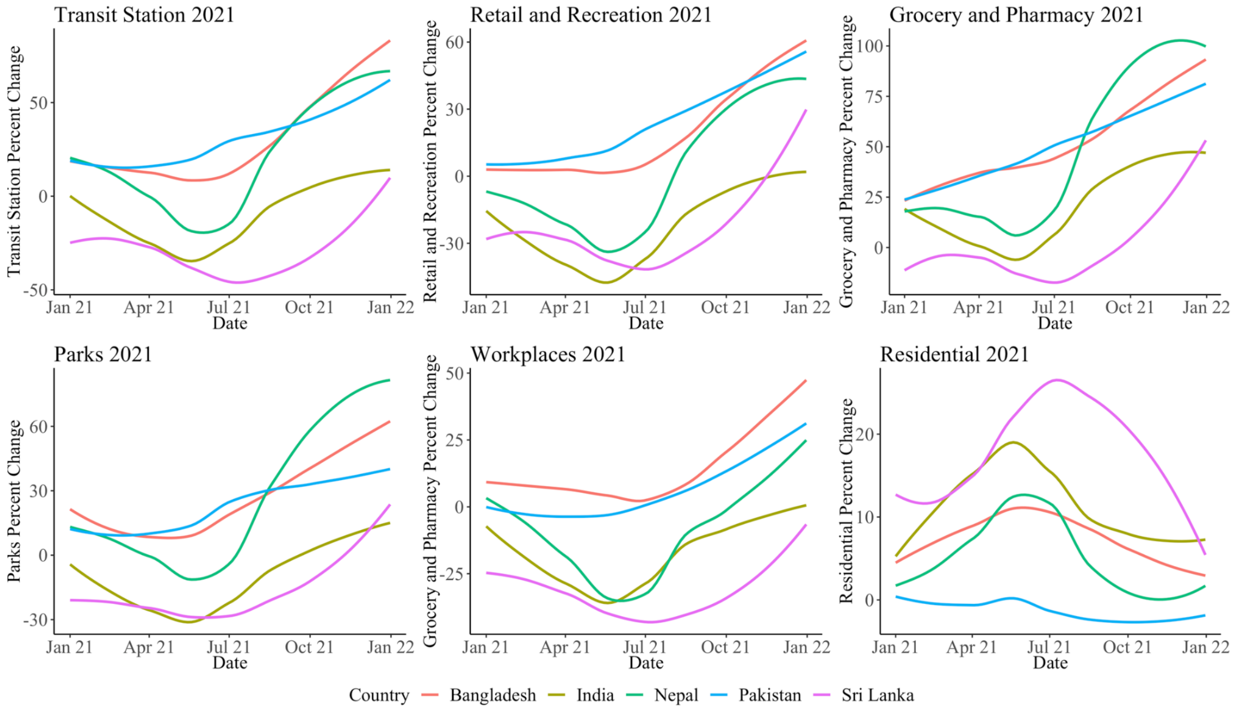


**Figure R2 : Daily Percent change from baseline for different mobilities in 2021.** Baseline is the median value for the corresponding day of the week during January 3, 2020 - February 6, 2020. Percent changes are reported for Transit Station, Retail and Recreation, Grocery and Pharmacy, Parks, Workplaces, and Residential for the year 2021. Google Mobility data are used for this plot.

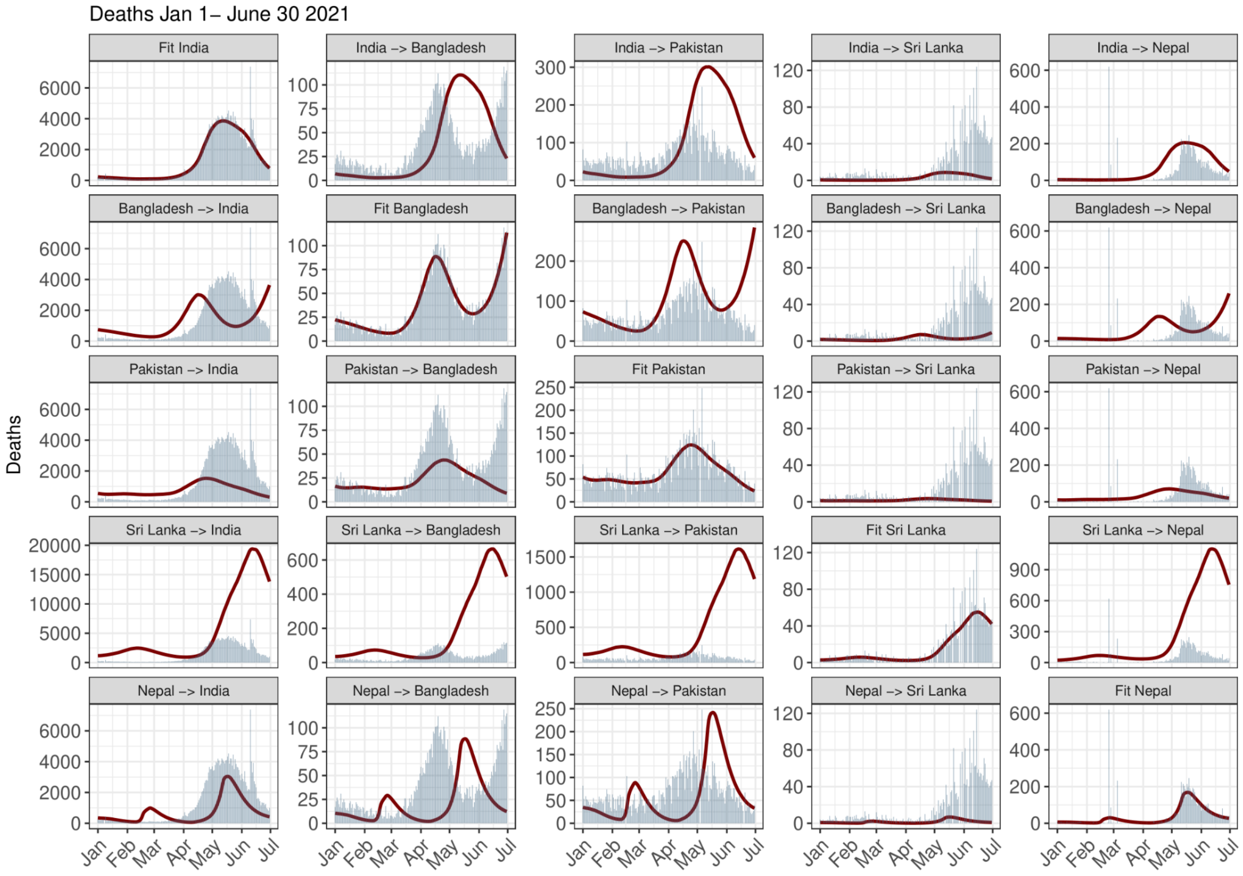


**Figure R3: Daily reported deaths for the different counterfactual situations along with the actual fitted deaths for each country in the first half of 2021.** Recipient countries vary along the columns while the donor countries vary along the rows. The blue bars in the plots denote the actual daily death cases for the recipient country, while the red lines denote the counterfactual ones. The time period of analysis is from Jan 1, 2021 to June 30, 2021.

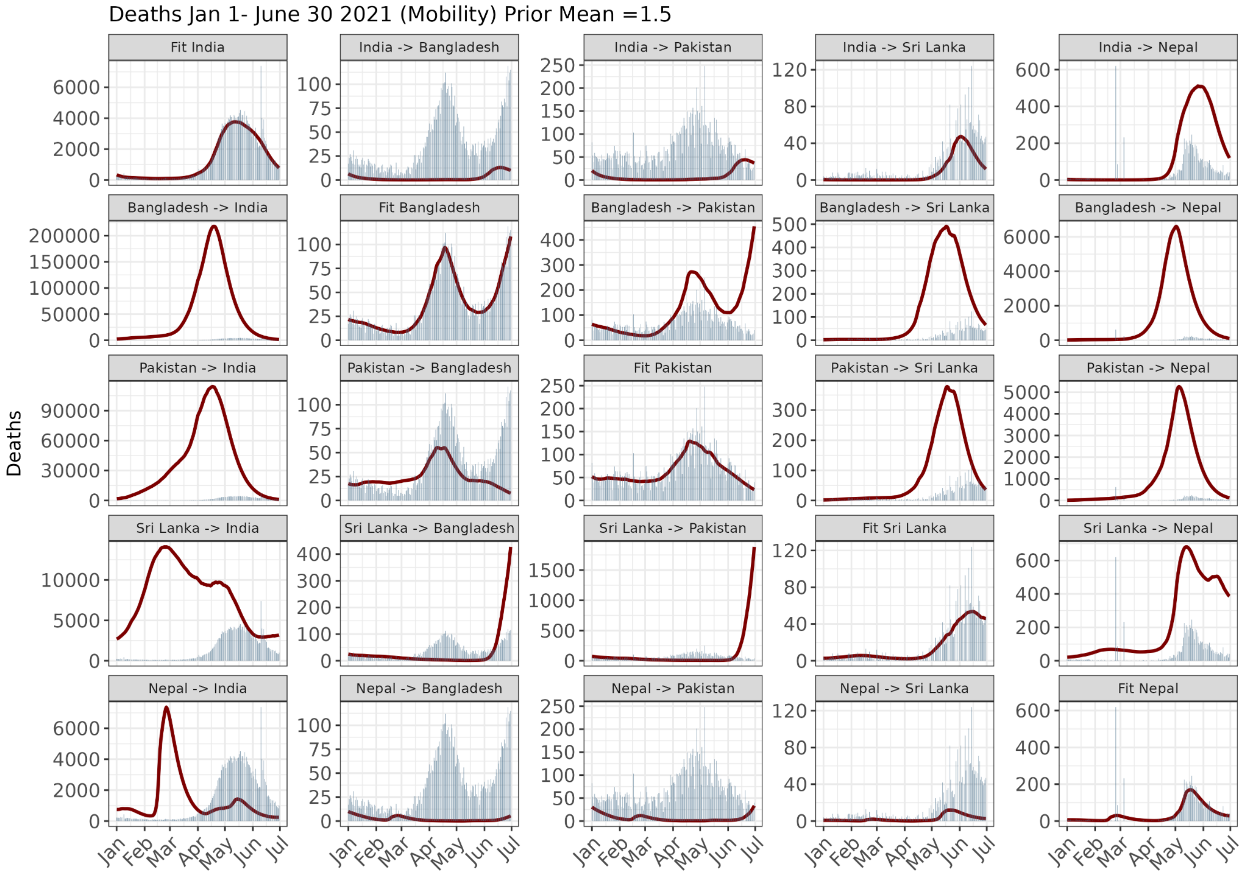


**Figure R4: Daily reported deaths for the different counterfactual situations along with the actual fitted deaths for each country in the first half of 2021 using mobility data in the transmission (Prior Mean =1.5)**. Recipient countries vary along the columns while the donor countries vary along the rows. The blue bars in the plots denote the actual daily death cases for the recipient country, while the red lines denote the counterfactual ones. The time period of analysis is from Jan 1, 2021 to June 30, 2021.

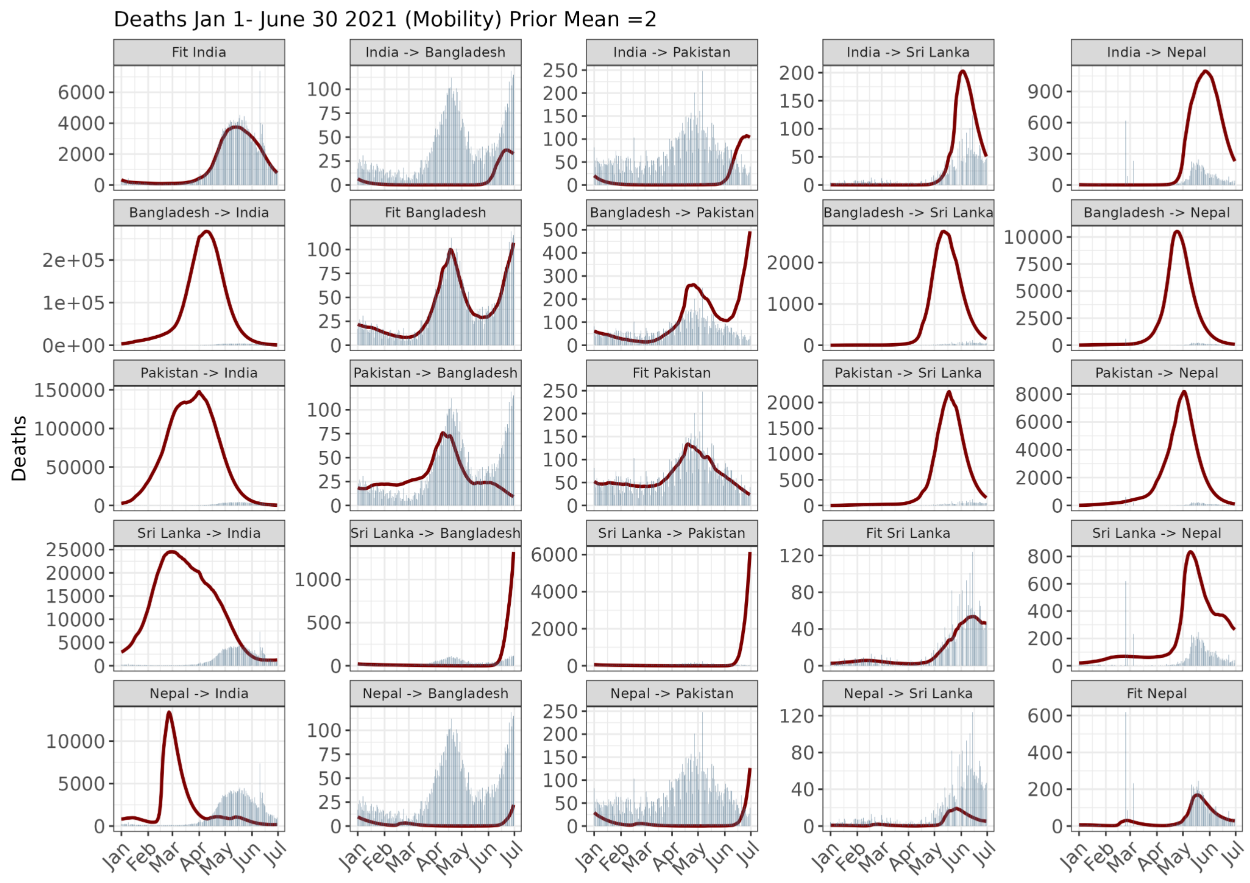


**Figure R5: Daily reported deaths for the different counterfactual situations along with the actual fitted deaths for each country in the first half of 2021 using mobility data in the transmission (Prior Mean = 2)**. Recipient countries vary along the columns while the donor countries vary along the rows. The blue bars in the plots denote the actual daily death cases for the recipient country, while the red lines denote the counterfactual ones. The time period of analysis is from Jan 1, 2021 to June 30, 2021.

2. **In equations 1 and 2, authors used the IFR for reported and total retrospectively and assumed to be constant. While the expected deaths d_t,m was temporally evaluated by introducing the temporal components as daily infection and their density to death from the time of infections as the backward looking.**
   **- First how density (Pi) could be constant across the epidemics, in fact several factors could change the distribution over time? If authors assumed this constant should be clearly mentioned in the text and discuss in the limitation section.**

**Response :** We thank the reviewer for this observation. We have tried to incorporate the response to this question in the restructured Methods section. Please refer to “Linking Mortality

to Infections” under “Renewal Equations” and “Prior Specification on the Infection Fatality Rate (IFR)” under “Specification/choice of priors and other input parameters” in the Model Section.
We added the following part under Sensitivity Analysis in the Results section of the main paper.

**Sensitivity Analysis on IFR prior parameters:**
In our sensitivity analysis, we explored two distinct parameter specifications for the normal prior distribution on the Infection Fatality Rate (IFR), specifically denoted as (mean, sd) equal to (0.05, 0.03) and (0.12, 0.06), respectively. The counterfactual results from both of these parameter specifications (Figures R6 and R7) exhibit precisely the same overall pattern as the original analysis (as illustrated in Figure R2). The only numerical difference lies in the magnitude of the peak in the curves, which is inflated in the first specification and deflated in the second. It is important to reiterate that our counterfactual analysis maintains a relative perspective. Therefore, the primary relative rankings of countries in terms of their pandemic performance have remained consistent throughout this sensitivity analysis even if changes are observed for the actual counts of mortalities in these counterfactual settings.
The serosurvey estimates in India were roughly at a six months interval. During the period of 2020 and 2021, four serosurveys were conducted in India. The 95% confidence intervals corresponding to the assumed IFR prior distributions contained the estimates obtained from these serosurveys. Moreover, both the serosurveys in 2021 provided similar IFR estimates. *Sharma et al. (2021)* [7] performed a sensitivity analysis for time varying IFR vs. Fixed IFR where they used a similar Bayesian Semi-Mechanistic Model. They observed that the results using time varying IFR did not deviate significantly from the ones obtained using fixed IFR. They obtained the data for time varying IFR in the UK using the office for national statistics (ONS) data. However, such detailed COVID-19 data were unavailable for the five countries of interest.

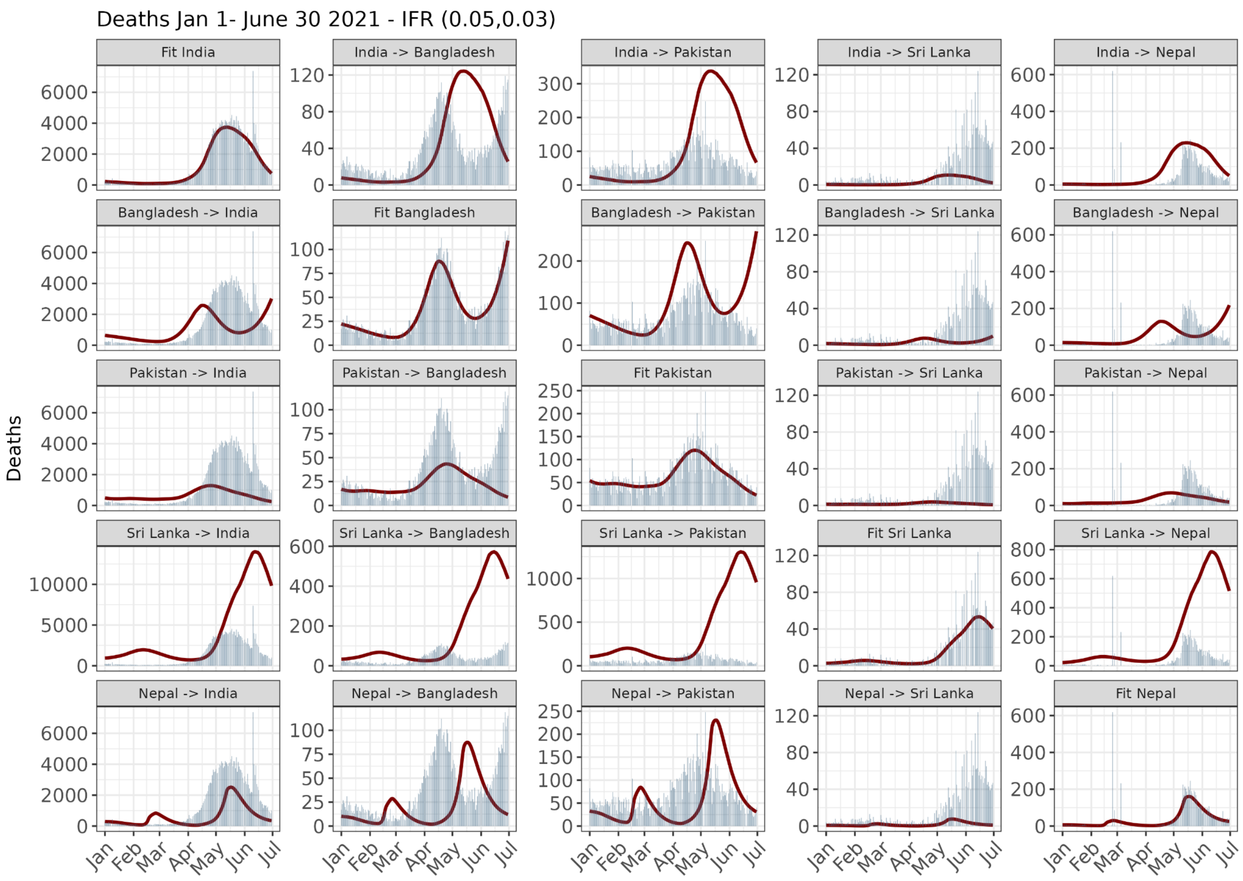


**Figure R6: Daily reported deaths for the different counterfactual situations along with the actual fitted deaths for each country in the first half of 2021 where the (mean,sd) for the IFR prior is given by (0.05,0.03)**. Recipient countries vary along the columns while the donor countries vary along the rows. The blue bars in the plots denote the actual daily death cases for the recipient country, while the red lines denote the counterfactual ones. The time period of analysis is from Jan 1, 2021 to June 30, 2021.

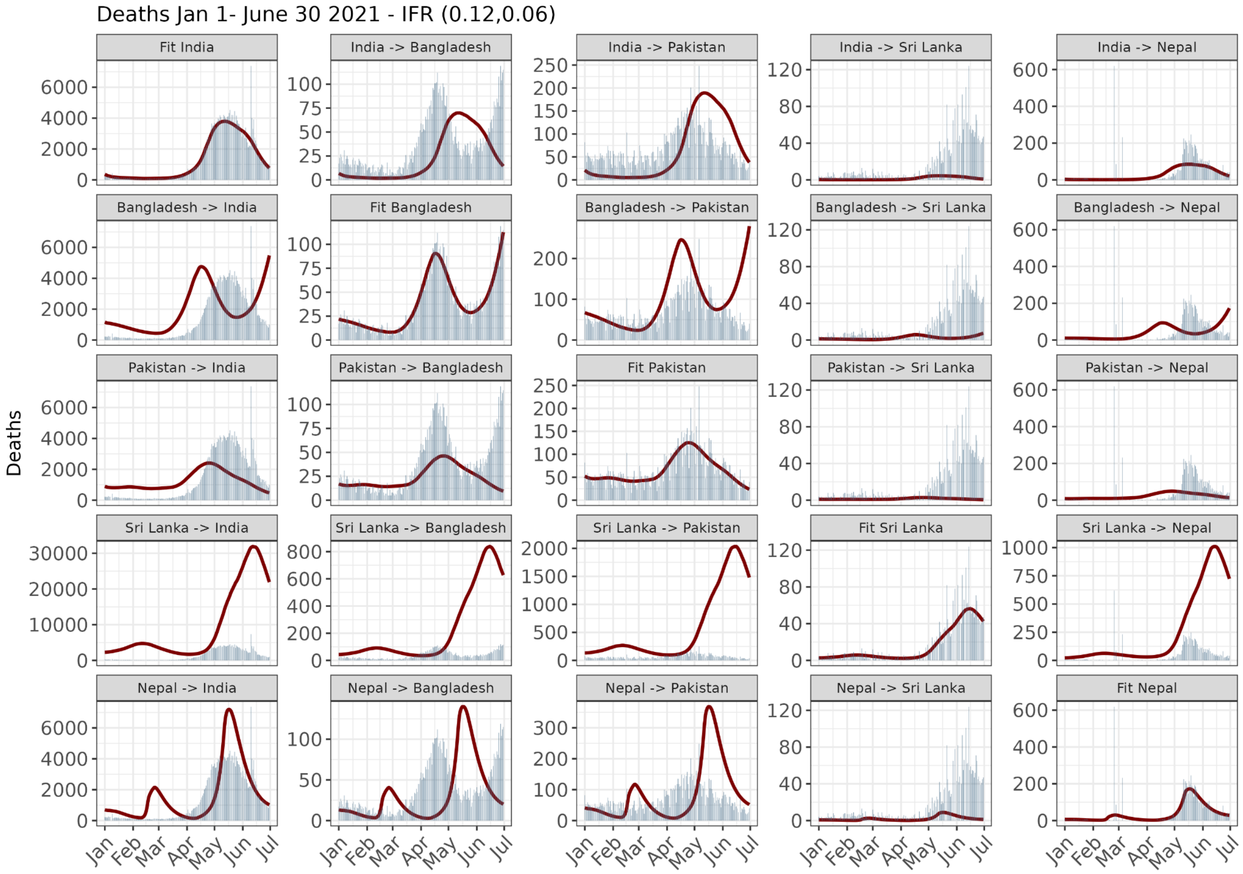


**Figure R7: Daily reported deaths for the different counterfactual situations along with the actual fitted deaths for each country in the first half of 2021 where the (mean,sd) for the IFR prior is given by (0.12,0.06)**. Recipient countries vary along the columns while the donor countries vary along the rows. The blue bars in the plots denote the actual daily death cases for the recipient country, while the red lines denote the counterfactual ones. The time period of analysis is from Jan 1, 2021 to June 30, 2021.

- **Authors have rightly considered the unobserved cases in the formulation, but I could not find how they have estimated the unobserved cases over time and not clear in the text. Please clarify or provide the details.**

**Response :** We thank the reviewer for this inquiry. For this response at first refer to "Linking Mortality to Infections" and "Linking Current Infections to Past Using Reproduction Number" under "Renewal Equations" in the Model Section.

**We added the following part in the Supplementary Section S1**

**Infection Modeling**

**Priors for initial seedings:**

We seeded the transmission model with six sequential days of an equal number of infections, $c_{1,m} = c_{2,m} =... = c_{6,m} \sim Exp(1/\tau)$ where $\tau \sim Exp(1)$. The prior parameter was chosen based on model convergence. For the counterfactual death analysis such a prior assumption is reasonable since it is a relative analysis. Its impact is analogous to what we observed when varying the prior values of the Infection Fatality Rate (IFR) mean in the Sensitivity Analysis (as discussed in the Results Section). Nonetheless, the posterior estimates of $c_{t,m}$ obtained on the basis of this assumption, particularly for the year 2021, are deemed unreliable. Consequently, we have adopted an indirect approach to address this issue.

During the period of 2020-2021, four national serosurveys were conducted in India to estimate the true prevalence of COVID cases in India. These serosurveys provided estimates of Under Reporting Factor for cases ( $URF_{cases}$) where,

$URF_{cases} = Cumulative\ number\ of\ true\ infections/Cumulative\ number\ of\ reported\ infections$

in India, specifically 29.1 (May-June 2020), 14.3 (August-September 2020), 25.7 (December 2020- January 2021) and 24.9 (June-July 2021). The denominator of $URF_{cases}$ is readily available from JHU data source (https://coronavirus.jhu.edu/data). With the help of sero estimates we obtain estimates of the numerator for India at the end of each serosurvey period. We predicted the $URF_{cases}$ at other dates for India in the period of March 15, 2020- Dec 31, 2021 using linear interpolations with knots at the known serosurvey end dates. In terms of the notation of the transmission model, the numerator in $URF_{cases}$ at day $t$ is equal to $\sum_{\tau=0}^{t} c_{\tau,m}$ where $m$ stands for India. Using the model estimates of $c_{\tau,m}$ and the ones obtained from serosurveys, we calculated the factor by which the model infection estimates needed to be adjusted to match the serosurvey estimates for India.

To obtain the estimates of $URF_{cases}$ for the other four countries, we made an assumption that the adjusting factor obtained for India remained the same for the other countries as well. We report these modified estimates of $URF_{cases}$ for 2020 and 2021 in Tables R1 and R2 respectively. **All these estimates are obtained using reported deaths analysis only.**

| Countries | June 1 | Sep 1 | Dec 31 |
|---|---|---|---|
| **India** | 29.1 | 14.1 | 25.0 |
| **Bangladesh** | 13.5 [7.7, 29.4] | 10.6 [6.3, 21.0] | 32.3 [19.8, 59.8] |
| **Pakistan** | 20.4 [11.7, 43.8] | 14.9 [8.6, 30.6] | 45.4 [27.2, 86.9] |
| **Sri Lanka** | 2.3 [0.7, 6.9] | 2.2 [0.6, 7.4] | 15.5 [7.2, 41.0] |
| **Nepal** | 5.2 [2.2, 14.2] | 7.6 [4.2, 16.8] | 16.0 [9.6, 29.7] |

**Table R2: Table for Under Reporting Factors (URF) along with their 95% CI for Cases over the one year period starting from March 15, 2020 to Dec 31, 2020 for the different countries.** For each country, we report the URF for cases on June 1, September 1 and December 31.

| Countries | April 1 | July 1 | Oct 1 | Dec 31 |
|---|---|---|---|---|
| **India** | 25 | 25 | 25 | 25 |
| **Bangladesh** | 25.3 [25.2,25.4] | 30.5 [30.1,30.9] | 30.9 [30.1,31.8] | 30.3 [29.9,31.5] |
| **Pakistan** | 23.3 [23.1,23.5] | 27.4 [26.8,28.2] | 30.0 [29.1,31.2] | 30.7 [30, 32.1] |
| **Sri Lanka** | 17.1 [17.0,17.3] | 17.3 [16.8,18.0] | 16.6 [15.6,18.0] | 15.8 [14,17.4] |
| **Nepal** | 25.8 [25.7, 25.9] | 29.3 [28.8.30.0] | 29.1 [28.3,30.2] | 28.6 [28.0,30.0] |

**Table R3: Table for Under Reporting Factors (URF) along with their 95% CI for Cases over the one year period starting from Jan 1, 2021 to Dec 31, 2021 for the different countries.** For each country, we report the URF for cases on April 1, July 1, Oct 1 and December 31.

In 2020, Pakistan had the highest $URF_{cases}$ of 45.4 [27.2, 86.9] on December 31, 2020. In contrast, Sri Lanka and Nepal had consistently lower URF compared to the other three countries. In 2021, except Sri Lanka all the other four countries had comparable $URF_{cases}$ around 25-30. Throughout this work, our main focus is on comparing the five countries based on their performances in the counterfactual scenarios in terms of mortality. The $URF_{cases}$ estimates provided in Tables R1 and R2 should be used with caution since these are based on several assumptions.

3. **The selling part is the ‘linking Rt to the mortality’. I found this part is not clear enough.**
   **- Authors used the renewal process to estimate Rt from daily number of cases. Where generation time distribution is approximated with the serial interval distribution as g ~ Gamma (6.5, 0.62), but not cited the evidence for this parametric distribution. I wonder authors have evaluated from the line-list data for each country. Please clarify or revise the text with the relevant information**

**Response :** We thank the reviewer for the feedback and agree that we needed to improve our explanation of the model description.

We would like to clarify first that the renewal equation used in our work doesn’t use the daily cases to estimate the time varying reproduction number but rather uses the **daily deaths** to estimate it. This can be seen from equations R3-R5. In a nutshell our formulation uses infections (not just positive cases- this is basically true infections) as a latent variable and then links infections to deaths via Equation R3 or R4. Then the input data in the model are daily deaths as illustrated in Equations R3 and R4. Next please refer to “**Generation Time Distribution” under “Specification/choice of priors and other input parameters”**

We performed a sensitivity analysis to compare the counterfactual results by varying the mean parameter of the assumed Gamma distribution for the generation time. *Xu et al. (2023)* [8] inferred that the mean intrinsic generation times for Alpha, Delta and Omicron variants are 5.86 [5.47-6.26], 5.67 [3.79-7.55] and 6.84 [5.72-8.6] respectively. Therefore in the sensitivity analysis we chose the means of the Gamma distribution for generation to be 5, 6 and 7 days respectively with sd 4.2 which covers the generation times for all the three variants of COVID.

The counterfactual outcomes exhibited minimal variation upon adjusting the mean generation time, as depicted in Figures R8, R9, and R10. The alterations primarily manifested as slight adjustments in the actual mortality counts. Notably, the relative ranking of the five countries concerning their pandemic performances remained consistent with the original analysis.

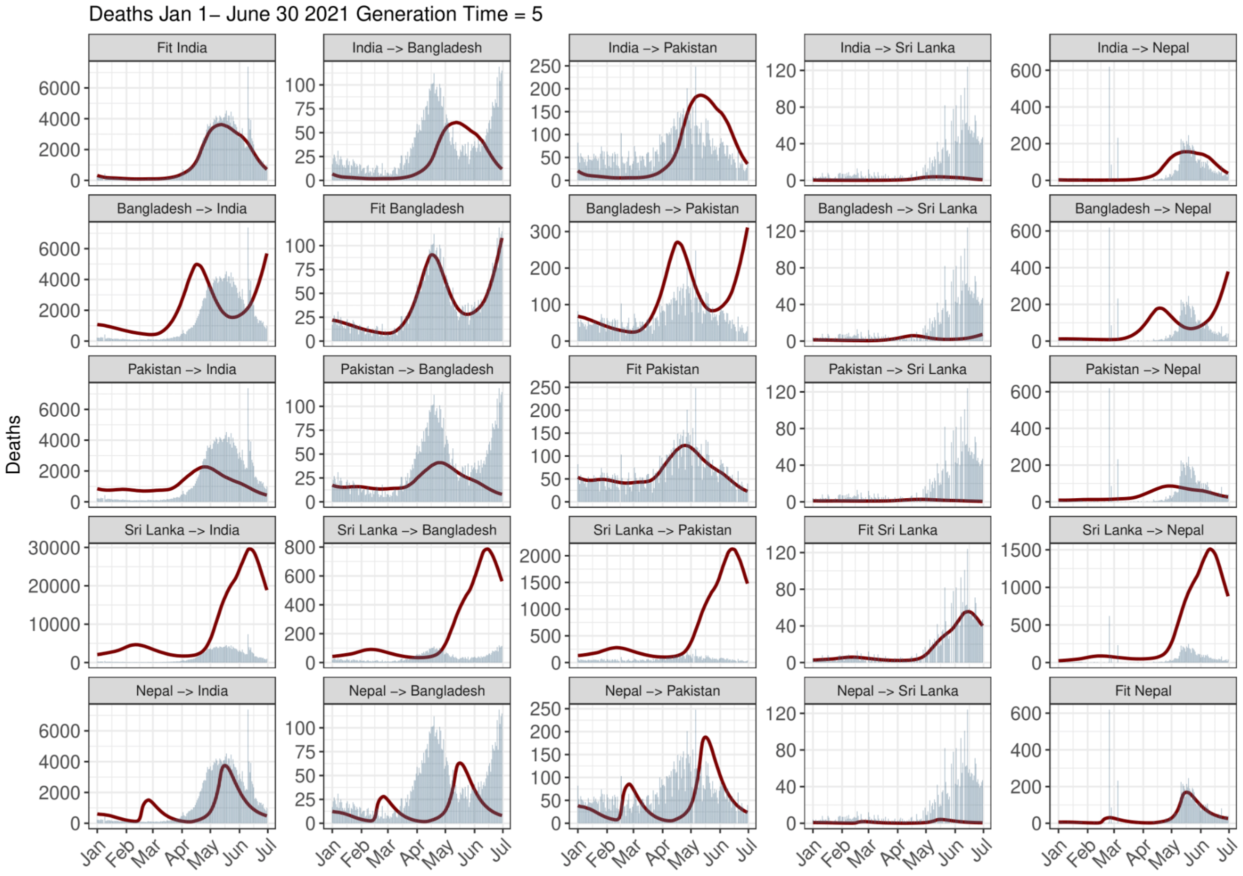


**Figure R8: Daily reported deaths  for the different counterfactual situations along with the actual fitted deaths for each country in the first half of 2021 where the mean for the generation distribution is 5 days**. Recipient countries vary along the columns while the donor countries vary along the rows. The blue bars in the plots denote the actual daily death cases for the recipient country, while the red lines denote the counterfactual ones. The time period of analysis is from Jan 1, 2021 to June 30, 2021.

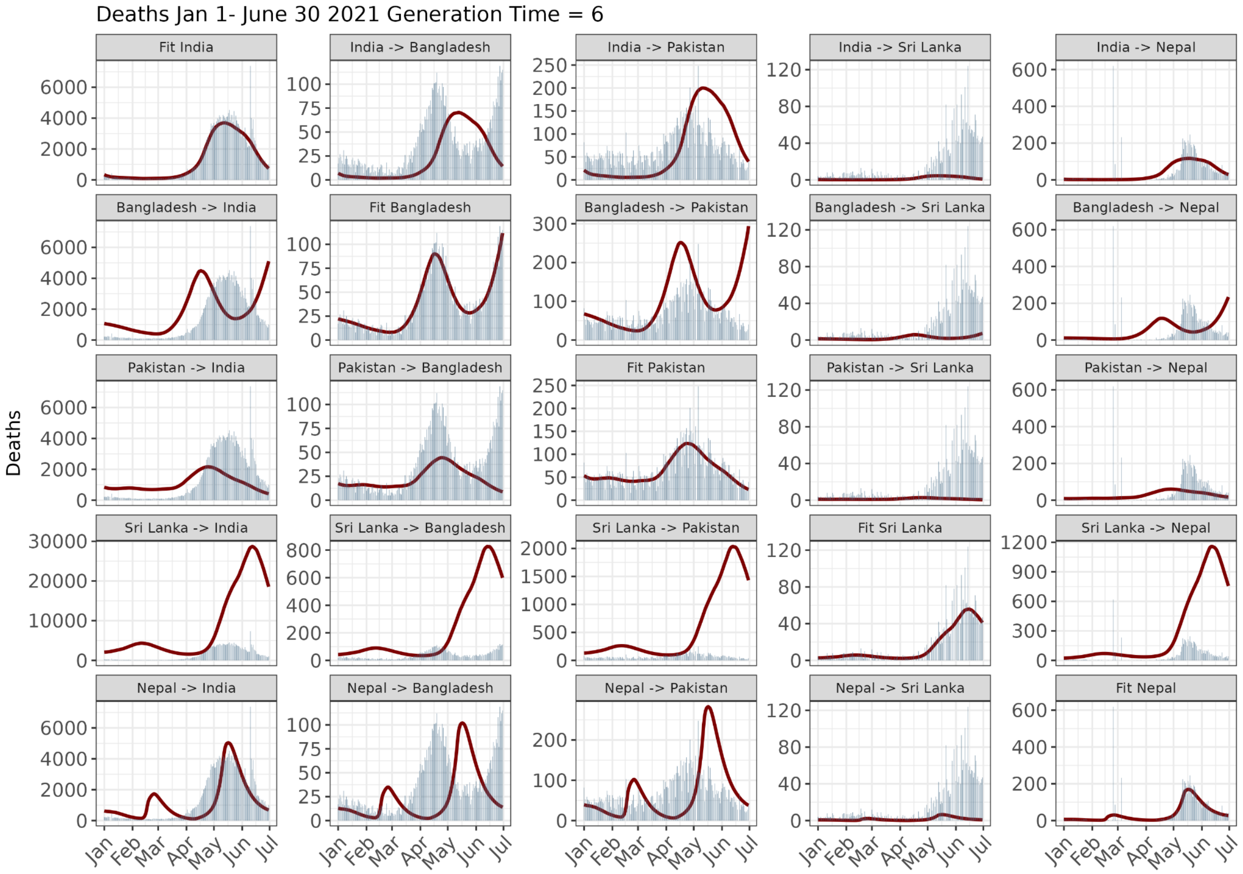


**Figure R9: Daily reported deaths for the different counterfactual situations along with the actual fitted deaths for each country in the first half of 2021 where the mean for the generation distribution is 6 days**. Recipient countries vary along the columns while the donor countries vary along the rows. The blue bars in the plots denote the actual daily death cases for the recipient country, while the red lines denote the counterfactual ones. The time period of analysis is from Jan 1, 2021 to June 30, 2021.

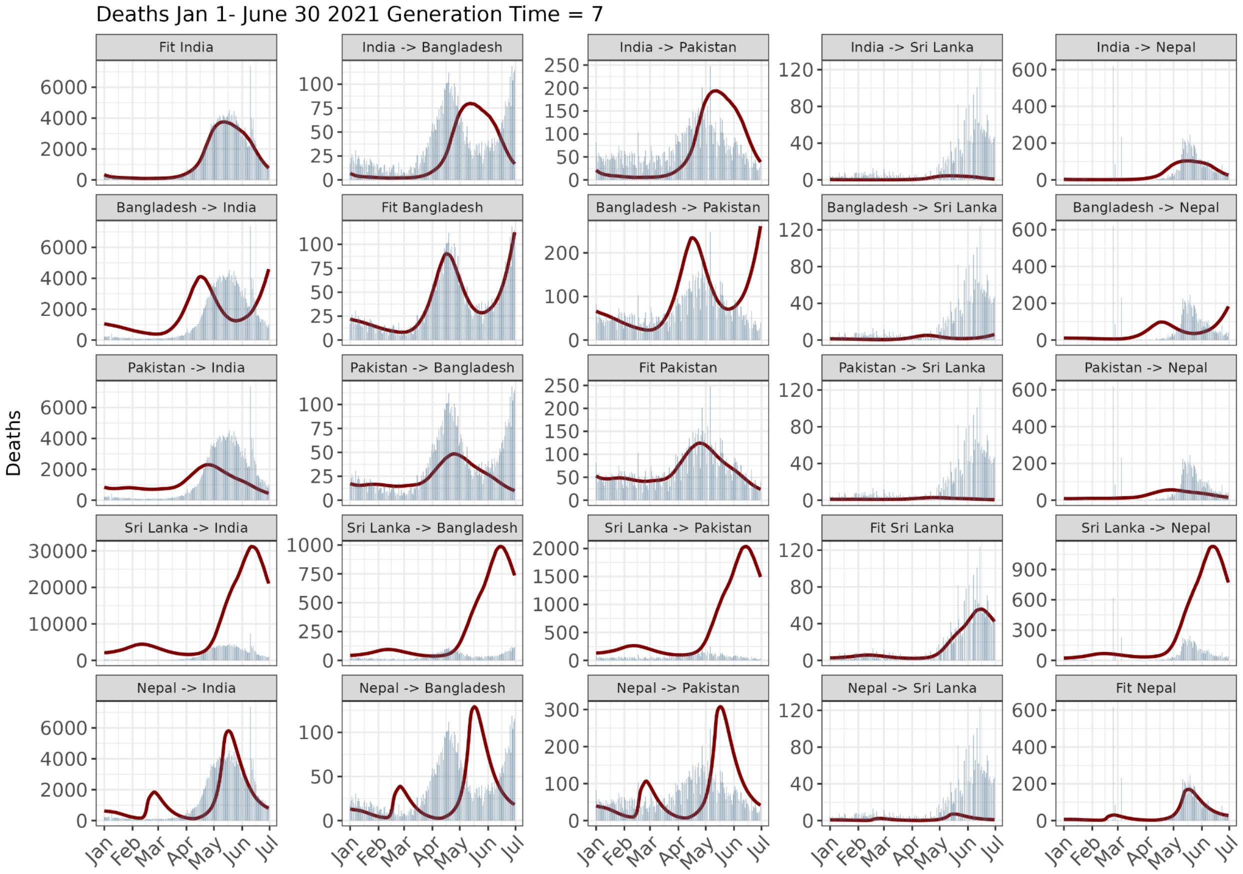


**Figure R10: Daily reported deaths for the different counterfactual situations along with the actual fitted deaths for each country in the first half of 2021 where the mean for the generation distribution is 7 days**. Recipient countries vary along the columns while the donor countries vary along the rows. The blue bars in the plots denote the actual daily death cases for the recipient country, while the red lines denote the counterfactual ones. The time period of analysis is from Jan 1, 2021 to June 30, 2021.

- **Considering the Rt as random process with exponential form is very crude assumption, in fact, the Rt usually considered to follow gamma distribution, which surely an exponential family of distribution but should not be an extreme member of the family. I suggest to provide the evidence for this assumption and cite the original study for this.**

**Response :** We would like to clarify here that we have not assumed any parametric form for the time varying reproduction number . In our formulation, exponentiating the random walk process essentially serves the purpose of restricting the latent random process to be strictly

positive which is different from assuming the $R_{t,m}$ to have an exponential distribution. Therefore we model $log\ (R_{t,m})$ which is unrestricted. Next please refer to "Structure of the model for $R_{t,m}$" in the Models Section.

**We did not use any exponential distribution assumption for the $R_{t,m}$ model instead we just modeled $log(R_{t,m})$ to ensure positivity of $R_{t,m}$. *Quick et al. (2021)* [9] used a similar type of approach to model jointly estimate $R_{t,m}$ , ascertainment rates, incidence, and prevalence over time in one or multiple regions.**

- **The measure Rt is mostly driven by the susceptibility, but while reconstruction via. random process, the authors estimated e^α0,m, which is R0,m the basic reproduction number for country m. Then multiplied a quantity e^Iϵw(t),m, which fixed to 1 at the start of the epidemic and then follow the random walk. I am not sure how it will ensure to have decreasing trend accounting the depletion of susceptible in the population.**

**Response :** We are sorry for the confusion here. However, we would like to clarify that our model accounts for the depletion in the susceptible population explicitly as follows. Please refer to "Structure of the model for $R_{t,m}$" and "Linking Current Infections to Past Using Reproduction Number" under "Renewal Equations" in the Models Section.

**In short we model the unadjusted $R_{t,m}$ as a random process, then we multiply it with the adjustment factor in the renewal equation to account for the population depletion.**

- **Finally, the linking of transmissibility to the mortality is not found in the text, I can't assess this part as it not available in their original article clearly. Please provide the details how these Rt could be translated to the mortality in temporal scale.**

  **Response :** We are sorry for the confusion here. Please refer to "Linking Mortality to Infections" and "Linking Current Infections to Past Using Reproduction Number" under "Renewal Equations", "Structure of the model for $R_{t,m}$" and "Constructing Counterfactual Scenarios" in the Models Section.

# Bibliography


1. Zimmermann, L., Bhattacharya, S., Purkayastha, S., Kundu, R., Bhaduri, R., Ghosh, P., \& Mukherjee, B. (2021). SARS-CoV-2 infection fatality rates in India: systematic review, meta-analysis and model-based estimation. *Studies in Microeconomics*, 9(2), 137-179.
2. Lauer, Stephen A., et al. ``The incubation period of coronavirus disease 2019 (COVID-19) from publicly reported confirmed cases: estimation and application." *Annals of internal medicine* 172.9 (2020): 577-582..
3. Mishra, S., Scott, J.A., Laydon, D.J. et al. Comparing the responses of the UK, Sweden and Denmark to COVID-19 using counterfactual modelling. *Sci Rep* 11, 16342 (2021).
4. Zeng, Kangwei, et al. "Serial intervals and incubation periods of SARS-CoV-2 Omicron and Delta variants, Singapore." Emerging infectious diseases 29.4 (2023): 814.
5. Bi, Qifang, et al. "Epidemiology and transmission of COVID-19 in 391 cases and 1286 of their close contacts in Shenzhen, China: a retrospective cohort study." The Lancet infectious diseases 20.8 (2020): 911-919.
6. Carpenter, Bob, et al. "Stan: A probabilistic programming language." Journal of statistical software 76 (2017).
7. Sharma, Mrinank, et al. "Understanding the effectiveness of government interventions against the resurgence of COVID-19 in Europe." Nature communications 12.1 (2021): 5820.
8. Xu, Xiangyanyu, et al. "Assessing changes in incubation period, serial interval, and generation time of SARS-CoV-2 variants of concern: a systematic review and meta-analysis." medRxiv (2023): 2023-05.
9. Quick, Corbin, Rounak Dey, and Xihong Lin. "Regression models for understanding COVID-19 epidemic dynamics with incomplete data." Journal of the American Statistical Association 116.536 (2021): 1561-1577.
